# Estimating human mobility in Holocene Western Eurasia with large-scale ancient genomic data

**DOI:** 10.1073/pnas.2218375120

**Published:** 2023-02-23

**Authors:** Clemens Schmid, Stephan Schiffels

**Affiliations:** ^a^Max Planck Institute for Evolutionary Anthropology, Leipzig 04103, Germany; ^b^International Max Planck Research School for the Science of Human History, Max Planck Institute for Geoanthropology (formerly known as Max Planck Institute for the Science of Human History), Jena 07745, Germany

**Keywords:** aDNA, prehistory, mobility estimation, Gaussian process regression

## Abstract

The recent increase in openly available ancient human DNA samples allows for large-scale meta-analysis applications. Trans-generational past human mobility is one of the key aspects that ancient genomics can contribute to since changes in genetic ancestry—unlike cultural changes seen in the archaeological record—necessarily reflect movements of people. Here, we present an algorithm for spatiotemporal mapping of genetic profiles, which allow for direct estimates of past human mobility from large ancient genomic datasets. The key idea of the method is to derive a spatial probability surface of genetic similarity for each individual in its respective past. This is achieved by first creating an interpolated ancestry field through space and time based on multivariate statistics and Gaussian process regression and then using this field to map the ancient individuals into space according to their genetic profile. We apply this algorithm to a dataset of 3138 aDNA samples with genome-wide data from Western Eurasia in the last 10,000 y. Finally, we condense this sample-wise record with a simple summary statistic into a diachronic measure of mobility for subregions in Western, Central, and Southern Europe. For regions and periods with sufficient data coverage, our similarity surfaces and mobility estimates show general concordance with previous results and provide a meta-perspective of genetic changes and human mobility.

## Significance

Ancient human DNA (aDNA) extracted from archaeological contexts allows reconstructing past population movements. Previous methods work by calculating proportions of shared ancestry among individuals or groups in order to answer specific, regional research questions. Here, we propose a large-scale algorithm to quantify human mobility through time and space using bulk aDNA data. The algorithm has two core components: i) interpolation of the spatiotemporal distribution of genetic ancestry to obtain a continuous ancestry information field and ii) probabilistic estimation of a spatial genetic similarity surface for each input sample by projecting its ancestry profile into this field. We apply this to thousands of published genomic samples in the last 10,000 y to trace diachronic mobility patterns in Western Eurasia.

All human behavior is spatial behavior, and spatial perception and interaction are deeply rooted in the human mind. Understanding movements in space—mobility—on different orders of magnitude is therefore a major component for understanding human behavior throughout history (1), from the Iceman’s quest through the Ötztal Alps, to the Viking expansion even beyond Medieval Europe, and maybe eventually humankind’s journey to the stars.

Anthropological theory provides different concepts and categories to classify mobility. Mobility can be permanent or cyclical, a group property or individual behavior, and finally motivated by economic, social, or cultural incentives. It has complex implications for the formation, perception, and interaction of identity (2–4). Migration is an especially challenging and controversial topic (5, 6) as it is notoriously difficult to prove and to uncover its causes among the interdependencies of microprocesses and macroprocesses (7). Narratives of migration are particularly vulnerable to political instrumentalization (8).

The field of archaeogenetics now provides a perspective on mobility, which is at its very core influenced by population genetics theory. The emergence, change, and distribution of human ancestry components—mediated by the mobility of their hosts—are in fact some of its most important research questions (e.g., refs. 9–11), causing fruitful and corrective friction with the humanities (12–14). While so far much archaeogenetic research focuses on particular cultural–historical contexts, the recent growth of published ancient DNA samples from all around the world enables a unique category of quantitative meta-analysis.

Large, explicitly spatiotemporal datasets have been part of population genetics research for a long time already (15), sometimes even with a focus on mobility quantification (16–19). But to our knowledge, only few attempts have been made to systematically derive a continuous, large-scale and diachronic measure of human mobility with ancient genetic data. These are most notably a pioneering publication by Loog et al. (20) and another approach by Racimo et al. (21). Loog et al. measure mobility in prehistoric Europe by comparing the distance matrix correlation among spatial, temporal, and genetic distance for aDNA samples in moving 4,000-y windows. As a result, they generate an unscaled mobility proxy curve that indicates elevated levels of mobility correlating with the Neolithic expansion, the Steppe migration, and, finally, the European Iron Age. Racimo et al., on the other hand, employ admixture analysis to model the dynamics of specific ancestry components through time: Mesolithic hunter-gatherers, Neolithic farmers with ancestry originating in the Near East, and Yamnaya steppe herders, arriving in Europe during the third millennium BC. They derive mobility as a wave front speed of surpassed ancestry component thresholds. To overcome sample sparsity and to correlate the arrival of certain ancestry components with biogeographic metrics, they use Gaussian process regression for the interpolation of relative ancestry component occurrence—an idea we also took as a starting point for our proposed mobility estimation method.

In this paper, we present an algorithm to estimate past human mobility on the individual level. For each individual, we determine a probability distribution in space, which yields locations of likely genetic similarity to the sample in question. We call this the similarity probability surface, which, as we show, is generally informative on where an individual’s ancestors might have lived. The distance between the location where an individual was buried and a point of maximum likelihood in the similarity surface serves us as a simplified proxy for personal mobility in an individual’s (or their ancestors’) lifetime. We apply this algorithm to several thousand previously published ancient genomes from Western Eurasia dating from between 8000 BC and 2000 AD (excluding modern genomes) taken from the Allen Ancient DNA Resource (AADR) (22). And, we show that, while the average results largely match expectations including known and large-scale movements at the beginning and end of the Neolithic, these large-scale patterns are accompanied by considerable individual-level heterogeneity.

## Results

### Interpolating Genetic Ancestry Through Space and Time.

**Fig. 1.**
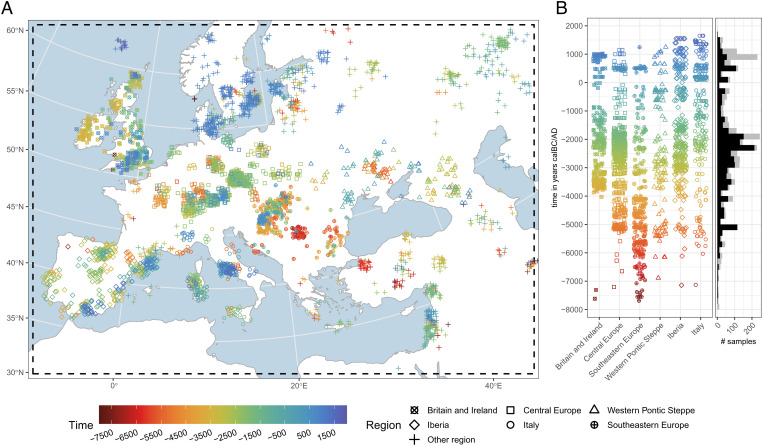
Spatial and temporal distribution of the aDNA sample selection. (*A*) Map (EPSG:3035, ETRS89 Lambert Azimuthal Equal-Area, “European grid") with the research area (dashed). Samples are jittered with up to ±60 km in x and y directions to reduce the effect of overplotting. The sample dots are colored according to their age, which is given in years calBC/AD (negative values indicate ages calBC). The sample dot shape encodes the attribution to different analysis regions. (*B*) Horizontally jittered scatter plot of temporal sample distribution for each analysis region. The stacked histogram on the right shows the sample count through time for all samples in gray and for the ones within the defined analysis regions in black (bin width = 200 y).

A key challenge for understanding shifts of ancestry through space and time is the inherent sparsity of archaeogenetic data. To address this, we employed an interpolation technique fitted upon 3138 published samples available in the AADR (22) for Western Eurasia during the Holocene, filtered according to general sample quality criteria (*Methods*). All samples in this public data collection reference single-nucleotide polymorphisms (SNPs) from a panel of about 1.24 million known informative positions (23). Within the derived AADR subset, the data distribution in time and space is heterogeneous (Fig. 1), with generally few data points from the European Mesolithic, significantly more from the Neolithic, then most from the Late Neolithic and Bronze Age, and again less from the Iron Age and later periods. The diachronic amount of data from Great Britain, Iberia, Central Europe, and Southeastern Europe is comparatively high, whereas other regions are less well covered.

**Fig. 2.**
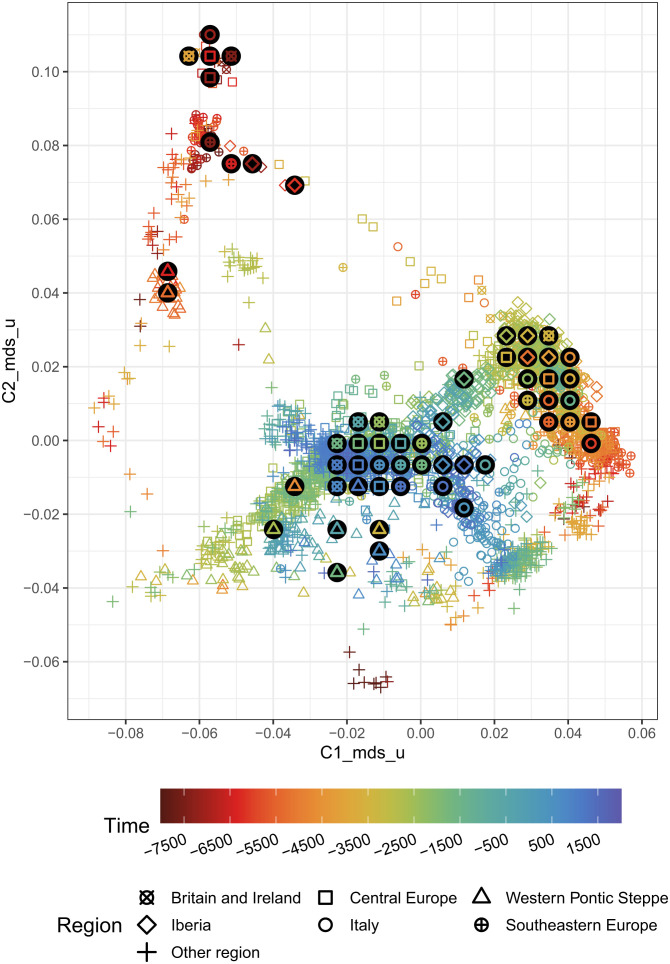
Scatter plot of the sample distribution in 2D multidimensional scaling (MDS) space. Each sample is plotted with the same shape and color as in Fig. 1. The bigger, black circles are the centroids of region-time groups (bin width = 1,000 y). To prevent overplotting, the centroids are not printed on their exact positions, but instead rearranged in a nonoverlapping lattice. *SI Appendix*, Text 1 for an explanation of what *_mds_u exactly entails, *SI Appendix*, Fig. S1 for a larger version where the individuals mentioned in the text are highlighted, and *SI Appendix*, Fig. S4 for a comparable PCA5 plot.

We applied multidimensional scaling (MDS) on these data to reduce their dimensionality to two summarizing ancestry components (Fig. 2), that are by construction most informative about the genetic diversity across the sample set. We use the term “ancestry component” here strictly to denote orthogonal components from MDS instead of a specific admixture component. Besides MDS, we also explored PCA, PCA with projection on modern diversity and EMU (24), considering up to 10 output dimensions for the respective methods (*SI Appendix*, Text 1). We decided to use MDS with two dimensions (MDS2) here and also present Projection-PCA with five dimensions (PCA5) in *SI Appendix*. For MDS2, we find the largest internal separation of samples to be along the tempocultural boundary between the Mesolithic and the Neolithic, highlighting the strong population shift the Neolithic introduced into Europe (25–27). Other patterns seen in the MDS are also consistent with previous observations and will be discussed among our results below.

**Fig. 3.**
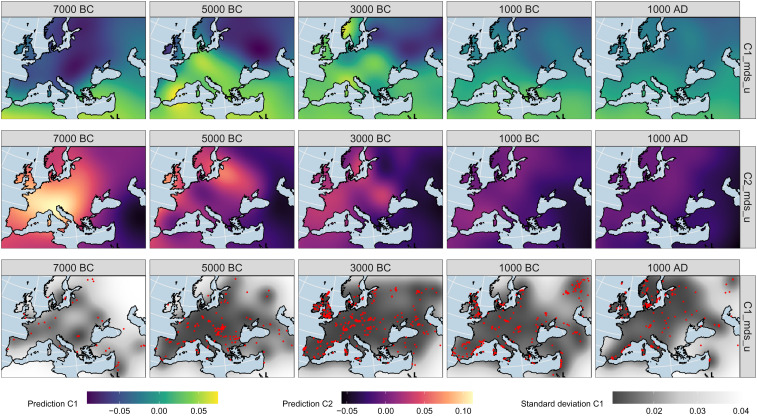
Gaussian process regression interpolation map matrix based on the multidimensional scaling dimensions (resolution = 50 km). The five maps on the top show time slices through the interpolated, spatiotemporal 3D space for the derived ancestry component C1, the five in the middle for C2. The maps on the bottom visualize the standard deviation of the GPR model for C1 (C2 looks nearly identical) and mirror sample sparsity. The samples informing the field from a time window ±1,000 y around the temporal slicing position are plotted here as red dots. *SI Appendix*, Fig. S5 for a comparable PCA5 plot.

We modeled these two MDS-derived ancestry components individually as the dependent variable in a Gaussian process regression (GPR) model with three independent input variables describing the position of each sample in space and time. To learn the properties of the relevant covariance matrix (“kernel”) for a model with the best mean postdiction abilities, we explored multiple methods: variogram analysis, maximum likelihood estimation, and cross-validation. We eventually settled on an anisotropic kernel covering multiple hundred kilometers and years (*SI Appendix*, Text 2). With the parameterized Gaussian process regression model, we predicted an average spatiotemporal genetic ancestry field across Western Eurasia. To illustrate the result, Fig. 3 shows map plots for time slices of this field—a visualization not unlike the seminal work by Menozzi et al. (28), but here leveraging the power of ancient DNA and 1.24 million informative markers.

The interpolation of ancestry components across time and space reflects how 10 millennia of human population changes have shaped genetic ancestry in this area. As already seen in Fig. 2, both ancestry components C1 and C2 most strongly reflect the enormous changes that underlay the transitions during the Early Neolithic, with increasing values (for C1 colored in yellow in Fig. 3) throughout Central and Western Europe before 5000BC as a result of people moving north–westward from the Levant and western Anatolia (25, 26). They also prominently feature further changes after 3000BC, bringing ancestry previously located in Eastern Europe and the Eurasian steppes into Western and Central Europe (9, 29).

With this interpolation, we can attempt the reconstruction of continuous, local ancestry histories even for places without consistent data coverage. To illustrate this, we selected arbitrary spatial positions (corresponding to four capital cities) and used the GPR model to postdict how the genetic profile in these locations changed through time (*SI Appendix*, Fig. S6). The four “virtual” time-series again generally reflect our knowledge of the genetic changes in Europe: In the locations of present-day London and Rome, we observe an ancestry shift with the arrival of Neolithic and then once more with Steppe ancestry—with small regional differences. Riga, on the other hand, starts out with a higher degree of Eastern Hunter-Gatherer ancestry before skipping the influx of the Anatolian farmer component. Jerusalem, expectably, fills a markedly different spot on the genetic map.

### Estimating Individual-Wise Genetic Similarity.

While the interpolated ancestry field reflects the average change in ancestry through space and time, it also forms the basis for our proposed algorithm to understand individual-based mobility. The key idea is as follows (*SI Appendix*, Text 3 for details): Each sample has a coordinate in the multidimensional MDS space, so one value for each output dimension. For a given point in the interpolated, spatiotemporal ancestry field, we can determine the likelihood that exactly this value emerges at that location. If the likelihood is high, so if the “similarity” to said field value is high, and the field point is in the respective past of the sample of interest, then we can deduce that this field point was a potential point of genetic ancestral origin for the sample (*SI Appendix*, Text 4 for a simple toy simulation supporting this assumption). A key feature of this approach is that the likelihood will only be high where the field is sufficiently supported by data (see the standard deviation in Fig. 3). This mitigates effects of extreme data sparsity, e.g., in the periphery of the research area for this study—even if the mean of the interpolated field spuriously aligns to the sample value.

**Fig. 4.**
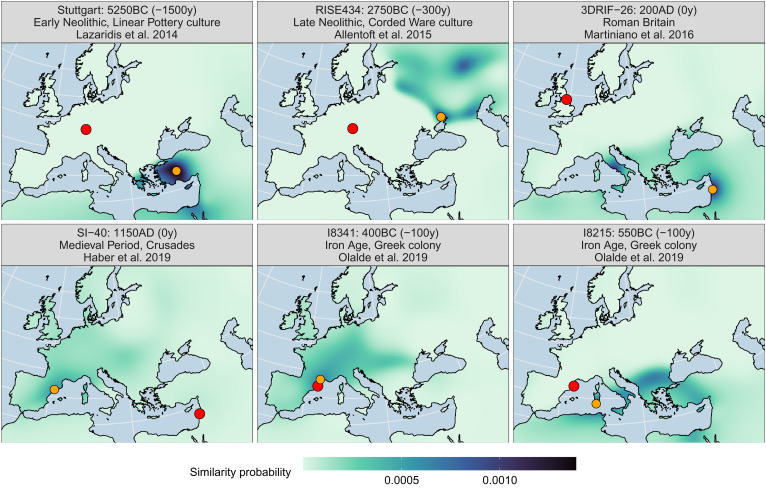
Genetic similarity probability maps for six selected individuals (resolution = 30 km). The larger red dots show their sampling/burial location, and the smaller orange dots the point of maximum probability. The facet labels feature a sample’s identifier, its approximate age, the retrospection distance applied for the search (in parentheses), a general period or context, and the publication where the sample was first discussed. *SI Appendix*, Figs. S8 and S9 for a comparable version of this plot for Projection PCA, which also breaks down the effect of individual output dimensions (C1–C10) for the overall result.

We turn this likelihood of genetic similarity in a given time slice to a normalized probability distribution using Bayes’ formula and multiplying the resulting probabilities for the individual MDS components (*SI Appendix*, Text 3). We prepared such similarity probability maps for six samples from different times, regions, and contexts in Fig. 4. These represent well-understood individuals considered outliers in their genetic signatures and which have been used in the past to establish narratives of mobility and migration. Note the conscious selection of “retrospection” distances, so the temporal distances between the time of death and the interpolated time slice for each sample. This is a key parameter that needs to be tuned to the specific question, as we illustrate below. The figure also features red dots for the sampling/burial locations and smaller orange dots for the respective point of maximum similarity probability in the field.

The individual named Stuttgart, one of the first ancient genomes sequenced (25), is also one of the earliest Neolithic samples from Central Europe. They display nonlocal genetic ancestry in the sense that they differ strongly from preceding Mesolithic samples in the area. In our analysis, we show that indeed the highest similarity probability for this individual can be found in western Anatolia, if we look 1,500 y into their respective past, so to around 6750BC (Fig. 4). This indicates mobility from there to Central Europe in accordance with well-established archaeological models (30). *SI Appendix*, Fig. S7 shows a diachronic sequence of such similarity probability maps for Stuttgart. At around 7500BC, the highest similarity can be observed to the Levant, after 7000BC to Anatolia, then the southern Balkans, and, finally, further North and West. We observe high similarity also to Italy and later Iberia, where the Neolithic expansion followed another route (31, 32). At around 5250BC, so the approximate time of death of the individual, the peak similarity area includes the burial location of the sample itself, demonstrating that this type of ancestry has indeed arrived in Central Europe at this time.

Fig. 4 holds more examples: In the late Neolithic, individuals affiliated with the Corded Ware culture from Central Europe have been identified as among the earliest with so-called Steppe ancestry, which was present already before 3000BC in the Pontic Caspian steppe. Indeed, for a representative sample from that group RISE434 (29) and a retrospection distance of 300 years, we find the closest matching ancestry points falling into Eastern Europe. A third example is an individual from Roman-time Britain 3DRIF-26 (33), buried in York, but featuring a genetic ancestry profile from the Near East. This is a clear case where the original publication concluded that either this individual themselves or their ancestors came from the Near East but ended up in Britain. Besides the high similarity to the Levant, we also observe a peak in the city of Rome, where the field is dominated at the time by many sampled individuals with Near Eastern ancestry (34). Then, confirming the analysis by Haber et al. (35), we find that multiple samples (here SI-40) extracted from a mass burial near a Crusader castle in Sidon in present-day Lebanon are linked to Iberian ancestry profiles (before the Umayyad conquest). Finally, to highlight a case with strongly differing ancestry profiles from the very same site, we show results for the samples I8215 and I8341 from the Iron Age Greek colony of Empúries in northeast Iberia. Empúries presumably had a multiethnic population, where I8215 represents an ancestry group similar to Bronze Age individuals from the Aegean, and I8341 similar to local Iron Age Iberians with some degree of Northern- and Central European ancestry (36). The similarity probability landscapes derived by our algorithm are plausible given these circumstances.

### Regional Mobility Patterns During the Last 10,000 y in Western Eurasia.

We argue that our aforementioned algorithm to derive similarity probability surfaces is a powerful method to visualize the spatial component of the genetic ancestry history of a single individual and to thus gain insights into mobility events happening in their life or the lives of their ancestors. For a large-scale meta-analysis that combines information from many individuals into regional statistics, we now require a simplified summary of the information in the probability surface. We solved that by spanning a spatial “mobility vector” from the burial location to the location of maximum similarity probability in a past time slice. This vector has a length and a direction, which renders it a simple summary that can be visualized along a time series. For example, for the individual from Roman-time York introduced above (3DRIF-26), this means that we infer a vector pointing from York to the hypothesized region in the Levant, resulting in a distance of several thousand kilometers and in south–western direction.

**Fig. 5.**
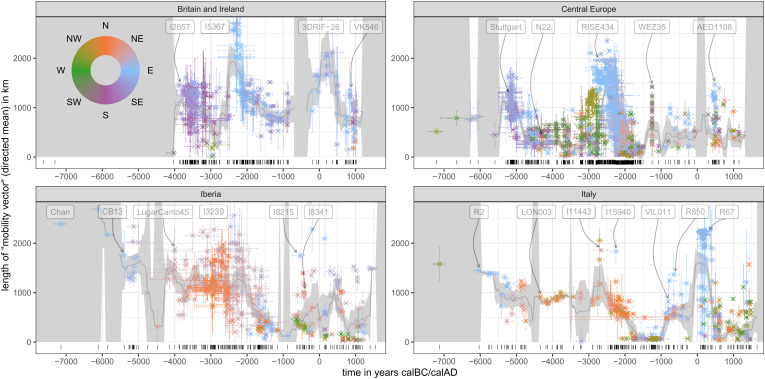
Mobility estimation results. Scatter plots for four analysis regions (Fig. 1). The position on the x-axis gives the median ages of the samples in years calBC/AD. The y-axis and color reflect length and direction of the mean mobility vector of each sample (the vector pointing from the burial location to the point of maximum genetic similarity). Each dot represents the mean vector for one sample across 25 temporal resampling runs that reflect the age uncertainties of the input samples. Each observation comes with error bars on the x- and y-axis. On the x-axis, these cover the limits of the 2-sigma range of the age probability distribution; on the y-axis, they show one standard deviation of the age resampling distribution. The smooth gray curve printed below the samples is a 400-y moving mean for the spatial distance. It is calculated from the total set of the resampled iterations. The dark gray ribbon accommodating this mean curve is two times the standard error of the mean based on the mean dots displayed here. It is visualized as infinite if a given 400-y time window has less than two samples. The barcode plot at the bottom of each subplot documents the diachronic data coverage for each region. Only samples with a median age between 7300BC and 1500AD were considered for this analysis. *SI Appendix*, Text 3.3 for a more detailed description of the algorithm behind this figure. Alternative versions are provided in *SI Appendix*, Figs. S10-S13.

The results of this large-scale application are compiled in Fig. 5, both with the lengths of individual mobility vectors (shown on the y-axis) and their direction (shown in color according to the legend). While, in principle, we can apply our algorithm to every sample in the dataset, we here focus on a selection of confined regions with acceptable coverage of samples throughout the study time period (Fig. 1; *SI Appendix*, Fig. S10 and Text 5 feature two more regions, Southeastern Europe and the Western Pontic steppe). We mostly consider patterns emerging from long-distance signals, observed as individuals with large mobility-distances (around 1,000 km and further), as these tend to correspond to events described previously in the literature and thereby provide a proof-of-concept for our method. However, beyond these long-distance signals, we highlight a considerable level of complexity of smaller-scale signals that may harbor information yet to be explored. Shown along the individual distances is a moving average curve together with an error band (in gray shading), which may help putting the largest individual-based events into perspective. Alternative visualizations of the time series shown in Fig. 5 are available with *SI Appendix*, Figs. S14, S15 and S16, which show these data as a sequence of windrose plots, relative distance fractions, and a map series with all individual vectors.

One core parameter for this analysis is the retrospection distance already introduced for the individual examples. Informed by the temporal lengthscale parameter of the GPR kernel function, we decided to set it to 667 y for the whole dataset but also explored lower and higher values (*SI Appendix*, Text. 5 and Figs. S12 and S13). We also ran the entire analysis for the Projection PCA setup with 5 output dimensions (PCA5) mentioned above (*SI Appendix*, Fig. S11).

Beginning with our time series from Great Britain and Ireland, the largest observed individual signals correspond to the Early Neolithic in the 4th millennium BC (37–41), the Bell-Beaker transition after around 2500BC (42, 43), Roman Britain, and the Viking period (44). Note, for example, the indicative individuals I2657, I5367 (43), 3DRIF-26 (> 3,000 km; already discussed above) (33), and VK546 (44), each of which represent extremely long distance mobility. Direction-wise, the respective mobility peaks are consistent with what we know about the sources for these events, with Southern sources during the Neolithic, and Eastern sources during the Bell-Beaker and Viking periods. The Neolithic transition is visible in our mobility proxy as a clear upward jump, both in the average and individual mobility distances, contrasting the few sufficiently well-covered and apparently “local” Mesolithic individuals from before 4000BC. But this peak only dies down surprisingly slowly until the first half of the third millennium, attributing almost every Neolithic individual a foreign ancestral origin. To some degree, this might be an effect of the smooth, only slowly recoiling ancestry interpolation model and the peripheral position of Britain and Ireland, which renders locations on the continent disproportionately likely. But the tardiness of the recovery also supports the assumption of a large and stable sphere of interaction or at least strong genetic similarity across Western Europe during the Neolithic. We will discuss a corresponding observation below for Iberia. The following Bronze Age peak, triggered by incoming ancestry ultimately from Eastern Europe, is remarkably strong and persists even after the initial Bell-Beaker transition. For the period after 2000 BC, Olalde et al. suggest a much more homogeneous gene pool, which does not rule out the possibility of incoming continental populations with higher proportions of Neolithic-related ancestry (43), though. These might be one reason for the mobility vectors pointing to the East and South in the Middle Bronze Age, also consistent with the narrative recently established by Patterson et al. (45).

For Central Europe, we observe similar peaks as for Britain and Ireland. The Neolithic expansion reaches this area in the late sixth millennium and leads to a first, strong uptick of the mobility signal from the Southeast (46–49), visible, for example, in the aforementioned Stuttgart individual (25). These Neolithic individuals’ maximum similarity points cover a wide corridor from western Anatolia to the Balkans, with some directed also toward the southern route of the Neolithic in Italy. The mobility pulse then dies down in the fifth millennium. An interesting case in this spatiotemporal context is individual N22 from modern-day Poland. Fernandes et al. (50) describe them as “the most recent individual (≈4300 BCE) with a complete genomic WHG attribution to be found to date in an area occupied by Danubian Neolithic farmers,” which causes the similarity search to link them to the remaining hunter-gatherer populations in the Baltic region, Ireland and elsewhere. In the first half of the third millennium, Steppe ancestry arrives, as observed in many Late Neolithic Corded Ware individuals—like aforementioned RISE434 (29). Their mobility vectors then clearly point into the far East and Northeast (9, 29, 49, 51, 52). This strong signal is heterogeneous both in distance and directionality. We caution that the spread of Steppe ancestry did most likely not follow a perfect wave-of-advance-like pattern, leaving pockets of unaffected or only later-affected ancestry behind, which will inevitably result in more erratic mobility estimates. After 1500BC, the data density for Central Europe decreases, and general observations become more difficult. Given archaeological and eventually historical evidence, it is not unreasonable to assume a high degree of mobility in the Bronze Age and later, the Iron Age and the Medieval period, connecting Central Europe to France, Great Britain, Southern Scandinavia, Eastern Europe, and the Balkans, catalyzed by different cultural processes (53). Two remarkable individuals with long mobility vectors are WEZ35, which is representative of the relatively unstructured population documented from the Tollense Bronze Age battlefield in northern Germany (54) and AED1108 from Bavaria with strong skull deformation and about 20% East Asian ancestry (55).

Already the first hunter-gatherer individual available from Iberia—Chan (56)—has a very large mean mobility vector pointing to the Iron Gates on the Balkans. This signal is not reliable, though, given the fact that no local, preceding reference data exist, which could inform the ancestry field for the similarity search to appreciable accuracy. Much more relevant are the observations for Early Neolithic individuals like CB13 (57). They document the southern route of the Neolithic expansion (58). From the end of the fifth millennium to the middle of the third millennium, many individuals from Iberia are attributed long mobility vectors toward the North and Northeast, e.g., LugarCanto45 (59), although others have described this period as a time of relative genetic stability (36). This forms a parallel observation to South- and West-facing mobility vectors described above for Great Britain, Ireland, and Central Europe between 4000 and 2500BC. We suspect that this crisscrossing of vectors may be caused by the low levels of genetic differentiation among different Neolithic populations. The Neolithic expansion and the following resurgence of hunter-gatherer ancestry in populations in Iberia, Central Europe, and Great Britain might have created a large geographic area of very similar genetic ancestry (Fig. 2). Alternatively—or additionally—the Atlantic sphere of influence connecting Western European megalithic cultures (to be taken up later in the Bell Beaker phenomenon and beyond) could have induced a high degree of mobility in said region (38, 60, 61). More clearly interpretable signals emerge later in the third millennium in Iberia with the arrival of Steppe ancestry—well visible through mobility vectors pointing to the far Northeast for individuals like I3239 (36). In the Iron Age and later, we observe some nonlocality from the North, e.g., I8341 (36)—which could potentially be connected to the spread of Central European, Celtic ancestry and languages to the region (62)—and from the East, e.g. I8215 (36), possibly through Greek and Roman influence. We note that the relative lack of samples from Northern Africa masks potential mobility that might have taken place between Europe and Africa (63).

The final focal region studied here, Italy, comprises not only the Italian Peninsula but also Sicily and Sardinia. These go through partially independent developments not comprehensively represented in the available data. Samples from the sixth millennium are limited to Sicily as well as Northern and Central Italy. They fit well with what we know about the southern route of the Neolithic Expansion with ancestry arriving from the East (34). Indeed, the ancestry vectors of Early Neolithic samples like R2 (34) point directly to western Anatolia. A few 100 y later, the Neolithic ancestry profile is distributed across large parts of Europe, and our derived mobility proxy reflects less a point of origin for the respective Neolithic samples, but rather their entanglement in the preceding cross-European mobility phenomenon. We assume this to be the reason for the moderately strong mobility signal we measure from the fifth to the middle of the fourth millennium, e.g., LON003 (64), arising despite almost all our input data are from Sardinia, where others have observed genetic continuity until the first millennium BC (64). In the third millennium, Steppe ancestry arrived on the Italian peninsula, heralding multiple long-distance mobility signals: The affected Sicilian and mainland individuals show affinity to the North and East—most notably I11443 (65) from Sicily, which was reported to have the highest amount of Steppe ancestry in ref. 65. Note the Chalcolithic sample I15940 (65) from Sardinia with their eastern mobility signal. Fernandes et al. (2020) identified them as an outlier with “significant affinity to Levantine and North African Neolithic individuals.” The second millennium in our Italy time series is almost exclusively covered by samples from Sardinia and Sicily, with a low mobility proxy signaling genetic isolation. During the Iron Age, Sardinia and the Italian mainland become once more part of an exuberant Mediterranean mobility network, as shown, for example, by VIL011 from a Carthaginian/Phoenician–Punic context (64) or R850 (34), which Antonio et al. could model as a “mixture between local people and an ancient Near Eastern population [...].” We finally observe the most extreme signals of nonlocality in Italy during the height of the Roman empire, in the first centuries AD, where a unique pattern of East–West mobility emerges, consistent with a strong Near Eastern influx into the city of Rome, visible, e.g., with individuals like R67 (34).

## Discussion

Our method to estimate human mobility from genetic data is based on a simple key principle: Changes in genetic profiles are informative about population movements. This key principle is not new but, in fact, the core assumption behind archaeogenetic studies reconstructing mobility and migration in Western Eurasia, most notably movements associated with the Neolithic expansion (e.g., refs. 26, 27, 39, 47, 48, 50, 57, 66 and 67) and the arrival of Steppe ancestry (e.g., refs. 9, 29, 42, 43, 51, 52, 65 and 68). In our algorithm, we have used this basic principle to derive mobility at an individual level, by interpreting human genetic profiles as quantitative proxies for a biogeographic field. This perspective unlocks a spatiotemporal, probabilistic similarity search for genetic ancestry, which we consider a valuable tool to understand an individual’s mobility history. It also forms the basis to quantify individual-wise mobility on a large scale to derive diachronic, regional summary statistics.

A first conceptual challenge for this methodology emerges from the fact that the genetic–spatial mapping changes through time, due to human movement, the very subject of this study. There can never be a perfect representation of a biogeographic ancestry field since genetic ancestry is not tied to geographic space but to the highly mobile individuals living within it. In our method, we have tried to approximate past ancestry using Gaussian process regression, which erects a slowly changing field, effectively smoothing out the rapid changes brought about by individual humans’ agency. Beyond said conceptual issue, this directly links to the practical concern of severe sparsity in the archaeogenetic record informing the interpolated field. Thanks to the fully probabilistic nature of our ancestry similarity search, missingness-induced uncertainty is handed down from the interpolation to the similarity probability surfaces. They will generally show lower values for badly covered areas. This empowers the algorithm to be used with highly unevenly sampled datasets, which is inevitably the case for the human archaeogenetic record.

Of course, though, this does not solve cases of entirely missing ancestry profiles (e.g., Northern Africa), which simply can not be accurately represented and considered, as long as key samples are nonexistent. It also does not accurately capture situations of multiple coexisting ancestries living in close proximity in space and time. Interpolation will in such cases create an average ancestry profile which may not be meaningful and cause the incorrect assignment of similarity probabilities. We have shown the example of individual N22, who carries a genetic hunter-gatherer profile at a point in space and time, when other individuals in its vicinity feature an Anatolian farmer profile (A similar case is individual I2534, *SI Appendix*, Text 5). In this case, we can assume a cultural admixture barrier, which the interpolation cannot correctly resolve.

Despite these limitations and the possibility that the condensing of sample-wise similarity probability surfaces into simple vectors could amplify the effect of inconclusive spatial assignment, our large-scale mobility estimation results generally fit the published state of research. The Neolithic demographic expansion, the Steppe migration, and a number of smaller ancestry relocation events show clearly visible signals for most of our study regions. The simulation experiment in *SI Appendix*, Text 4 also gives us some confidence that the similarity search algorithm is capable of producing accurate results even for small genetic differences as long as these differences maintain spatial stability for a sufficient amount of time. This holds true even for donor and receptor populations in close spatial proximity.

We therefore expect the mobility estimation to perform well in picking up outlier individuals who moved over a long distance in a short amount of time. We are reasonably certain that major turnover events with significant shifts in the MDS or PCA for a given point in space will be reliably detected. But the smaller the scope of a mobility event and the longer the duration of the process, the more diffuse and unclear the respective signal gets. We also observed a center–periphery effect in the mobility curves, with generally higher values for geographically peripheral regions like Britain and Ireland, Iberia or Italy, compared to more central ones like Central or Southeastern Europe. While this might be a real signal to some degree, it may also be an outcome of imprecise assignments of maximum similarity points, which happen to point to the geographic center rather than the outskirts.

To improve the results obtained in this paper, several important directions may be taken. Future research will probably be in a position to include more data as the sampling gaps in available ancient DNA data are quickly filled. This will make large-scale meta-analysis more and more feasible and will allow for increased postdiction model resolution. Beyond that, developing more sophisticated spatiotemporal interpolation models will be a core challenge. We are convinced that Gaussian process regression is a very powerful method, but other approaches may allow for more heterogeneous covariance settings dependent on the data density in space and time or even involve full-scale machine-learning (69). Concerning the derived mobility estimation method (consisting in our case of maximum-likelihood mobility vectors), entirely different algorithms may be conceived to get a more robust and precise measure compared to the one we present here. It may also be possible to quantify nongenomic information and assign priors from artifact refitting (70), isotope analysis (71), or least-cost-path analysis (72). We see great potential in codifying linguistic, historical, or archaeological data to derive alternative, large-scale measures of human mobility (73, 74). Shifts in local ancestry can, after all, not only be the outcome of the often-cited deliberate “mass migration” but potentially also of bottlenecks (75), forced migration (76), or sociocultural phenomena, which require combining interdisciplinary lines of evidence.

## Materials and Methods

### Dataset.

*SI Appendix*, Datasets S1, S2, and S3 summarize the input and output dataset for this paper, including the mean similarity search output statistics. *SI Appendix*, *Meta Information for the Datasets S1, S2, and S3* for a description of the meaning of each variable/column. The raw input data were compiled from the Allen Ancient DNA Resource (AADR) V50.0 (released on 2021-10-10) (22) and modified with convertf (77) and software tools from the genotype data management system Poseidon (https://github.com/poseidon-framework). *SI Appendix*, *Bibliography: AADR Dataset* for a list of papers providing the individual samples. We included only ancient DNA samples and removed samples without spatial or temporal position information as well as samples outside of the defined research area (Fig. 1) and time window (median age within 8000calBC–2000calAD).

The dataset includes both samples whose DNA libraries have undergone in-solution enrichment capture as well as samples who have been sequenced evenly across the entire genome using the so-called shotgun approach. Each sample covers an individual subset of the 1240K SNP array (23). For quality filtering, we kept only samples with 25,000 or more recovered autosomal SNPs on this array, determinable molecular sex and—for male individuals—an X-chromosome contamination value determined with ANGSD (78) < 0.1. We also excluded samples that were explicitly marked as contaminated by the respective authors or assessed negatively in the AADR. In a final data-filtering step, we calculated pairwise distances (1 - proportion of alleles identical by state) among all samples and kept only the best preserved one from pairs/groups with distance values < 0.245, to remove closely related individuals or samples from the same individual. *SI Appendix*, Text 1 for more details on the SNP selection process.

All radiocarbon dates in the archaeological context data were recalibrated with the R package Bchron (79) (intercept calibration with IntCal20). Multiple radiocarbon dates for one sample were merged with sum calibration.

### Multidimensional Scaling.

Multidimensional scaling is a dimensionality reduction method that can be applied to genetic data to derive positions in a genetic–distance space for individual samples. Before running it on our dataset with plink ––mdsplot v.1.9 (80), we removed SNPs in previously identified regions of high linkage disequilibrium within the 1240K SNP panel range according to Price et al. and Anderson et al. (81, 82). *SI Appendix*, Text 1 for other dimension reduction methods we explored (PCA, Projection PCA, EMU).

### Gaussian Process Regression.

Gaussian process regression is an interpolation method for n-dimensional space. The term Gaussian process means that a set of observations is modeled as the outcome of a multivariate normal distribution. The method allows making predictions for a dependent variable based on the position in independent variable space (83). It is a long-established method of geostatistics, where it is known as kriging (84). Here, we treat the position in spatial space (coordinates projected to EPSG:3035) and temporal space as three independent variables that are used to predict the dependent position on each of two (or more) MDS (or PCA) result dimensions.

A crucial step in the application of Gaussian process regression is the selection of a sensible covariance function (kernel) that effectively describes the degree and range of long-distance effect the model assumes for individual observations. We followed the default choice for an anisotropic Gaussian kernel implemented in the R package laGP v.1.5-7 (85). laGP provides comparatively fast and accurate local approximate Gaussian process modeling (86). The default laGP kernel has the form

$$\begin{array}{cc} Cov(x,x^{\prime }) & =\tau^{2}\left(\exp\left[-\sum_{k=1}^{p}\frac{{\left(x_{k}-x_{k}^{\prime }\right)}^{2}}{\theta_{k}}\right]+\eta \delta \left(x-x^{\prime }\right)\right), \end{array}$$

with (*x*_*k*_ − *x*′_*k*_) as the distance between all observations *x*, *x*′ in the different dimensions *k* and the kernel size scaling factor *θ*_*k*_ for each dimension. *η* is the so-called nugget term to account for different observations of the dependent variable at the same position in independent variable space. The values of *θ*_*k*_ (spatial and temporal) and *η* have to be fixed, which is the second important decision necessary to define the covariance matrix. We applied multiple approaches (variogram analysis, maximum likelihood estimation, cross-validation) to estimate these parameters. *SI Appendix*, Text 2 for more details.

### Similarity Search and Mobility Estimation Algorithm.

Our probabilistic similarity search algorithm determines the likelihood to observe a sample’s MDS (or PCA) coordinates at a certain point in space and time and we apply it to compute the relative spatial distribution of similarity probabilities in a given timeslice. *SI Appendix*, Text 3 for a detailed explanation. This works in principle as demonstrated by the toy simulation in *SI Appendix*, Text 4. The algorithm for the large-scale mobility estimation constructed from individual-wise similarity searches is explained in detail in *SI Appendix*, Text 3.3. The effect of alternative multivariate dimension reduction methods and different settings for the retrospection distance on the mobility estimation is shown in *SI Appendix*, Text 5.

## Supplementary Material

Appendix 01 (PDF)

## Data Availability

The code for this paper is available in a repository here: http://dx.doi.org/10.17605/OSF.IO/6UWM5. From that, we outsourced the main similarity search and mobility estimation workflow into an R package available here: https://github.com/nevrome/mobest. All data analysis and plotting was done in R (87) with the following packages: checkmate (88), cowplot (89), fractional (90), future (91), ggh4x (92), ggnewscale (93), ggpubr (94), ggrepel (95), ggridges (96), igraph (97), khroma (98), latex2exp (99), lemon (100), progress (101), rnaturalearth (102), sf (103), smartsnp (104), viridis (105), and, finally, the tidyverse and the many packages within it ref. 106. Previously published data were used for this work (Allen Ancient DNA Resource https://reich.hms.harvard.edu/allen-ancient-dna-resource-aadr-downloadable-genotypes-present-day-and-ancient-dna-data, version 50.0).
