# Supplementary material for: Estimating human mobility in Holocene Western Eurasia with large-scale ancient genomic data
Source: Proc Natl Acad Sci U S A. 2023 Feb 23;120(9):e2218375120. doi: 10.1073/pnas.2218375120 (PMC9992830; doi:10.1073/pnas.2218375120)
Supplement: Supplementary file 1 — Appendix 01 (PDF) [file pnas.2218375120.sapp.pdf]

# Supplementary Material

for

*Estimating human mobility in Holocene Western Eurasia with large-scale ancient genomic data*

Clemens Schmid & Stephan Schiffels

2023

# Contents


**A Supplementary Figures** **1**

**B Meta information for the Datasets S1, S2 and S3** **18**

**1 Supplementary Text: Creating a simplified genetic space** **20**
1.1 Finding the most suitable multivariate analysis method . . . 20

**2 Supplementary Text: Interpolation parameter estimation** **25**
2.1 Variogram analysis . . . 26
2.2 Maximum likelihood estimation . . . 29
2.3 Crossvalidation . . . 32

**3 Supplementary Text: The similarity search algorithm** **35**
3.1 Ancestry and sample-wise similarity fields . . . 35
3.2 Diachronic mobility proxy . . . 37
3.3 Concrete steps for the mobility estimation . . . 37

**4 Supplementary Text: A toy simulation to demonstrate the similarity search algorithm** **39**
4.1 Simulation setup . . . 40
4.2 Interpolation . . . 42
4.3 Similarity search . . . 42
4.4 Conclusion . . . 44

**5 Supplementary Text: Mobility curve exploration** **46**
5.1 Two additional regions: Southeastern Europe and Western Pontic Steppe . . . 46
5.2 Comparing different mobility curves . . . 47

**C Bibliography: Supplementary Texts** **50**

**D Bibliography: AADR Dataset** **52**


# A Supplementary Figures

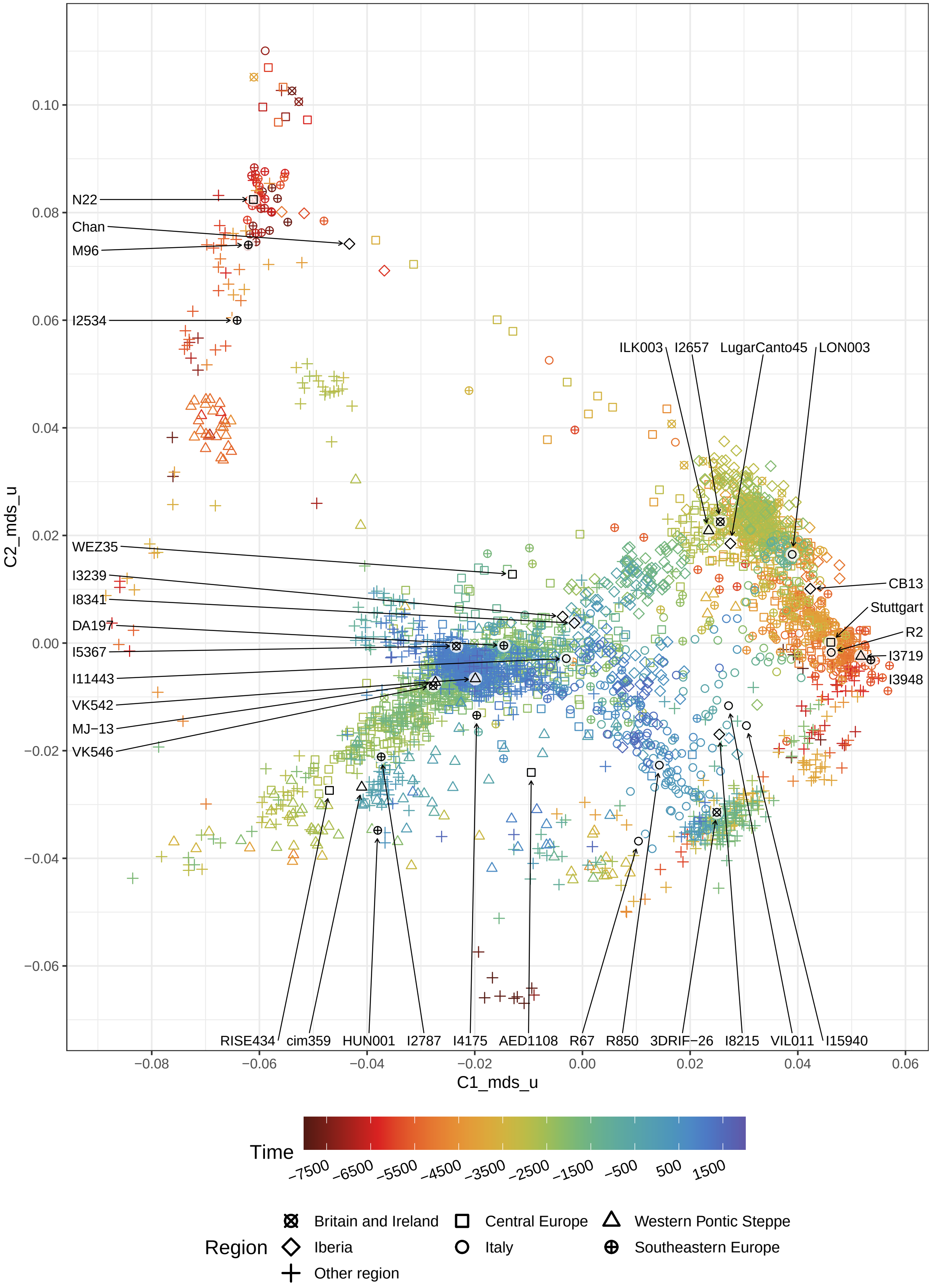


**Figure S1**: A more detailed version of Figure 2, where the individuals mentioned in the text are highlighted.

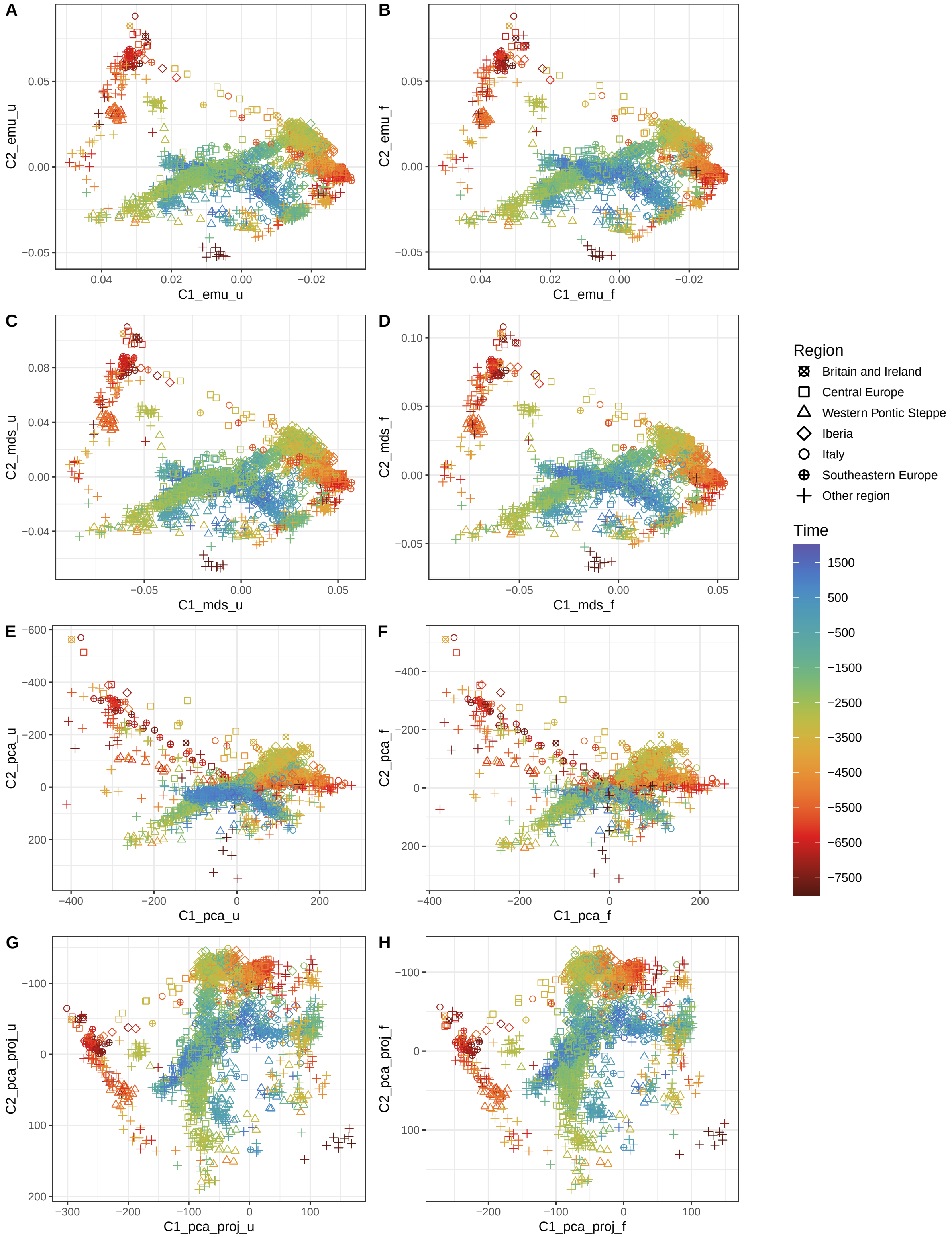


**Figure S2**: Scatter plots of samples on the first two result dimensions of the four multivariate analysis methods run for this paper (rows of the plot matrix), each in two iterations for the two tested SNP sets (columns of the plot matrix). Shape and colour according to Figure 1.

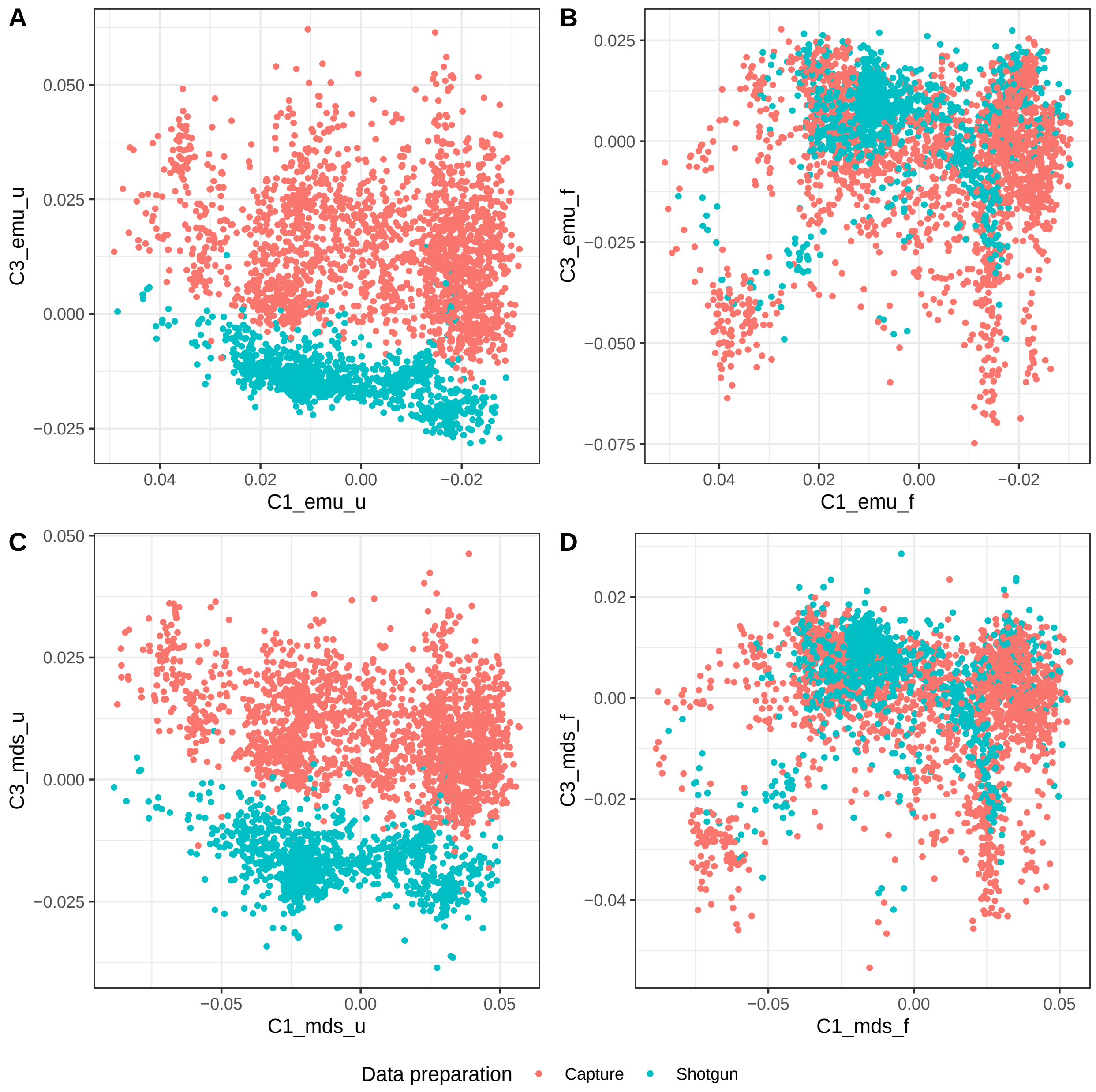


**Figure S3**: Scatterplots of samples on output dimension one and three for EMU and MDS. Both in two iterations for the two tested SNP sets: **A** and **C** with the unfiltered set, **B** and **D** for the filtered one. Samples handled with capture and shotgun technique are distinguished via dot colour.

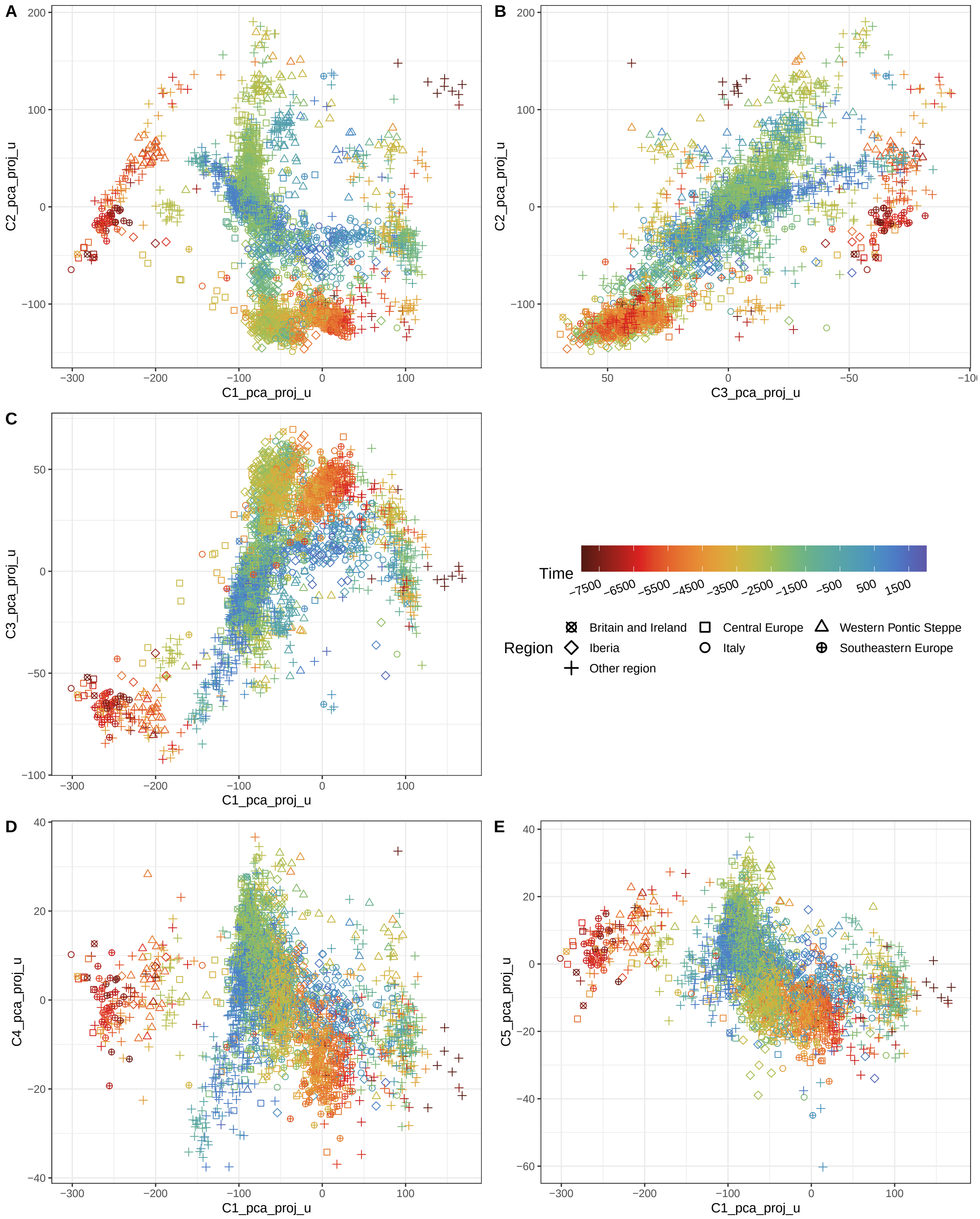


**Figure S4**: Scatter plots with the (ancient) sample distribution on the first five output dimensions of projected PCA. The modern reference samples used for the projection are left out for the sake of visual clarity. For **A**, **B** and **C**: To stay true to a 3D perspective, the printing order of each sample dot is according to the third dimension (the one not on the two axis) – with lower values always printed first. For **A** that means for example that the dots are printed in the order of their coordinate value on C3: Samples with lower values on C3 are printed first, so they are below samples with higher C3 values. **D** and **E** are ordered by C1.

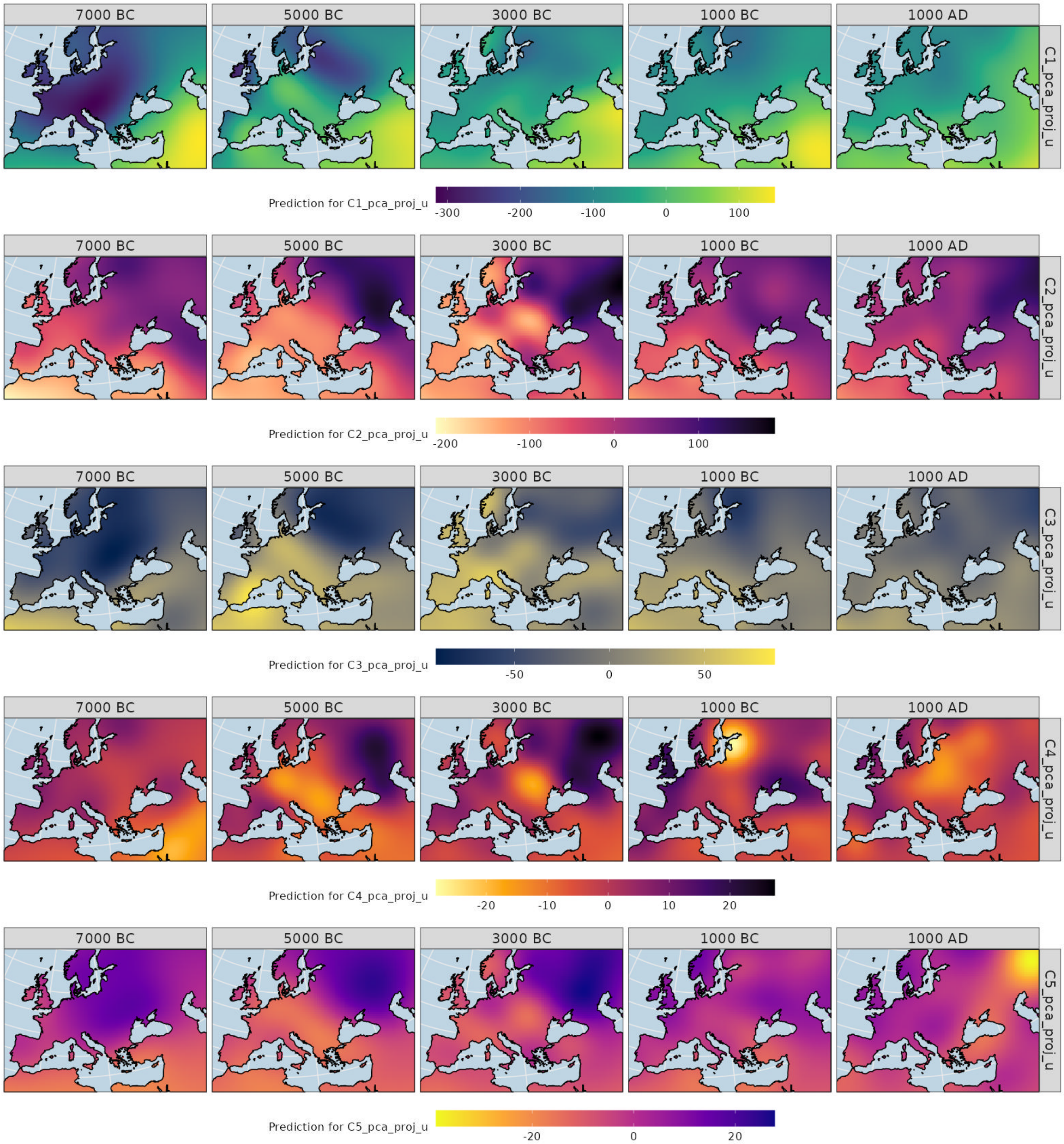


**Figure S5**: Diachronic Gaussian process regression interpolation map matrix as in Figure 3, but here for the first five output dimensions of the projected PCA. Compare Figure S4.

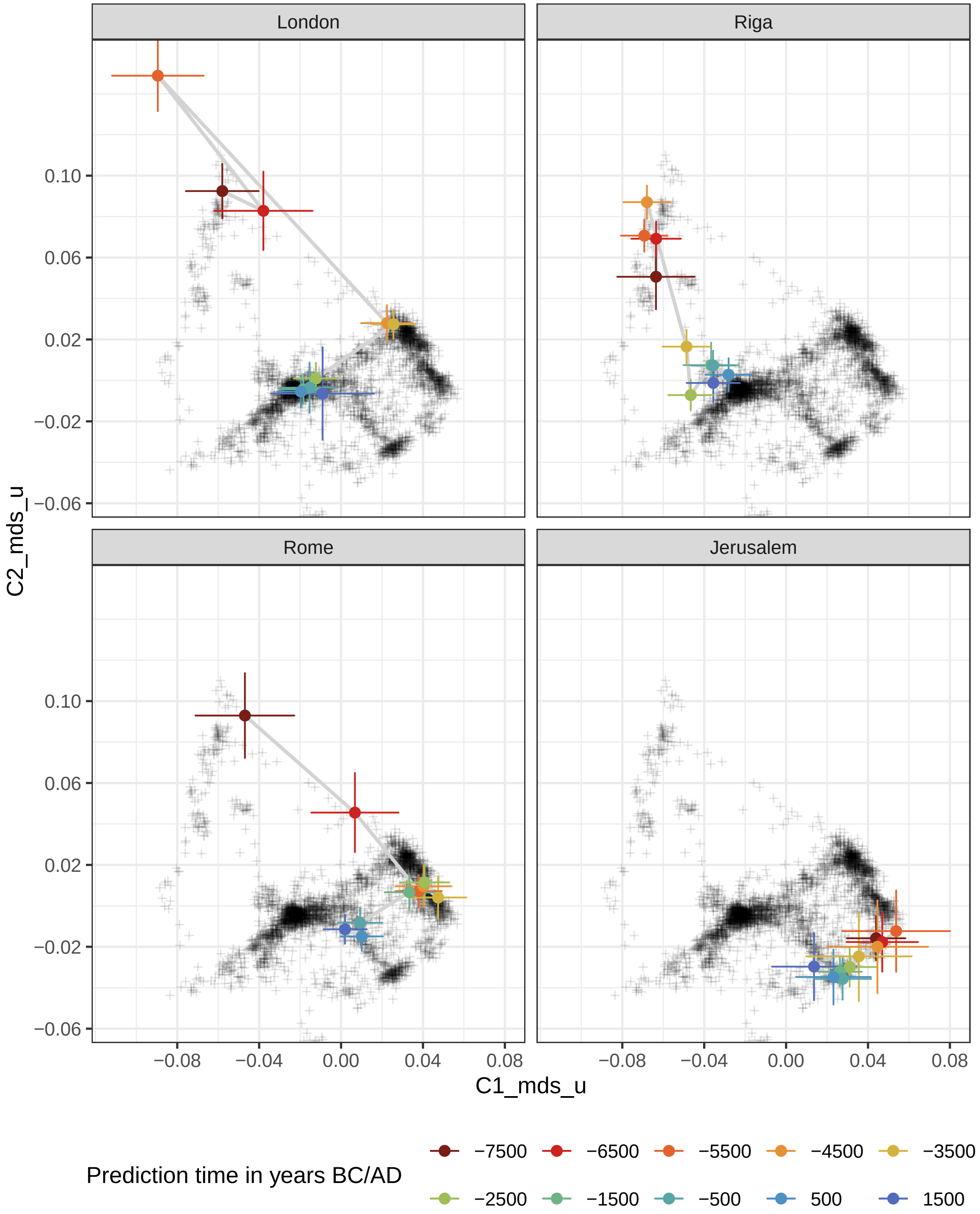


**Figure S6**: Interpolation-based reconstruction of the past ancestry development at the spatial position of modern day city centres. Each "time-path" in MDS space (see Figure 3) connects the interpolated positions in steps of 1000 years. The individual steps are colour-coded by age and horizontal and vertical error bars indicate the standard deviations given by the GPR model for this position. The black, semitransparent crosses in the background are the ancient samples as in Figure 2.

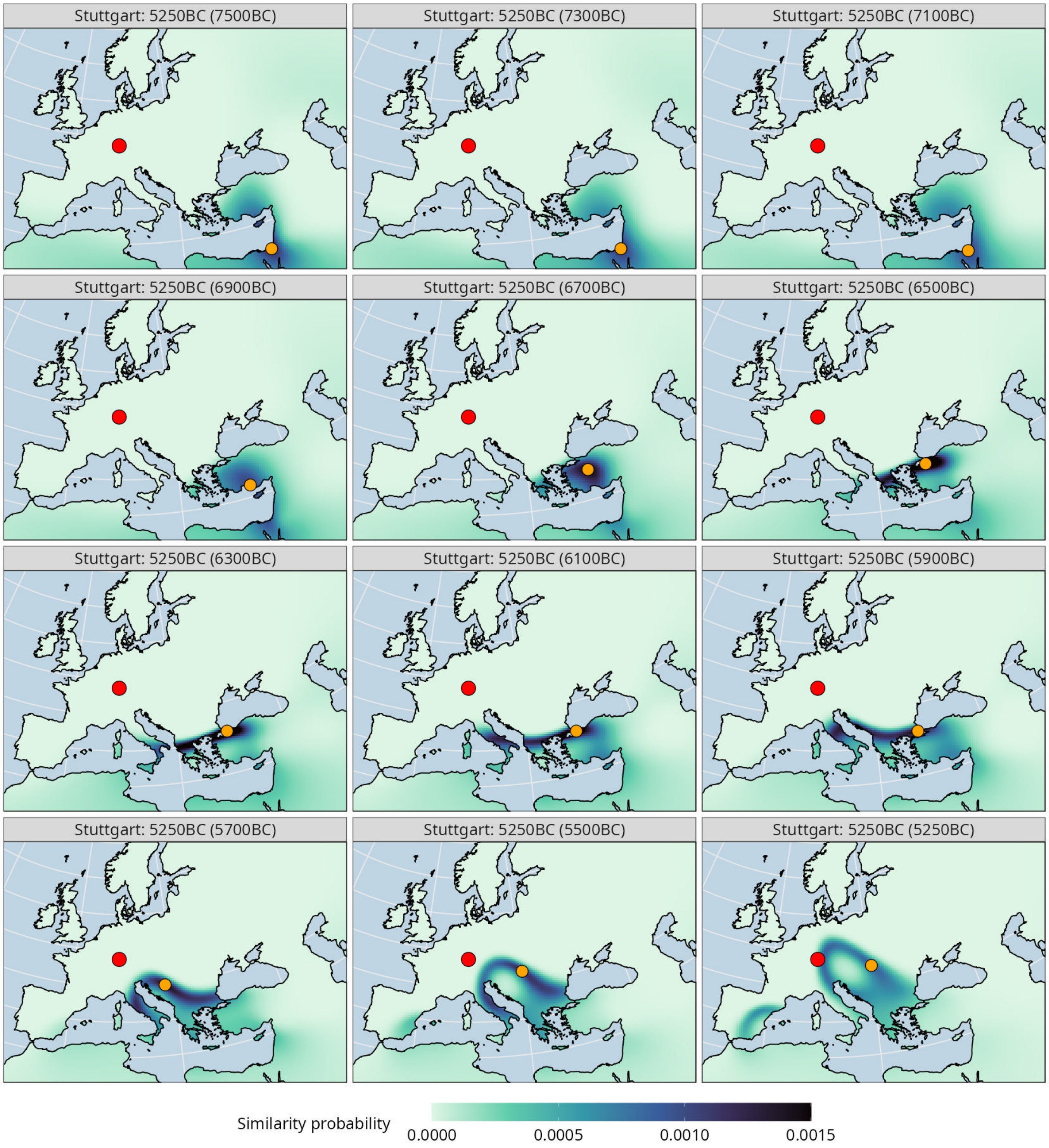


**Figure S7**: Plot matrix similar to Figure 4, but here just the Stuttgart sample with different retrospection distances through time. The absolute date of a timeslice is given in parentheses.

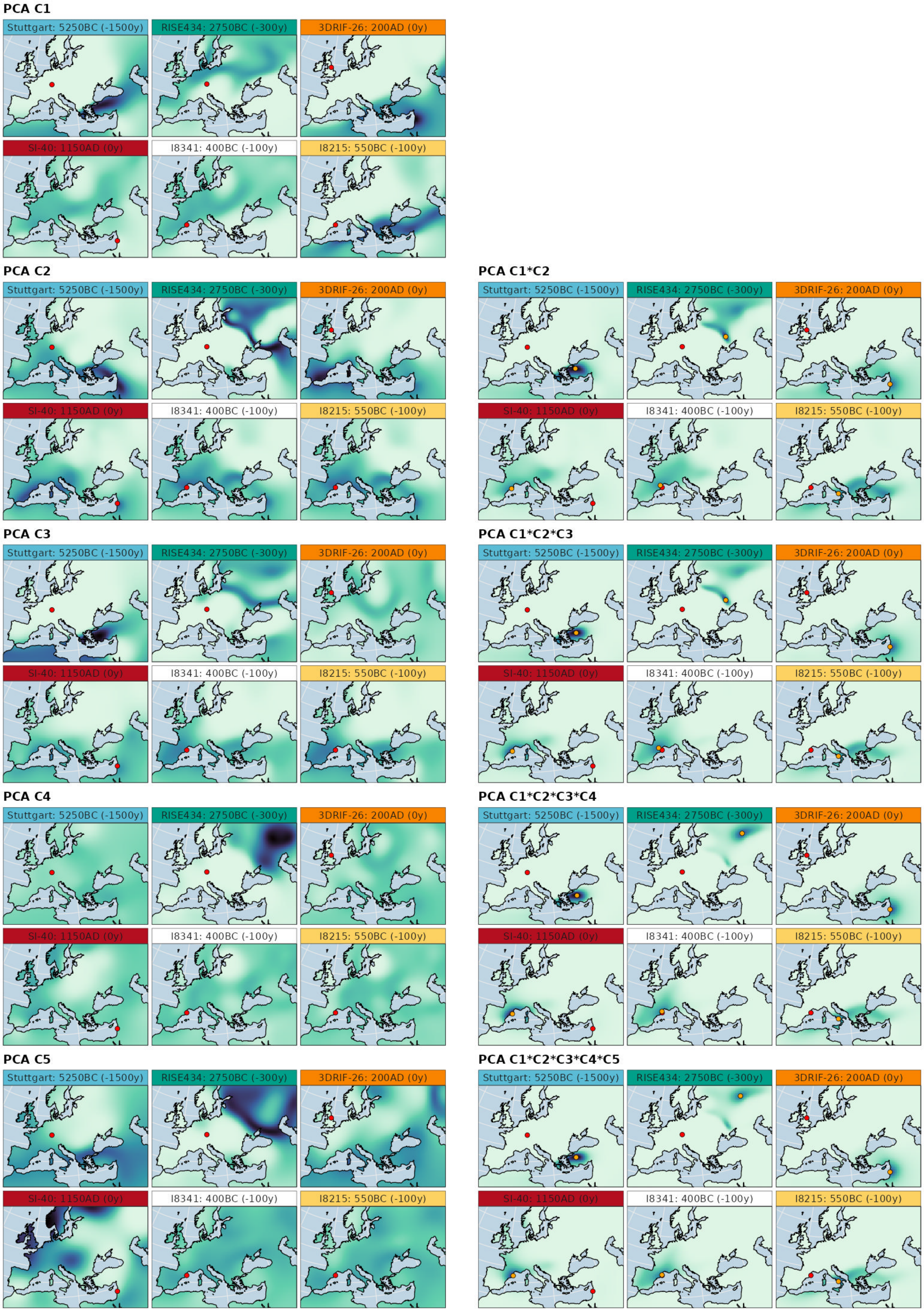




**Figure S8**: Plot matrix similar to Figure 4, but here not just for the product of two similarity search dimensions (MDS C1*C2), but for the individual projected PCA dimensions (PCA C1-C10, on the left), and their cumulative products (on the right). To simplify the comparison, colours were assigned to the facet labels. These feature the sample ID, an approximate age and the retrospection distance applied.

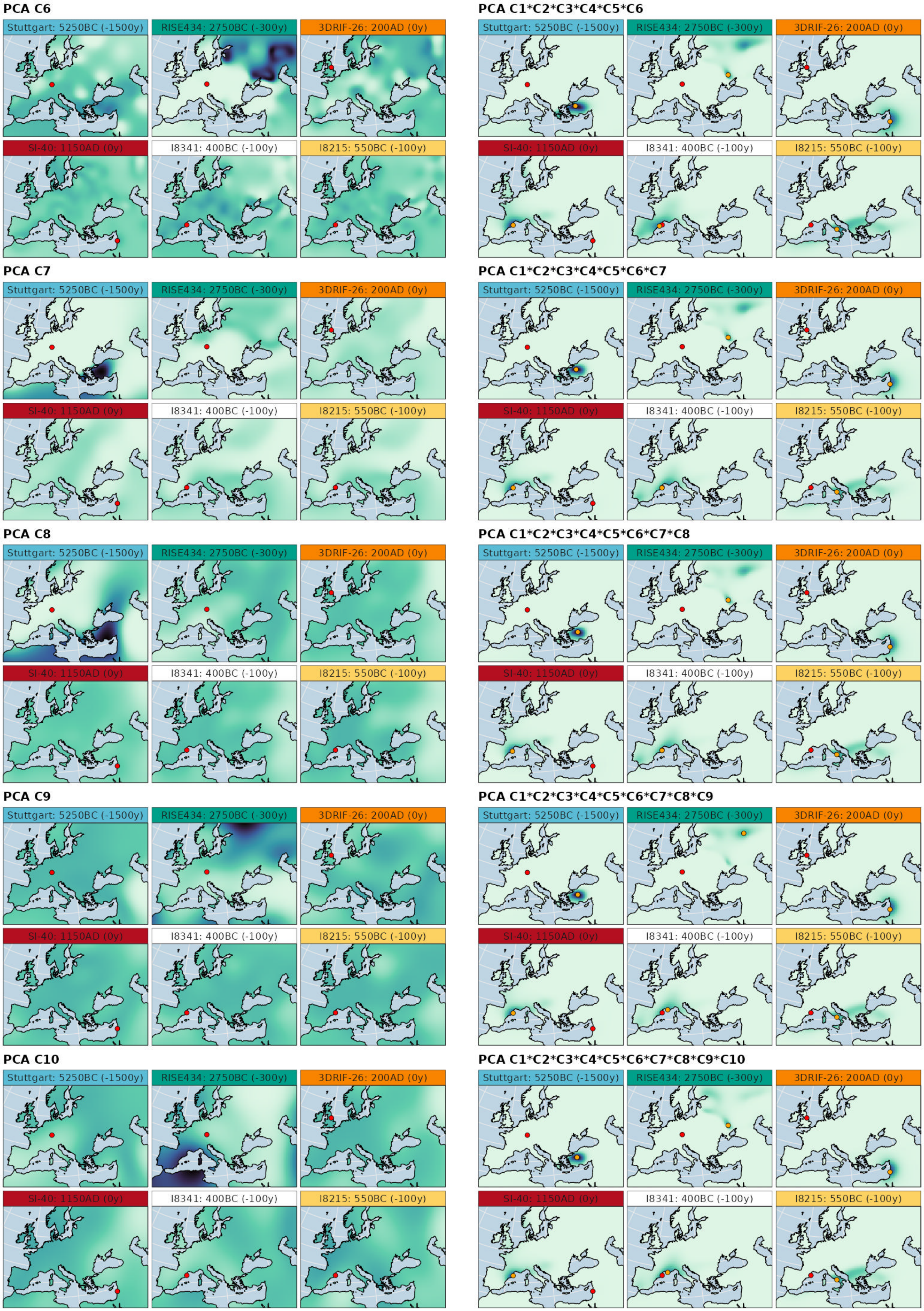




**Figure S9**: Continues Figure S8.

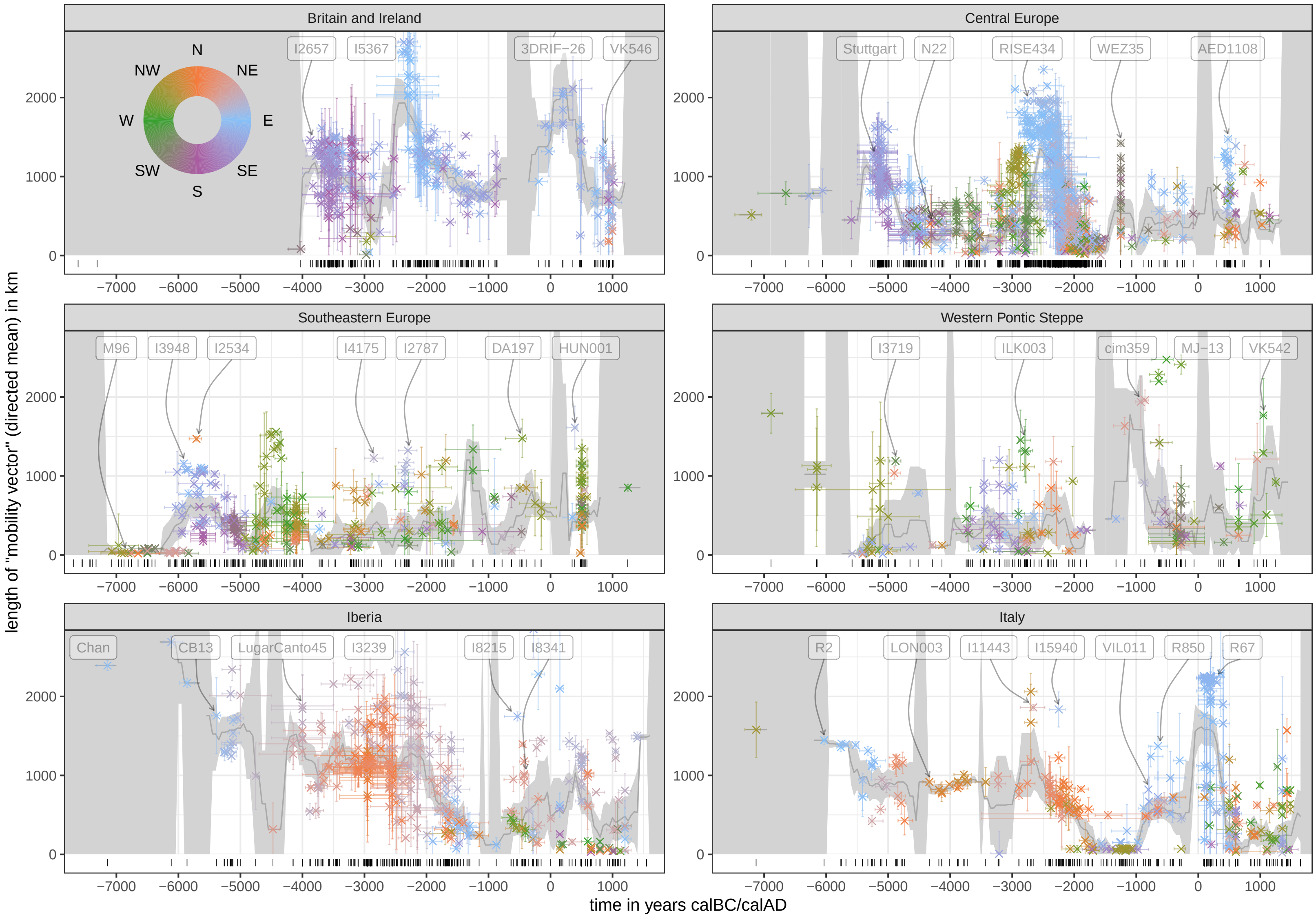


**Figure S10**: Regional mobility curves just as in Figure 5 for the mobility estimation run with **the first two MDS dimensions** (MDS2). Identical to Figure 5, but with the two additional regions *Southeastern Europe* and *Western Pontic Steppe*.

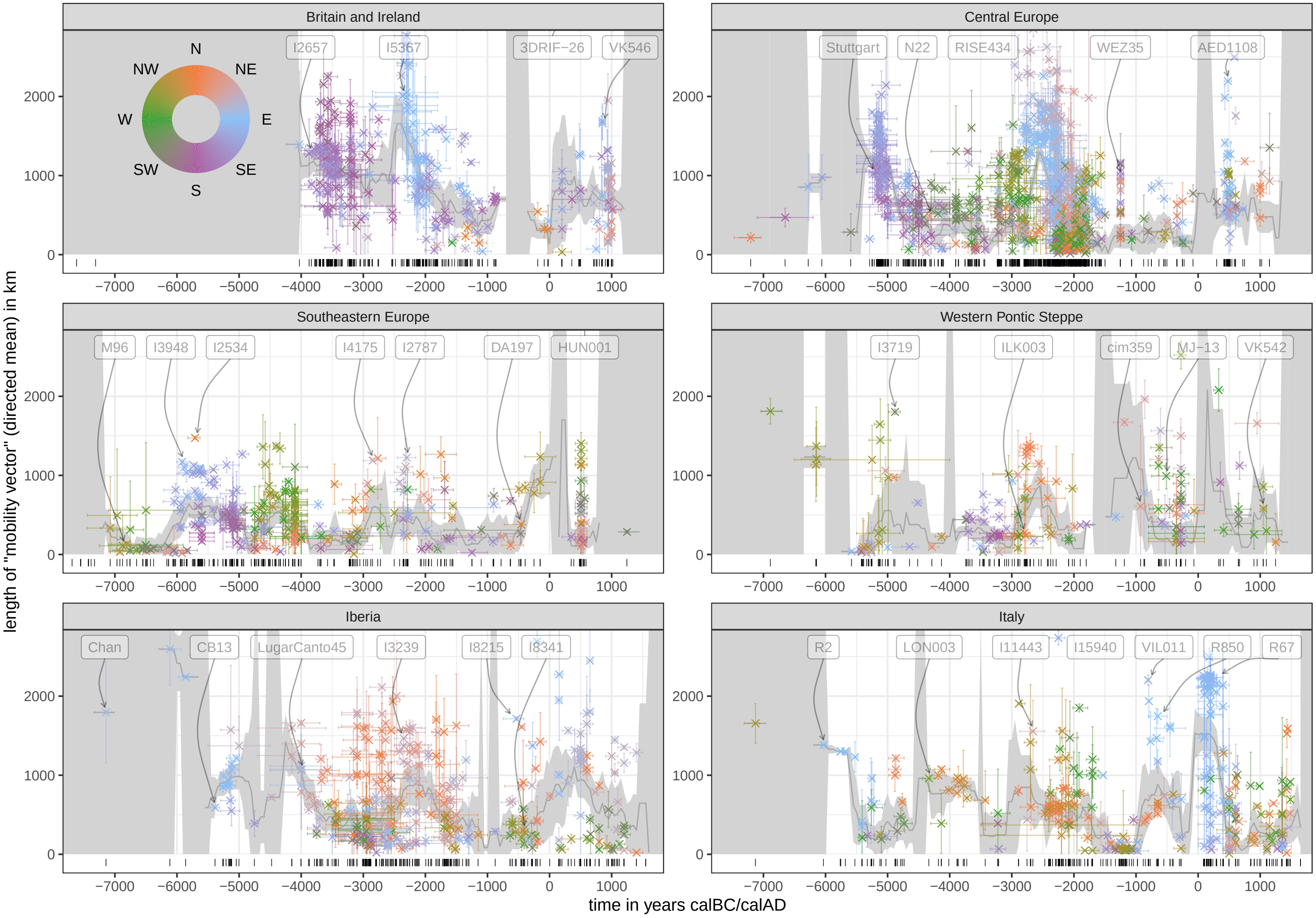


**Figure S11**: Regional mobility curves for an mobility estimation run with **the first five Projection PCA dimensions** (PCA5). Beyond that just as Figure S10. See Figure S34 for a direct comparison.

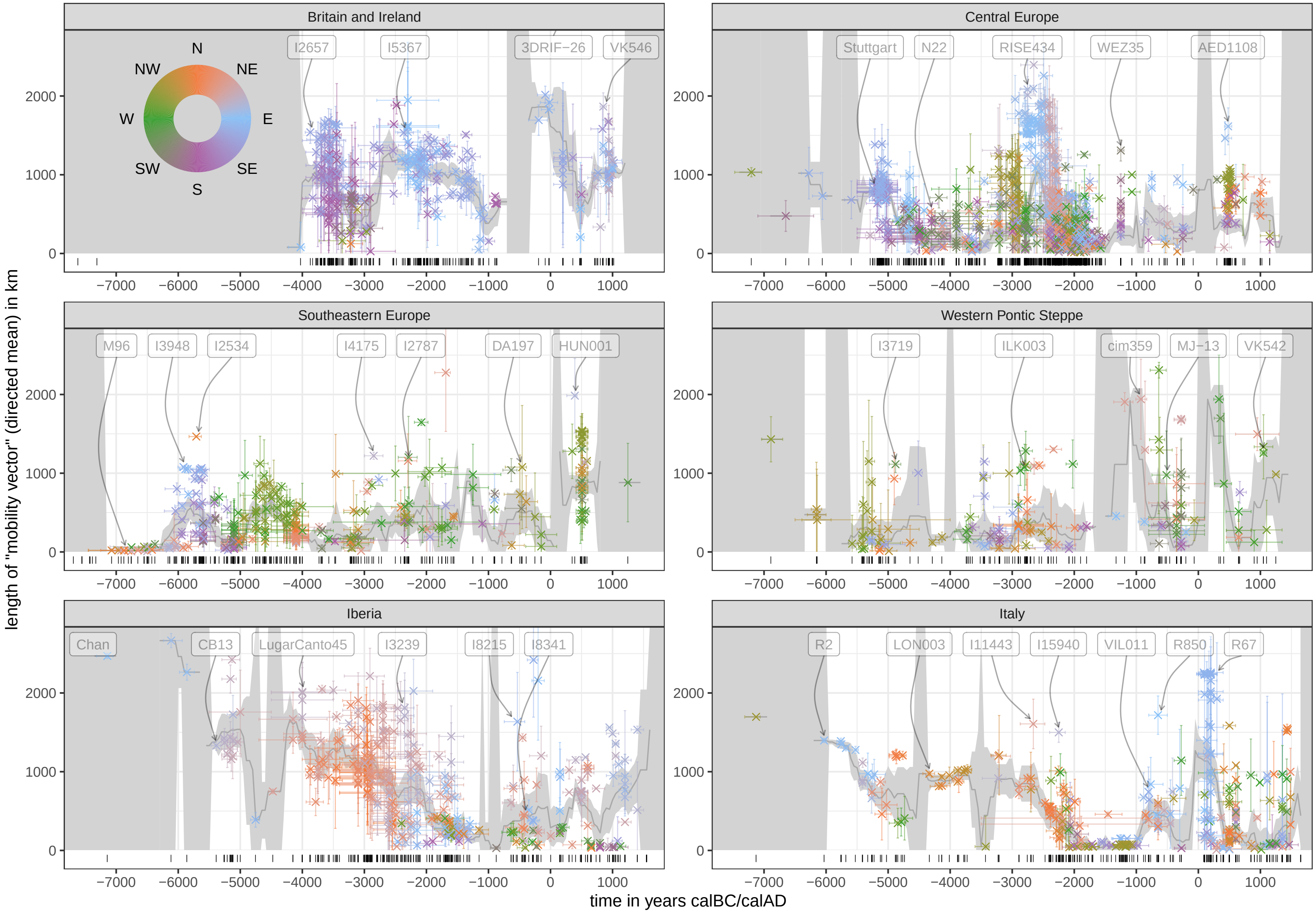


**Figure S12**: Regional mobility curves for an mobility estimation run with **the first two MDS dimensions** (MDS2) and a **lower retrospection distance**.

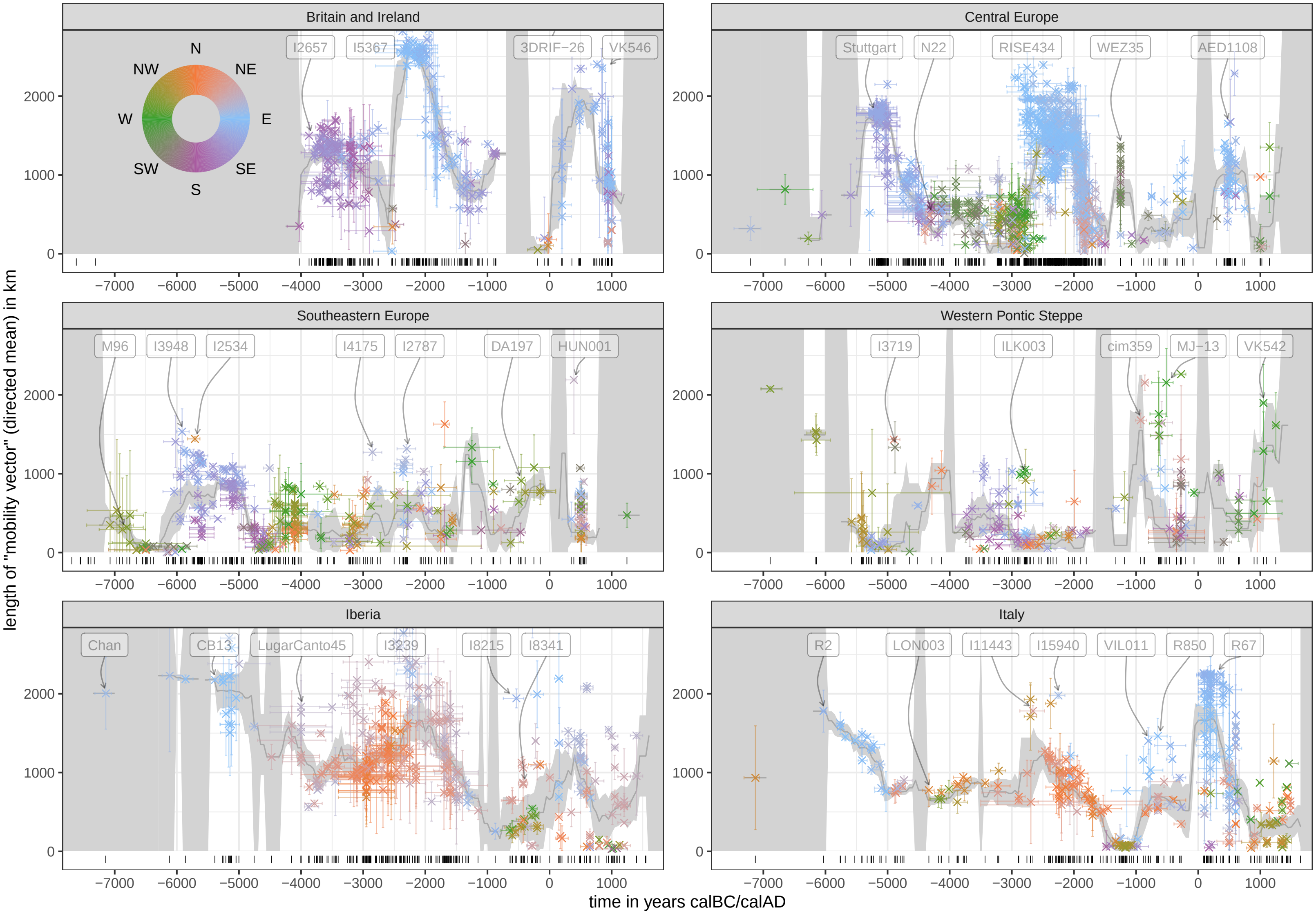


**Figure S13**: Regional mobility curves for an mobility estimation run with **the first two MDS dimensions** (MDS2) and a **higher retrospection distance**.

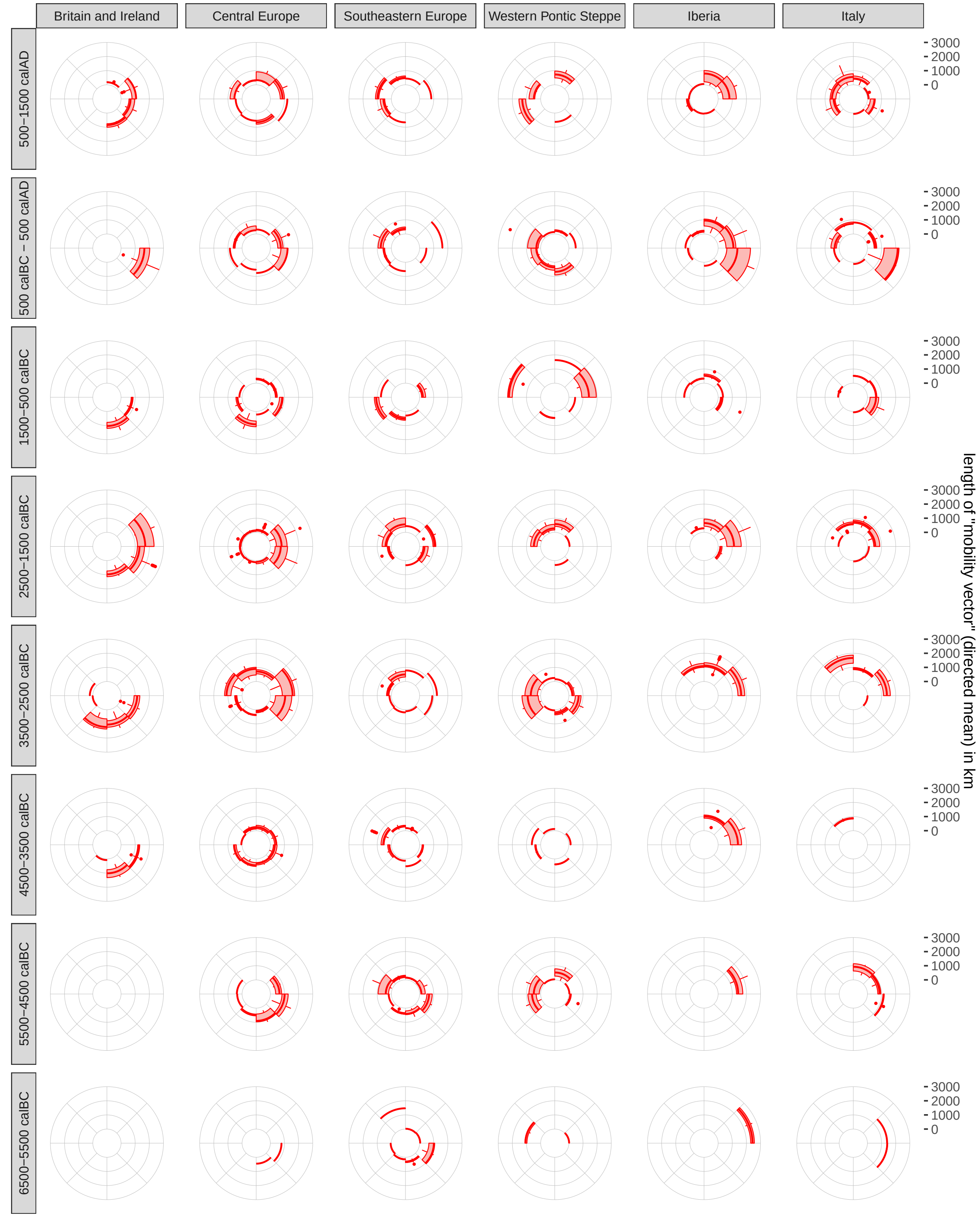


**Figure S14**: Another view on the data in Figure S10. Here the sample-wise mobility vectors are attributed to a group given by the analysis region, a 1000-year time window and the 45° angle range. Each region-time window group is represented by one plot in the plot matrix. These individual plots show the distribution of directed distances within each 45° window as a boxplot in a circular coordinate system (windrose plot). The plot matrix maps time from bottom to top and the analysis regions on the horizontal axis from left to right.

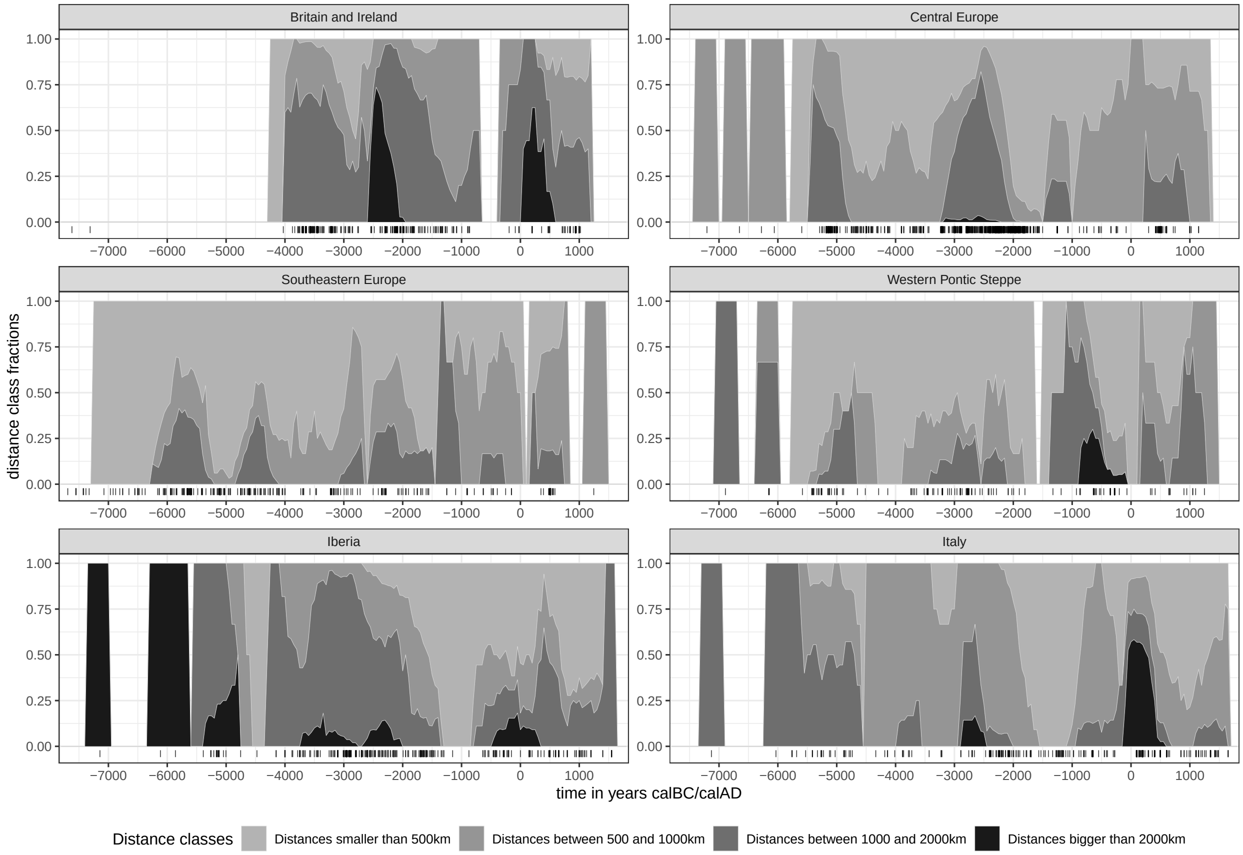


**Figure S15**: Another view on the data in Figure S10. The same sliding window used to calculate the moving mean and standard error for Figure S10 was employed here to determine proportions of distance vectors smaller, in between and bigger 500, 1000 and 2000 kilometres. These fractions are displayed as region-wise stacked area charts. No-data windows are left blank.

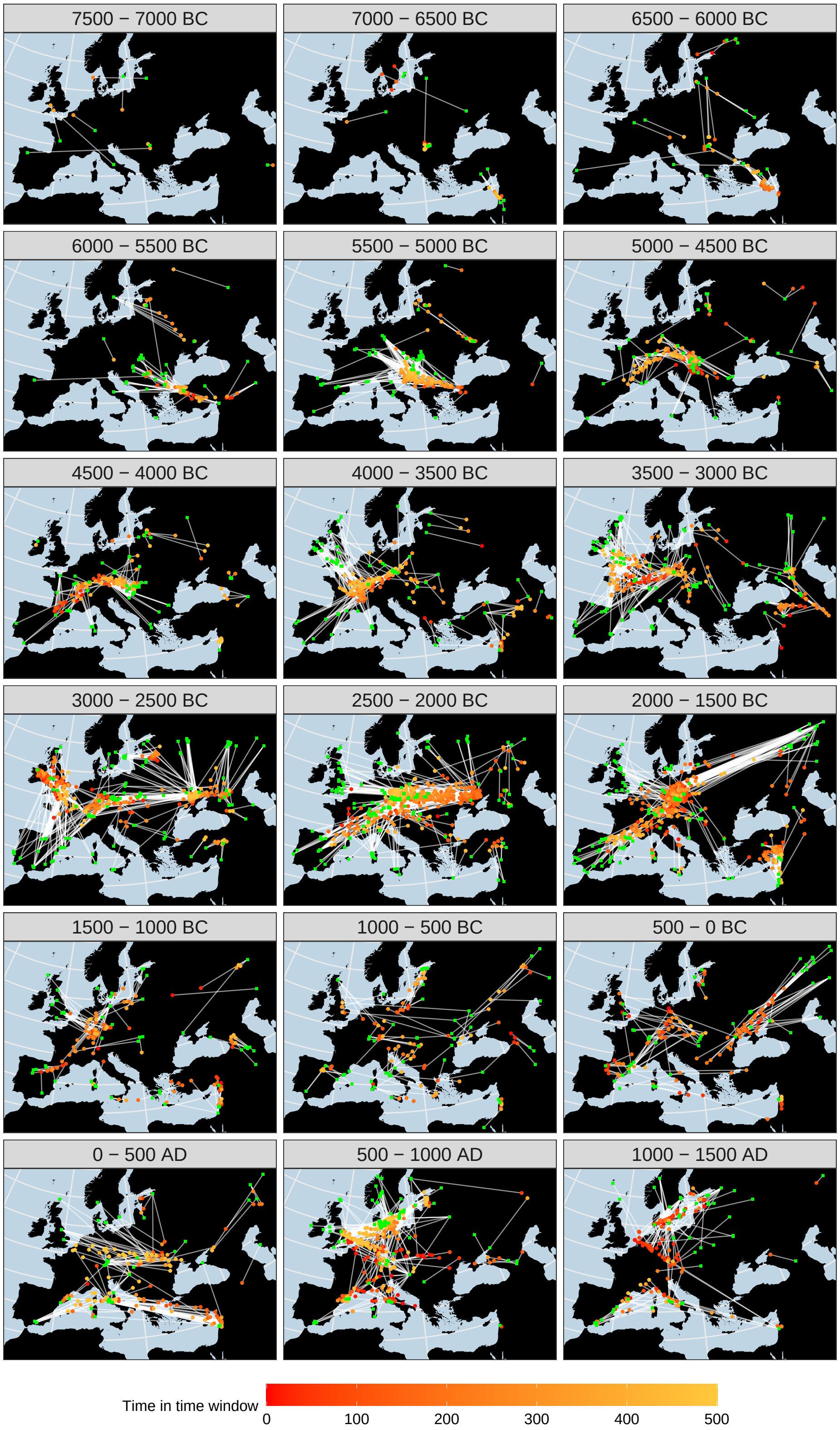


**Figure S16**: Another view on the data in Figure S10. Each subplot in the plot matrix covers a 500-year time window, where each mobility vector is shown as a white line, connecting the sampling location (so usually the place of death) in green with the reconstructed point of highest genetic similarity one default retrospection distance in the past. These points are coloured by a gradient indicating sample age within the respective 500-year time window. This is helpful to see some processes, for example in the 2500-2000BC time window.

# B Meta information for the Datasets S1, S2 and S3

## Dataset S1: Sample context information

Lists context information for all individuals/samples that went into the analysis.

1. **Sample_ID**: An identifier for the individual/sample (taken from the AADR's "Version ID")
2. **Genetic_Sex**: Genetic sex as listed in the AADR
3. **Group_Name**: A "population"/group the individual is attributed to in the AADR dataset (AADR: "Group ID")
4. **Publication**: Publication from which the data for the respective sample was taken. The short publication keys are resolved on the AADR website: https://reich.hms.harvard.edu/allen-ancient-dna-resource-aadr-downloadable-genotypes-present-day-and-ancient-dna-data (AADR: "Publication"). The publications providing the samples are also listed in section D at the end of this document
5. **Country**: The modern day country where the sample was recovered (AADR: "Country")
6. **Region**: The spatial macroregion the sample is coming from (as defined for this paper, see Figure 1)
7. **Latitude**: Latitude of sample location (AADR: "Lat.")
8. **Longitude**: Longitude of sample location (AADR: "Long.")
9. **Date_BC_AD_Start**: Likely starting point of the age range of the sample. Negative values indicate years BC, positive values years AD
10. **Date_BC_AD_Median**: Likely center point of the age range of the sample
11. **Date_BC_AD_Stop**: Likely end point of the age range of the sample
12. **Date_C14**: Radiocarbon dates recorded for this sample in the AADR
13. **Age_Group**: Millennium into which **Date_BC_AD_Median** falls

For the dating information we parsed the AADR column *Full Date: One of two formats. (Format 1) 95.4% CI calibrated radiocarbon age (Conventional Radiocarbon Age BP, Lab number) e.g. 2624-2350 calBCE (3990±40 BP, Ua-35016). (Format 2) Archaeological context range, e.g. 2500-1700 BCE.* Contextual, archaeological age ranges are directly represented here in **Date_BC_AD_Start** and **Date_BC_AD_Stop**, with **Date_BC_AD_Median** as the center point of a uniform distribution between start and end. When radiocarbon dates were available (listed in **Date_C14**), we recalibrated them with the IntCal20 calibration curve to determine the 95.4% range and the center of the post-calibration probability density distribution for **Date_BC_AD_Start**, **Date_BC_AD_Stop** and **Date_BC_AD_Median**. Multiple dates were combined as a simple, normalized sum of said distribution ("sum calibration").

## Dataset S2: Results of multivariate analysis

Shows the result coordinates for the multivariate analysis with EMU, MDS and PCA. **Sample_ID** is shared with Dataset S1. The remaining 80 columns emerge as the product of ten output dimensions (C1-C10), four multivariate analysis methods (EMU, MDS, PCA, Projection PCA) and two SNP sets (unfiltered "$u$", filtered "$f$"). Each column name encodes this parameter combination as follows: **{output-dimension}_{multivar-method}_{SNP-set}** (e.g. C1_mds_u, C3_pca_proj_u).

## Dataset S3: Results of the large-scale mobility estimation

Includes summary statistics for the large mobility estimation run. See Supp. Text 3 for more details on this algorithm. **Sample_ID** is shared with Dataset S1. The columns from column 5 onwards appear in multiple iterations for the permutations of spatiotemporal dimensions, multivariate analysis methods and retrospection distances. All values are rounded to full integers.

1. **Sample_ID**: An identifier for the individual/sample (taken from the AADR's "Version ID")
2. **search_x**: The spatial x-axis coordinate of the (archaeological) site where a sample was found. Coordinates are rounded and given in kilometres according to EPSG:3035 (ETRS89 Lambert Azimuthal Equal-Area, "European grid") after conversion from the AADR's WGS 84 latitude and longitude coordinates
3. **search_y**: The respective y-axis coordinate
4. **search_z**: Rounded mean age of the sample across the temporal resampling iterations The similarity search was repeated in many iterations with different ages drawn from the age range probability distributions. search_z is the rounded mean of these values
5. **field_**[xyz]**_{multivar-method}_{retrospection-distance}**: Mean (across the temporal resampling iterations) spatiotemporal coordinates of the field point with highest similarity probability: The mean end point of the mobility vector
6. **ov_**[xy]**_{multivar-method}_{retrospection-distance}**: Mean (across the temporal resampling iterations) length of the mobility vector in x or y direction
7. **ov_dist_{multivar-method}_{retrospection-distance}**: Mean (across the temporal resampling iterations) length of the mobility vector. See Supp. Text 3 for more details on how exactly this mean is calculated.
8. **ov_dist_se_{multivar-method}_{retrospection-distance}**: Standard error of the mean of all temporal resampling iteration mobility vector lengths
9. **ov_dist_sd_{multivar-method}_{retrospection-distance}**: Standard deviation of all temporal resampling iteration mobility vector lengths
10. **ov_angle_deg_{multivar-method}_{retrospection-distance}**: Direction of **ov_dist** as an angle in degree $(0 - 360°)$

# 1 Supplementary Text: Creating a simplified genetic space

For the analysis in this paper it was necessary to derive simplified, genetic ancestry components for each ancient DNA sample that should be considered in the spatiotemporal model. Each sample should be genetically positioned with coordinates in an $n$-dimensional space, where $n$ is far smaller than the several hundred thousand single nucleotide polymorphisms (SNPs) potentially available for it. Such dimension-reduction is a common application in archaeogenetics, where multivariate analyses are usually employed to make complex admixture patterns readily accessible for visual inspection. Among the most popular methods is principal component analysis (PCA) with modern reference samples, onto which ancient samples are mathematically projected [1].

## 1.1 Finding the most suitable multivariate analysis method

We explored different ways of dimension reduction, and different numbers of target dimensions $n$ . We limited our search to $n \leq 10$ and the following four methods:

- MDS as implemented in plink v1.9 [2] using 1-IBS pairwise distances
- PCA as implemented in the smartsnp R package v1.1 [3] with simple mean-frequency imputation of missing values
- Projection PCA as implemented in smartsnp with a set of modern, Western Eurasian reference populations extracted from the AADR dataset
- EMU, a PCA implementation with significantly more sophisticated imputation of missing values compared to PCA. Provided by the emu command line tool v0.9 [4]

Figure S2 shows the scatter plots of the first (C1) and the second (C2) output dimension for these methods. The subplots **E** and **F** already indicate that simple PCA with mean imputation is not capable to distinguish spatiotemporal clusters as clearly as the other methods, which we think is due to the underperforming imputation of missing data in ordinary PCA using mean allele frequencies. Experiments with the correlation and out-of-sample prediction analysis below confirmed this observation: Simple PCA performed worse there by a factor of 2 to 3. We therefore decided to exclude this method right away from further consideration.

We also saw a clear separation of samples that were prepared via untargeted shotgun sequencing and samples that went through a target-enriching capture preparation step (usually for the 1240K SNP set) on the third output dimension of both the MDS and the EMU analysis (Figure S3). This effect was already highlighted by Margaryan et al. 2020 (Supplementary Note 8 - Genetic clustering) [5]. In an attempt to mitigate the effect of this undesired, as for our analysis irrelevant, cofactor, we applied a simple association analysis (`plink --assoc`) to identify and remove SNPs from our input dataset, that are significantly correlated with the shotgun vs. capture variable ($p < 0.001$).

That left us with the following SNP filter workflow and two main SNP sets for the comparison analysis:

| | # of samples | # of SNPs | Identifier SNP set |
|---|---|---|---|
| Starting point (AADR V50.0, 1240K) | 10391 | 1233013 | |
| - Selecting spatiotemporally relevant samples<br>- Removing samples below data quality threshold (SNP count, contamination)<br>- Genomic range filtering according to [6, 7] (see Materials and Methods in the main text)<br>- Removing SNPs below a 1% minor allele frequency threshold | 3530 | 963289 | |
| - Removing samples from related individuals<br>- Removing SNPs below a 5% maf threshold | 3191 | 847053 | **u**nfiltered SNP set |
| - Removing SNPs that are associated to shotgun vs. capture data preparation<br>- Removing samples now below SNP count threshold | 3138 | 705367 | **f**iltered SNP set |

To make an informed decision about which of the remaining multivariate analysis methods (MDS, Projection PCA or EMU), number of dimensions $n$, and SNP set (unfiltered or filtered) to use, we employed three quantitative measures that, as we argue below, are informative on the suitability of a given genetic space to allow for good similarity probability estimates with a spatiotemporal model as desired for this analysis:

- Normalised mean Euclidian distance in the reduced genetic space for very low spatiotemporal distance pairs (the "nugget")
- Correlation of pairwise (reduced) genetic and spatiotemporal distance
- A normalized measure of true and estimated distances in the reduced genetic space (according to the eventually desired spatiotemporal interpolation model)

Figure S17 summarizes the results for these metrics.

The nugget term in S17 **A** is introduced in more detail below in Supp. Text 2. It is calculated independently for every output dimension of the respective multivariate analysis and functions as a normalized proxy for pairwise genetic distances of samples that are close in space and time (often even from the same archaeological site or burial context). For this plot the nuggets are determined not directly from the (genetic) output coordinates, but for the residuals of a linear model (see Supp. Text 3). As they are computed from pairs of samples close-by in space and time, nuggets are a direct estimate of local noise in the reduced genetic space. We generally observe lower nuggets for the first output dimensions compared to more derived ones, which indicates that the first dimensions have a higher signal to noise ratio. The increase of the nugget along the dimension count is not linear, though, with different growth patterns for the different multivariate methods.

The measure in Figure S17 **B** is calculated as the correlation of pairwise "genetic" distance (Euclidean distance in the multivariate output dimension space up to dimension $n$, so e.g. C1-C7 for C7), and pairwise spatiotemporal distance (Euclidean spatiotemporal distance scaled with 1 year = 1 kilometre) (e.g. Figure S18). We report the $R^2$ value to summarise the output, so a higher value indicates a stronger correlation. All methods perform generally well, and higher-dimensional spaces seem to be linked to some degree to higher correlations for MDS and EMU up to C7. This does not necessarily contradict the results for the nugget term: Here long-distance correlation dominates the result, which is deliberately omitted in the nugget. We also observe, that the filtered SNP set performs consistently worse in all instances of this correlation test, probably because the filter is not perfect and also removes valuable information.

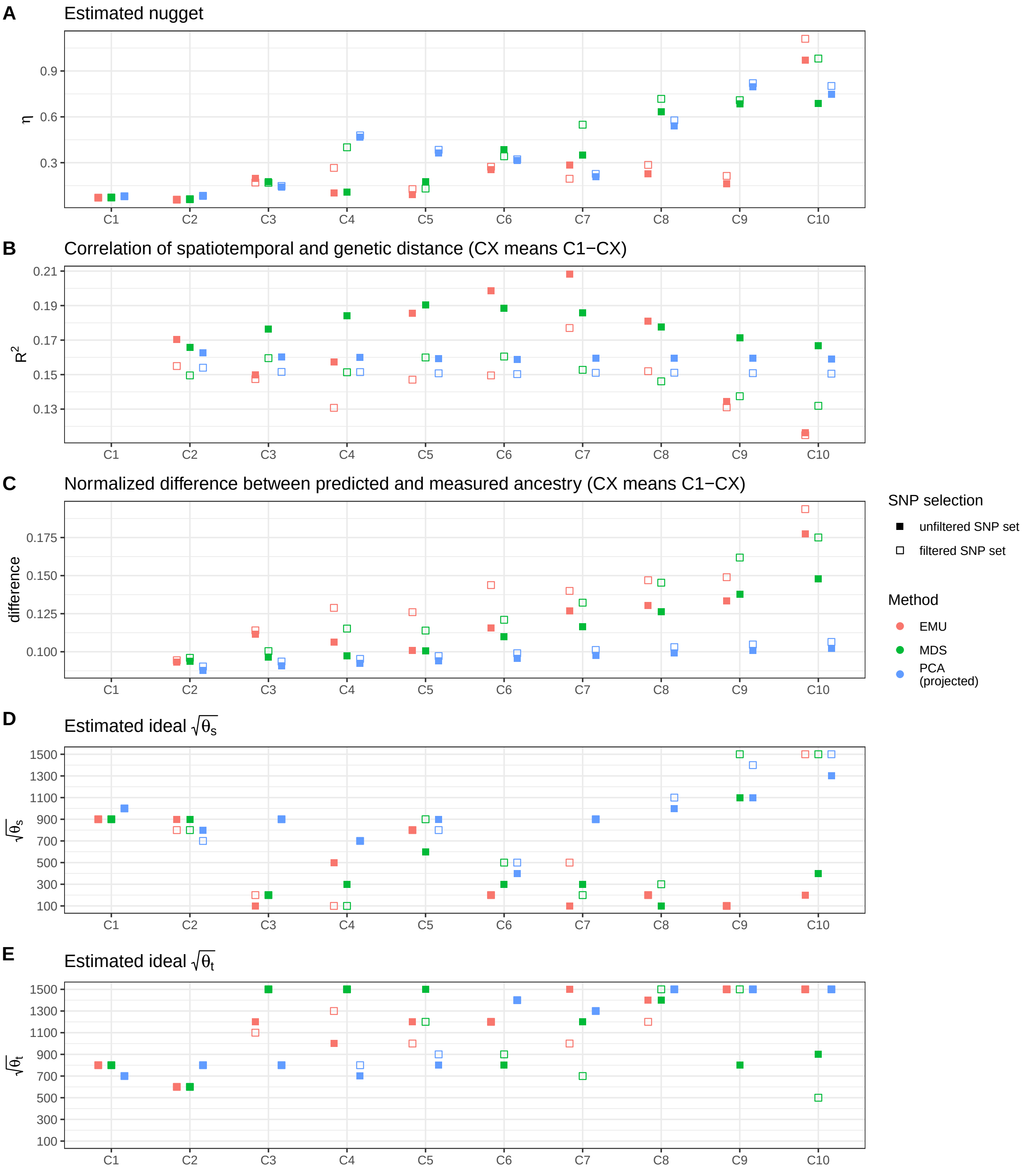


**Figure S17**: Various measures to compare differently calculated genetic spaces.

Figure S17 **C** summarises the measure we ultimately consider the most informative: The predictive accuracy of a spatiotemporal Gaussian process regression model in a cross-validation setup as explained in Supp. Text 2. Each dimension for each multivariate method is modelled independently with the ideal nugget and kernel parameter setting for a 9/10 training dataset. We then compare the difference of actual and predicted values for a 1/10 test dataset through the multivariate output dimension space (again: for example

C7 on the x-axis means C1-C7). The differences shown in Figure S17 **C** are normalised by the mean pairwise distance in said space to make them comparable across methods (a similar normalisation as done in the calculation of the nugget). Projection PCA performs best by that metric, especially for higher-dimensional spaces. EMU and MDS lose accuracy quickly, the former already for C1-C3, the latter after C5. Note that the general increase of values towards more included dimensions is expected due to the "curse of dimensionality".

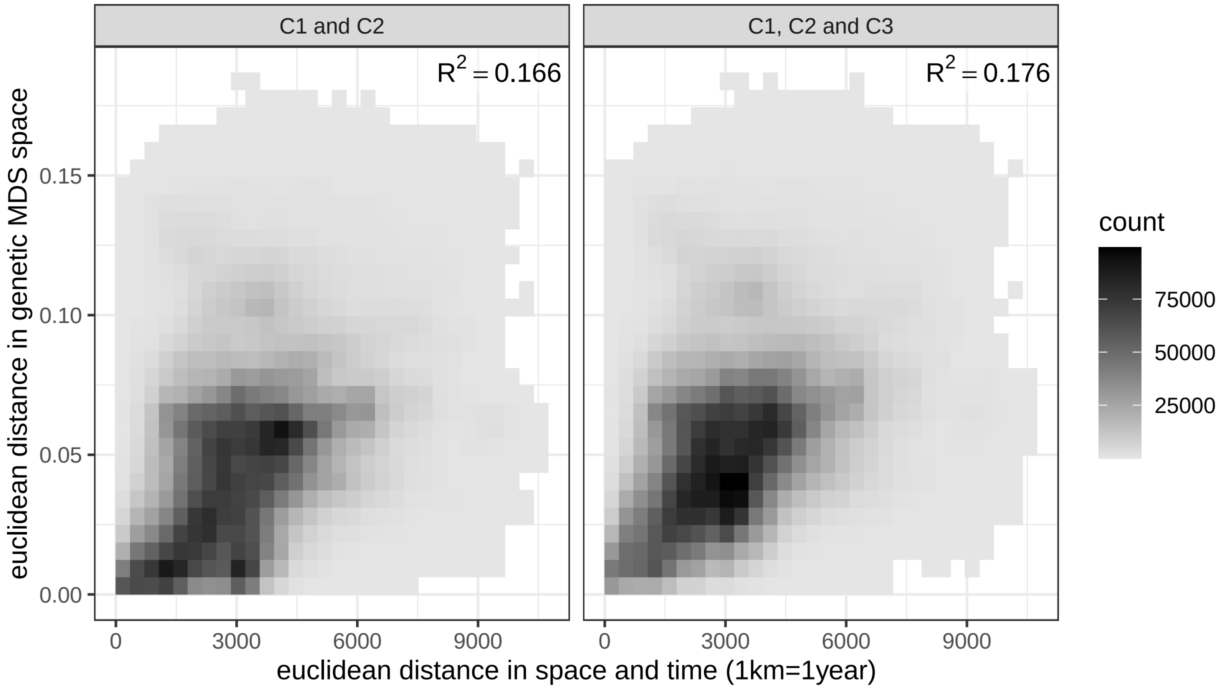


**Figure S18**: Correlation of pairwise genetic and spatiotemporal distance with genetic distance in two- or three-dimensional MDS space. The pairwise distances are counted in bins and plotted as a density raster.

To conclude, the observations for these three measures together do not necessarily lead to an obvious decision which multivariate analysis method, $n$, and SNP set is optimal for the spatiotemporal mobility estimation we want to attempt. In an iterative process we could rule out some options, though:

The reduced, filtered SNP set performs almost always worse than its unfiltered counterpart. It clearly avoids some of the shotgun vs. capture bias, but there seems to be spatiotemporal information encoded exactly in this distinction – maybe through a complex interaction of the archaeological record, preservation and research history. We seem to be better off with the additional 140,000 SNPs and decided to abandon the filtered dataset.

The question which multivariate method to use is harder to decide – at least for low-dimensional spaces. Our understanding is, that Projection PCA, EMU and MDS generally perform similarly well on C1 and C2, with various local optima where one method trumps the others. Here we resorted to external factors, like the complexity of the underlying algorithm and the amount of additional parameters necessary for a given method, assuming that simpler and less is generally preferable. EMU employs a relatively complex imputation algorithm on top of normal PCA, and Projection PCA requires a set of modern reference populations, which has critical influence on the genetic space it generates. We therefore decided to rely on MDS (**MDS2**) for the analysis presented in the main text. This includes the implicit assumption that the method would produce similar and robust results even with other pairwise distance metrics beyond the 1-IBS measure we employed.

A forced limitation to C1 and C2 has the additional advantage that a 2D ”genetic map” is relatively easy to visualize and understand – one can intuitively conclude that its structure is meaningful on the spatial and temporal scale of our analysis.

For higher dimensions beyond C2, MDS and EMU can be ruled out entirely due to the extreme bias the shotgun vs. capture distinction introduces (Figure S3) and which we could not reliably cancel out via SNP filtering. Projection-based PCA has a massive advantage here, as it relies on an optimized, external data source to inform the structural properties of the genetic space. It is robust for all the ten dimensions we tested, and adding more dimensions could barely deteriorate correlation or predictive accuracy. It is unclear though, how many of these additional components $n$ add value to the similarity search implemented for this paper. Figure S8 explores this question with a set of test individuals and search settings. Adding dimensions beyond C3 does not visibly change the respective probability landscapes, but the position of maxima can suddenly change, if there are multiple relevant peaks. It is likely that this effect would continue also beyond C10. Based on the observation that the estimated values for $\sqrt{\theta_s}$ and $\sqrt{\theta_t}$ seem to follow a different dynamic from C5 onwards for the PCA (Figure S17, **D** & **E**), which indicates some change in the setup of these variables, we decided to only consider C1-C5 (**PCA5**) for a second run of the mobility curve determination for Western Eurasia (beyond **MDS2**).

# 2 Supplementary Text: Interpolation parameter estimation

A key component of this paper is the interpolation of a genetic ancestry field based on the output dimensions of different multidimensional analysis methods. Here, we use Gaussian process regression, which is a parameterised method. The following section explains the process we went through, to find an optimal set of parameters. For increased clarity the plots only show the results for the first two or three output dimensions of our MDS run with the unfiltered SNP set (see Supp. Text 1). As discovered above, the third dimension is highly biased by the library preparation (capture vs. shotgun) and not directly correlated to space and time. When we include it below, then only as a didactic reference point.

We consider a number of individuals distributed in space and time, with a single-dimensional (scalar) genetic MDS (or PCA) component as dependent variable. We use the notation $(x_i, y_i, t_i, g_i)$ to denote for each data point $i$ the set of spatial coordinates ($x_i$ and $y_i$), an age $t_i$ and the value of the genetic component $g_i$.

We intend to model our data points as a random Gaussian process, for which we are using the laGP R package for local approximate Gaussian process regression [8]. As a technical note, one of the assumptions in this package is a mean of zero in the Gaussian process, which we exactly achieve by first fitting a linear model to the data, and then considering the *residuals* instead of the original genetic values.

In mathematical terms, the model including the linear fit can be presented as

$$g \sim ax + by + ct + g'(x, y, t) \tag{1}$$

where $g'$ reflects a mean-zero random field, which we model with GPR. For simplicity, and because this is a one-time operation, we just continue using the notation $g_i$, now actually denoting the residuals of the linear model instead of the raw genetic component.

A key ingredient for Gaussian process regression is the covariance kernel function, for which we here follow the standard choice of a squared exponential, which in general terms for p-dimensional input data and in the notation of laGP is defined as

$$Cov(x, x') = \tau^2 \left( \exp\left[ -\sum_{k=1}^{p} \frac{(x_k - x'_k)^2}{\theta_k} \right] + \eta\delta(x - x') \right) \tag{2}$$

where $x$ and $x'$ are positions in p-dimensional space, and $\theta_k$ are lengthscale parameters for each dimension $k$. $\eta$ is a dimension-less additional noise term to be added only if $x = x'$, using the delta-distribution, the so called nugget term. $\tau^2$ is a general scaling parameter.

Specifically for the purpose of spatio-temporal modelling with isotropic space, we write this covariance function as

$$\mathrm{Cov}(r, u) = \tau^2 \left( \exp\left[ -\left(\frac{r}{\rho}\right)^2 - \left(\frac{u}{\alpha}\right)^2 \right] + \eta\delta(x - x') \right) \tag{3}$$

where we have changed notation slightly, and introduced the spatial kernel radius $\rho$ and the temporal kernel radius $\alpha$, which by construction have now length- and time-dimensions (measured in years and kilometres, respectively).

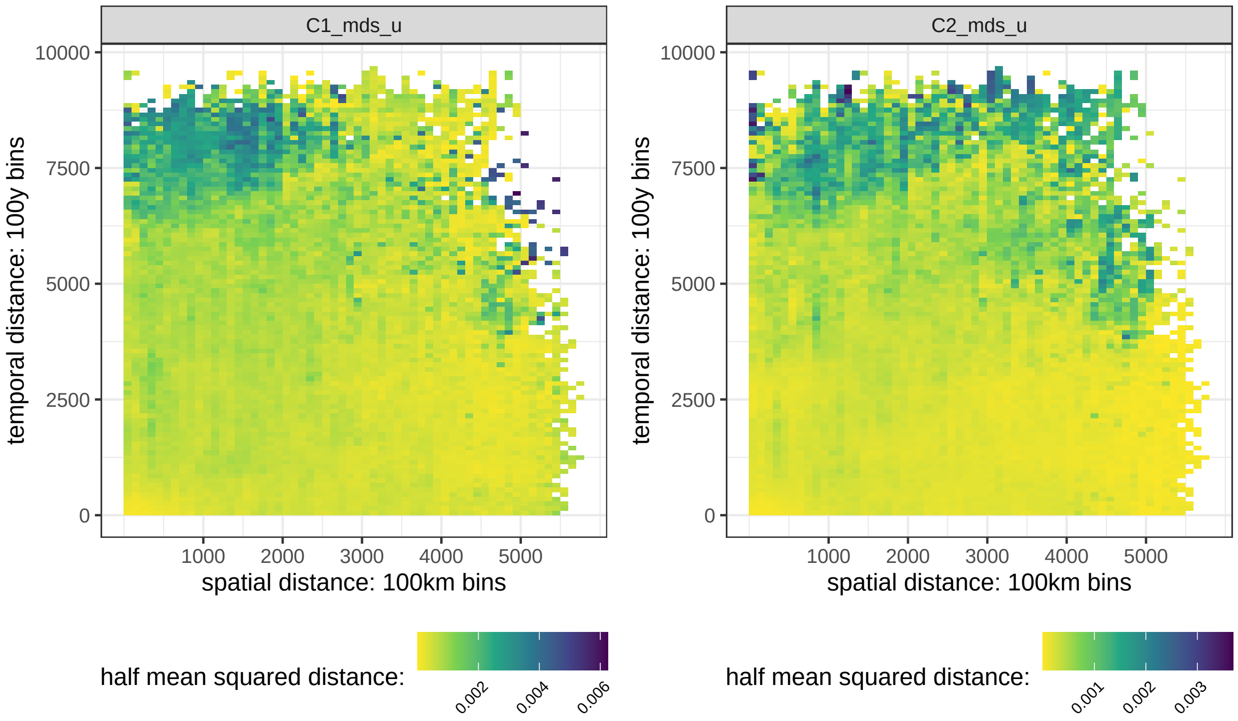


**Figure S19**: Empirical semivariogram rasters calculated including all samples in the analysis dataset, with one plot for each ancestry component (MDS coordinate) C1 and C2. The fill colour represents the mean squared pairwise distance in the respective space-time bin. For some basic detrending these distances were calculated not directly on the ancestry components, but on the residuals of a simple linear model, where the genetic coordinates for each sample are predicted by their spatiotemporal position.

## 2.1 Variogram analysis

One possibility to inspect plausible parameters for $\tau$, $\eta$, $\rho$ and $\alpha$ as defined in 3 is variogram analysis (see also [9], [10]).

It is instructive to first consider variograms in the context of continuous fields, where the field value $g(x,t)$ is defined at all spatial points $x$ (which in our case are two-dimensional) and all time points $t$. The semivariogram is then defined as the mean squared difference of field values at given spatial and temporal distances:

$$V(r,u) = \frac{1}{2}\langle (g(s,t) - g(s+r,t+u))^2 \rangle_{s,t} \tag{4}$$

where the average runs over all space-time points $(s,t)$.

Under the assumption of constant variance $\langle g(s,t)^2 \rangle = \langle g(s+r,t+u)^2 \rangle$ for all $s,r,t,u$, we can establish the relationship of the semivariogram and the covariance function of the Gaussian process:

$$
\begin{aligned}
V(r,u) &= \frac{1}{2}\langle (g(s,t) - g(s+r,t+u))^2\rangle_{s,t} \\
&= \frac{1}{2}\langle g(s,t)^2 - 2g(s,t)g(s+r,t+u) + g(s+r,t+u)^2\rangle \\
&= \frac{1}{2}\left(\langle g(s,t)^2\rangle - 2\langle g(s,t)g(s+r,t+u)\rangle + \langle g(s+r,t+u)^2\rangle\right) \\
&= \frac{1}{2}\left(Cov(0) - 2Cov(r,u) + Cov(0)\right) \\
&= \mathrm{Cov}(0) - \mathrm{Cov}(r,u)
\end{aligned}
\tag{5}
$$

with $\mathrm{Cov}(r,u) = \langle g(x,t)g(x+r,t+u)\rangle$

So the variogram is directly related to the covariance of the Gaussian process:

$$
V(r,u) = \mathrm{Cov}(0) - \mathrm{Cov}(r,u) \tag{6}
$$

Following ref. [10] (p.30), the **empirical semivariogram**, defined for a set of actual datapoints, can be computed as a binned version of the continuous semi-variogram definition employed above. Specifically, instead of continuous spatial and temporal "radius" values $r$ and $u$, as in the continuous version, we now consider bins $R_k = (r_k, r_{k+1})$ and $U_l = (u_l, u_{l+1})$, with boundaries $r_1 < r_2 < \ldots$ and $u_1 < u_2 < \ldots$. We then write

$$
V(k,l) = \frac{1}{2N(k,l)}\sum_{i,j}(g_i - g_j)^2 I\left(\sqrt{(x_i - x_j)^2 + (y_i - y_j)^2} \in R_k, |t_i - t_j| \in U_l\right) \tag{7}
$$

where $I$(condition) is an indicator function that is 1 if the condition is true and zero otherwise, and the normalization $N$ is

$$
N(k,l) = \sum_{i,j} I(\sqrt{(x_i - x_j)^2 + (y_i - y_j)^2} \in R_k, |t_i - t_j| \in U_l) \tag{8}
$$

Figure S19 is one way to visualize this empirical semivariogram $V(k,l)$ as a raster plot. The bins $R_k$ and $U_l$ are here chosen such that $r_i - r_{i-1} = 100$km and $u_l - u_{l-1} = 100$years.

To finally determine the kernel parameters from equation 3, one needs to fit the variogram using the kernel covariance function, thereby learning the four kernel parameters $\tau^2$, $\eta$, $\rho$ and $\alpha$. We realised that in the case of our data, this was unfortunately not possible, as we cannot co-estimate Cov(0) and the kernel radiuses simultaneously. Consider Figure S20, which shows only a single cut through the semivariogram. In this case, we aim to fit three parameters from this curve (since we consider only the temporal dimension now). The squared exponential form has three features that we expect to see in the semivariogram: i) An offset at $t = 0$ (the left hand side of the variogram), ii) the scale of the increase towards larger values of $t$, and iii) the height of the plateau of the semivariogram. These three features are related to the three parameter we seek to fit. However, the expected plateau is in our case however never reached. Instead, the covariances in the genetic space continue to increase towards larger values of temporal distance $t$. A similar effect is seen in one-dimensional cuts through the spatial component of the semivariogram. We believe the lack of an implicit scale in the semivariogram suggests the presence of many different temporal and spatial scales in human genetic relatedness (due to various evolutionary processes and mobility acting also on multiple scales), which precludes estimating a kernel width from the semivariogram directly. The degeneracy of the

semivariogram can be directly demonstrated by fitting two kernel models with very different parameters in Figure S20.

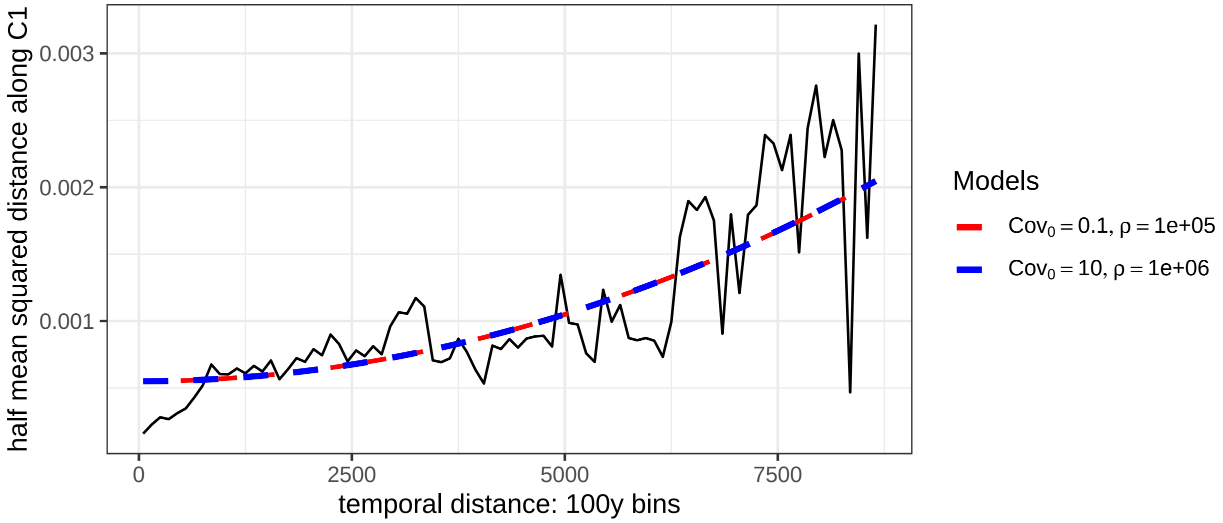


**Figure S20**: A variogram for one time slice ($x \in [0, 100]$) with two different, but equally well fitting exponential models.

Indeed: For $(r/\rho) \ll 1$ we have

$$\begin{aligned}\mathrm{Cov}_0 - \mathrm{Cov}_0 * \exp(-(r/\rho)^2) &= \\ \mathrm{Cov}_0 \left(1 - \exp(-(r/\rho)^2)\right) &\approx \\ \mathrm{Cov}_0 \left(1 - (1 - (r/\rho)^2)\right) &= \\ \frac{\mathrm{Cov}_0}{\rho^2} r^2 &\end{aligned} \tag{9}$$

where we have used the Taylor expansion for the exponential function: $\exp(x) = 1 + x + \mathcal{O}(x^2)$. So for small values of $r/\rho$ (i.e. long before a plateau gets reached) we get an approximate squared function with a coefficient of $\mathrm{Cov}_0/(\rho^2)$, which shows that the model is approximately invariant under changes of $\mathrm{Cov}_0$ and $\rho^2$ that leave the ratio constant. This is the case in the two curves above. We don't get into the plateau of the variogram, so can not fit $\mathrm{Cov}_0$ and $\rho$ independently. We concluded that empirical variograms can not be used for kernel length estimation in this particular context, and turned to different estimation approaches below.

However, the variogram at least exposes an approach to estimate the variance $\tau^2$ and nugget term $\eta$ (as in equation 2). First, from the form of the covariance function 3 we have $\mathrm{Cov}(0, 0) = \tau^2(1 + \eta)$. At the same time, for small but non-zero values of $r$ and $u$ we have $\mathrm{Cov}(r \to 0, u \to 0) = \tau^2$. So for the semivariogram we get:

$$V(r \to 0, u \to 0) = \mathrm{Cov}(0, 0) - \mathrm{Cov}(r \to 0, u \to 0) = \tau^2(1 + \eta) - \tau^2 = \tau^2\eta \tag{10}$$

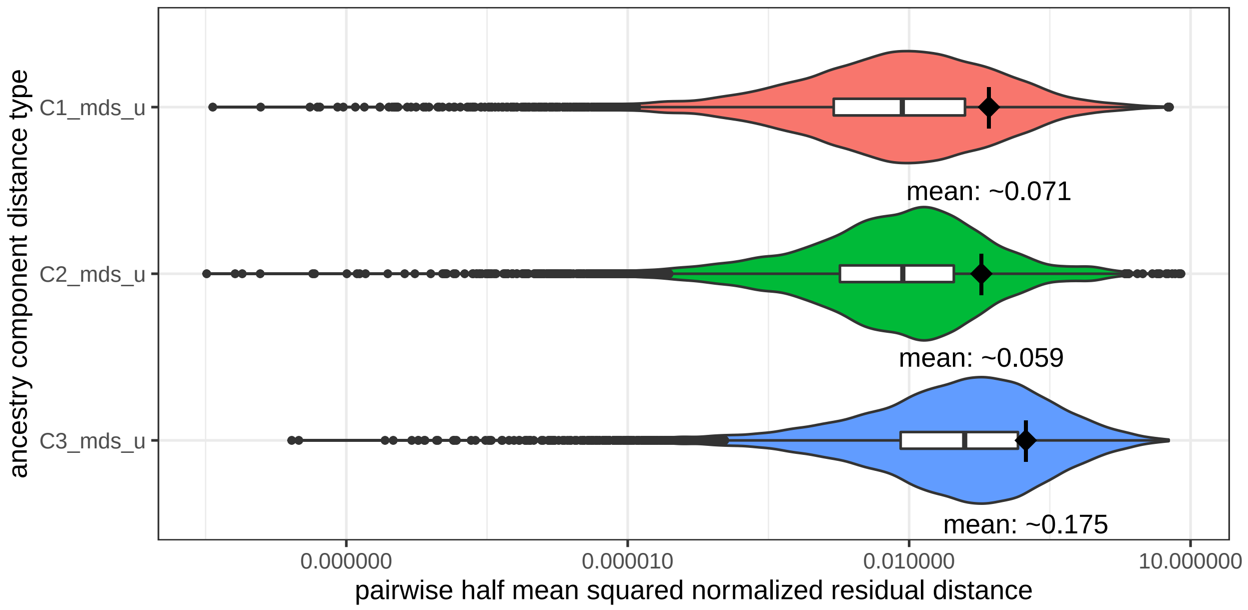


**Figure S21**: Violin- and boxplot of the detrended pairwise distance distribution for different ancestry components in a short and narrow temporal and spatial distance window ($<$ 50km & $<$ 50years). The diamond shaped dot is positioned at the mean point of the distribution.

For the nugget term we have now an estimator

$$\hat{\eta} = \frac{V(r \to 0, u \to 0)}{\tau^2} \tag{11}$$

This can be readily derived, since the variance $\tau^2$ can be estimated as the overall sample variance of the data, i.e.

$$\hat{\tau^2} = \frac{1}{N}\sum_i (g_i - \bar{g})^2 \tag{12}$$

where $N$ is the number of data points and $\bar{g}$ is the mean genetic value.

Figure S21 shows the distribution of pairwise squared genetic distances for samples that are less then 50 years and 50 kilometres apart. Each distance value is scaled according to the estimator defined in equation 11 and the mean of these pairwise distances is a good default for the nugget term of a given ancestry component (see also Figure S17 **A**). So these means are what we used for the nugget terms throughout the paper. Note how the result for the third MDS output dimension is 2-3 times bigger than for the first two. This is certainly a consequence of the low spatiotemporal correlation of this variable uncovered in Supp. Text 2.

## 2.2 Maximum likelihood estimation

As a second method for kernel-width estimation, we turn to maximization of the likelihood. The laGP package [8] provides two different maximum likelihood estimation (MLE) algorithms for automatic kernel parameter exploration in anisotropic spaces: `mleGPsep` and `jmleGPsep`. According to the manual, `mleGPsep` uses L-BFGS-B optimization (a limited memory quasi-Newton approximation of the Broyden-Fletcher-Goldfarb-Shanno algorithm) to get an estimate of $\theta$ ($\rho$ and $\alpha$ above). It allows for joint estimation of $\theta$ and the nugget $\eta$ with a common gradient. `jmleGPsep` on the other hand is explicitly designed for joint inference by

iterating over the marginals of $\theta$ and $\eta$. laGP allows to set starting parameters and search boundaries for both algorithms with the helper functions `darg` and `garg`. According to the manual, these ”leverage crude summary statistics” over the independent and dependent input variables to define sensible defaults.

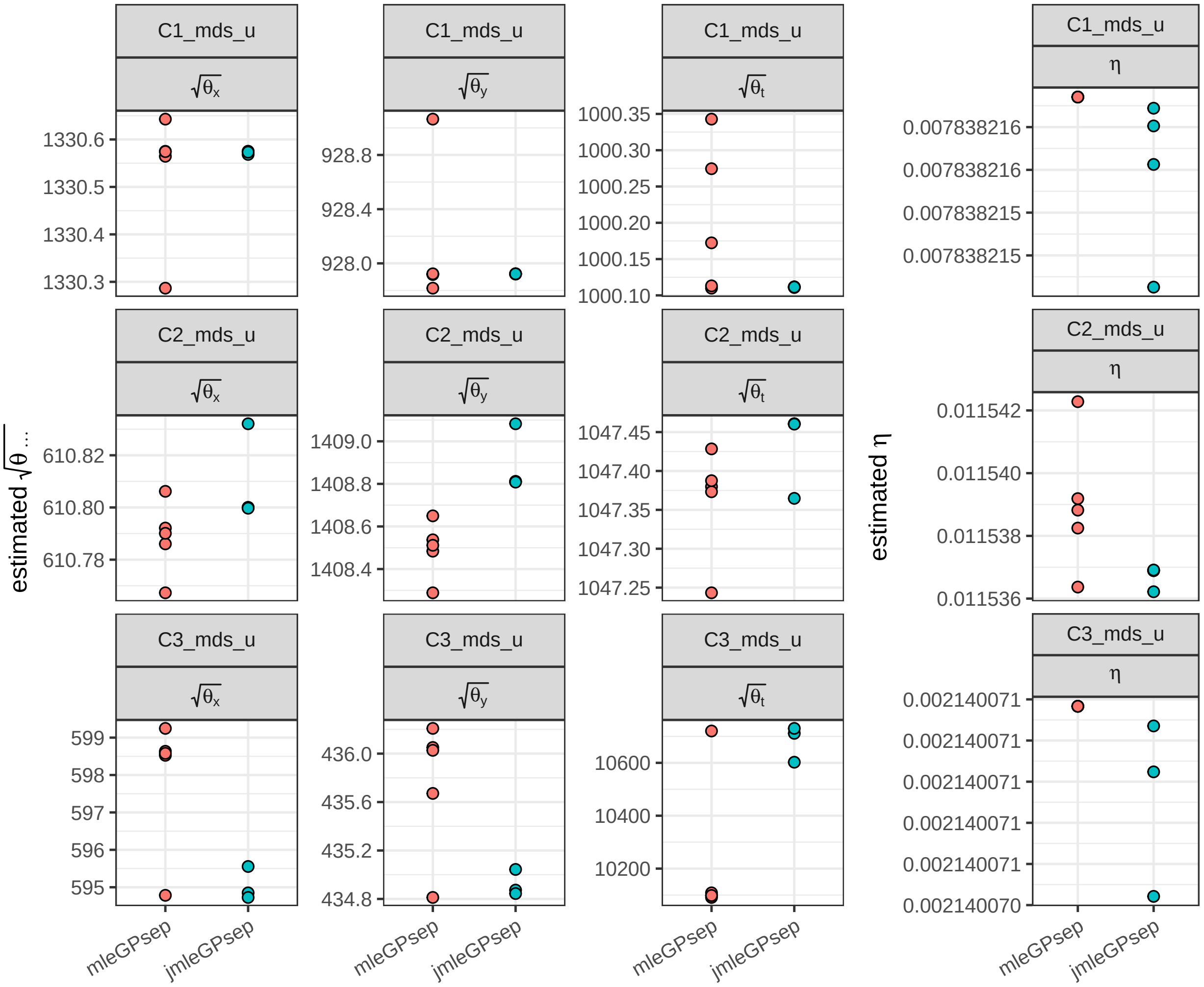


**Figure S22**: Results of the kernel parameter estimation with the laGP maximum likelihood algorithms `mleGPsep` and `jmleGPsep`. Each dot represents the result of one run for one parameter. For each permutation of algorithm and ancestry component 5 runs were calculated.

`mleGPsep` and `jmleGPsep` as implemented in laGP are generally not well suited for spatiotemporal data without inherent latitudinal or longitudinal bias, as they optimize each input dimension separately: Instead of one spatial kernel radius $\theta_s$ and one temporal kernel radius $\theta_t$, they effectively yield two separate values for $\theta_s$, one for the spatial x axis ($\theta_x$), and one for the spatial y axis ($\theta_y$). Despite this, we decided to apply the algorithms here to get a first estimate for $\theta$ and to test our previous conclusion concerning $\eta$.

Figure S22 shows the result of multiple runs for each combination of algorithm and ancestry component. The estimated $\theta$ values for the three dimensions are very similar between the two algorithms (`mleGPsep` and `jmleGPsep`) but different for the three ancestry components modelled with the Gaussian process (C1, C2 and C3 in MDS space). Note that we report $\sqrt{\theta}$ instead of $\theta$, since that has the more interpretable unit

(kilometres and years, respectively), see equation 2. The values for the first two MDS output dimensions are relatively large, but seem at least generally plausible, given how far the influence of each point could "radiate" in a squared exponential model and how far prehistoric interaction networks may have spanned (see Figure S23 to get some intuition). This does not hold for the biased MDS dimension C3, where $\sqrt{\theta_s}$ is estimated to be about 20 times smaller than $\sqrt{\theta_t}$.

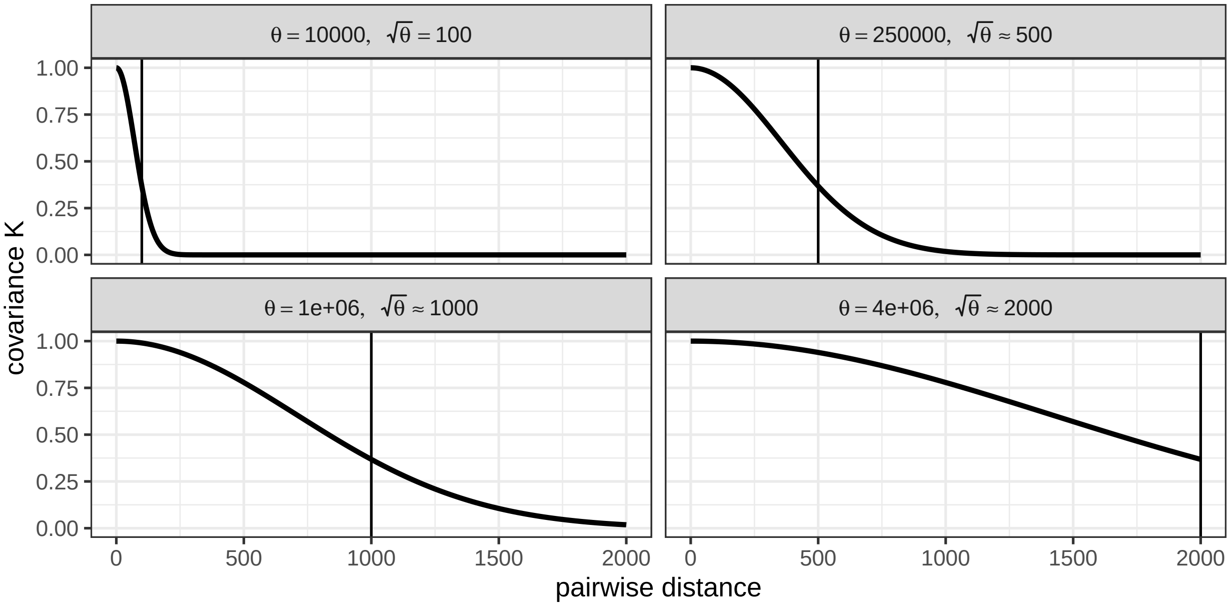


**Figure S23**: Example curves to illustrate the behaviour of a squared exponential function $K_{ij} = \exp(\frac{-\|x_i - x_j\|^2}{\theta})$ with different values of $\theta$. The "pairwise distance" could for example be in kilometres or years.

One consistent observation across all three ancestry components considered here, is that $\theta_x$ should be different from $\theta_y$ for an optimal model. So the above mentioned anisotropy issue does indeed affect the outcome of the parameter estimation and poses a form of overfitting. We do not believe though that a model with a latitude-longitude mismatch is justified in this context.

The estimated values for $\eta$ are about one order of magnitude smaller than the ones estimated from the variogram. We assume this to be an effect of the implausible anisotropy. Experimental interpolation test runs with $\eta < 0.04$ led to overfitting in settings with fixed $\theta_x = \theta_y$ and we therefore decided to keep $\eta$ as decided above.

laGP also provides the function `mleGP` to estimate $\theta$ and $\eta$ in isotropic systems and we decided to employ this algorithm as well. To account for the anisotropic nature of the space-time relationship we introduced a scaling factor that manipulates the temporal axis. Starting from the default 1 (1km = 1y) we increased and decreased the scaling factor in a rescaling sequence from 1/10 to 2. Figure S24 documents the result: `mleGP` yields only one value for $\theta$, which reacts to the forced temporal "contraction" and "inflation". One way to imagine this is a rigid sphere in a changing cuboid universe: We contract or inflate the cuboids z-axis and estimate for each setting i) if a sphere is a good assumption for predicting observations (Figure S24 **B**) and ii) which radius the sphere should ideally have (Figure S24 **A**). As stated above, we used fixed values for $\eta$ here.

In the MDS setup shown here and for the spatiotemporally informative ancestry components C1 and

C2, increasing and decreasing the scaling factor, so temporal inflation and deflation, quickly deteriorates the model likelihood. A scaling factor of 1, so $\theta_t = \theta_s$ and 1km = 1y, yields good results for both. The estimates for the absolute values of $\sqrt{\theta}$ are smaller, but on the same magnitude as for the anisotropic estimation above. For C3 the algorithm does not detect a local optimum within the search space. Just as observed above with the anisotropic mle algorithms, solutions with very small scaling factors, so massively contracted time and therefore $\theta_t \gg \theta_s$ are favoured for this component.

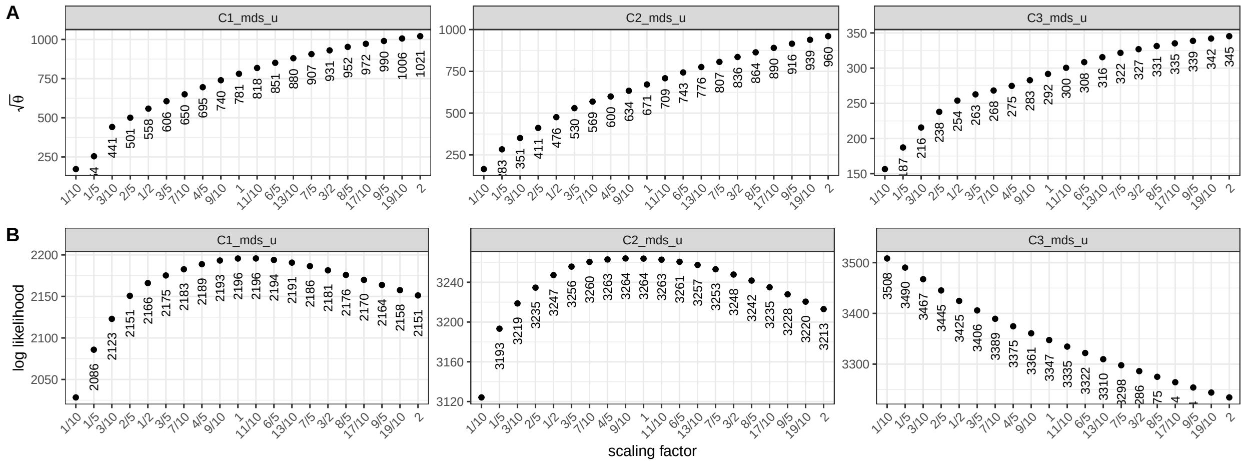


**Figure S24**: Results of `mleGP` exploration runs with variable scaling of temporal and geospatial space. `mleGP` assumes an isotropic sytem.

## 2.3 Crossvalidation

As a third and more independent method to estimate $\theta$, we turned towards a simple crossvalidation approach, which allows to see the effect of different kernel size values on prediction accuracy and precision. We explored a $\theta$ grid with 15 values for the spatial kernel size $\sqrt{\theta_s} = 100, 200, 300, ..., 1300, 1400, 1500$ km and 15 values for the temporal kernel size $\sqrt{\theta_t} = 100, 200, 300, ..., 1300, 1400, 1500$ years. The nugget term $\eta$ was again fixed as decided above. Our crossvalidation algorithm includes the following steps and was applied for each ancestry component and $\theta_s$ and $\theta_t$ combination separately:

1. Randomly reorder the observations
2. Split the observations into 10 groups
3. Build a laGP GPR model from 9 of the 10 groups and use it to predict the 10th. Do this for all combinations of groups
4. Calculate the distance between real and predicted value for each observation

As these steps are also repeated 10 times, this crossvalidation is computationally very expensive and was calculated on a high performance computing cluster. The fast approximate GPR implementation in laGP helped substantially to make this feasible.

Figure S25 shows the distributions of a large sample of normalized (to the range of the ancestry components) distance values. As expected, the distances form a distribution around zero. Most predictions are in a 10% margin around the observed ancestry data. That means that i) the GPR models are generally good

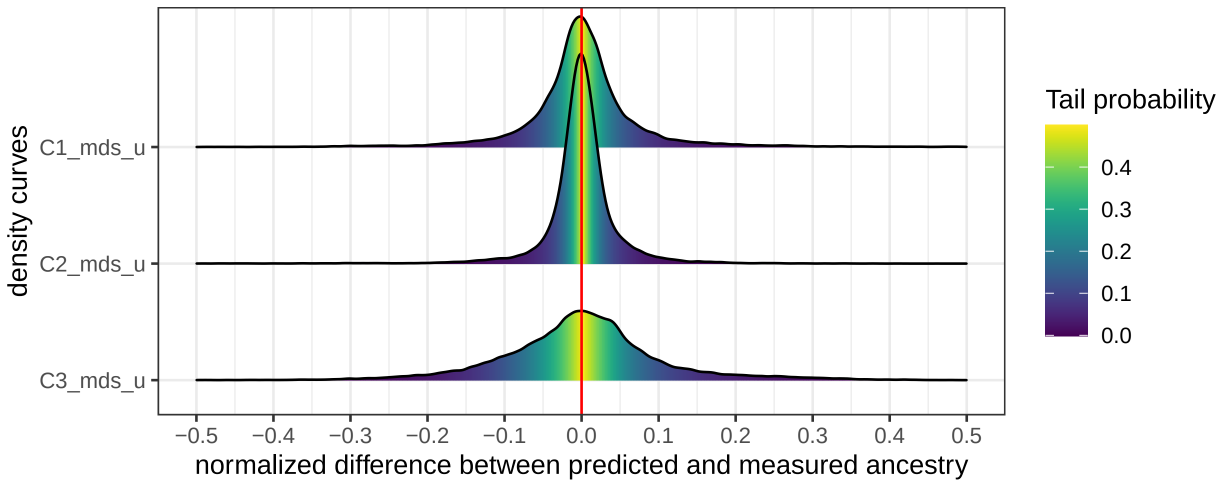


**Figure S25**: Distribution of 200,000 randomly drawn deviations out of all crossvalidation prediction-observation distances. The distance values were normalized to the total range of the respective ancestry component.

at predicting the ancestry of unknown observations and ii) there must exist multiple combinations of $\theta_s$ and $\theta_t$ that yield a solid GPR model.

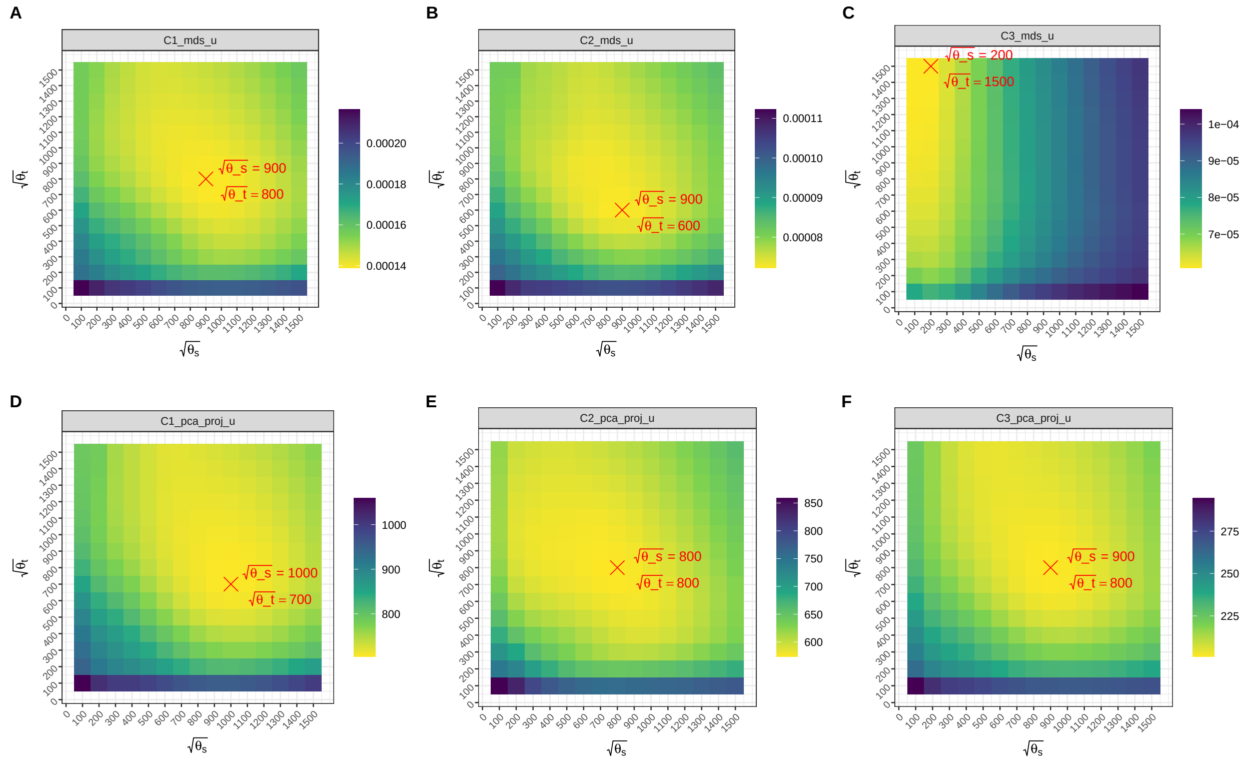


**Figure S26**: Crossvalidation results (mean squared differences between prediction and observation) of the first three ancestry components of MDS and projected PCA for different $\theta_s$ and $\theta_t$ combinations. The combinations of $\theta_s$ and $\theta_t$ with the best mean predictive power are highlighted in red.

The latter is confirmed when we look at the mean squared difference rasters in Figure S26 **A** and **B**. Generally, good predictions are possible in a remarkably large corridor of $\theta_s$ and $\theta_t$ value permutations. The red crosses in the plots mark the lengthscale parameter combination with the best predictive capabilities for the respective ancestry component. The figure also includes the results for the third MDS dimension (**C**) which shows how clearly it deviates in its spatiotemporal behaviour. The results for the first three dimensions of the projected PCA (**D**, **E** and **F**) are added as well, to further illustrate what we already derived from Figure S2 **D** and **E** in Supp. Text 1.

We conclude that large (multiple hundred kilometers and years) kernels with $\theta_t \approx \theta_s$ have the best mean postdictive power for the European spatiotemporal ancestry field given the amount and distribution of the data and the ancestry components (MDS2, PCA5) considered for our study. This was already indicated by maximum likelihood estimation with the laGP functions `mleGPsep` and `jmleGPsep`, and then confirmed by a large scale crossvalidation. This crossvalidation yields robust and reproducible results and the analysis in this paper therefore relies on the kernel settings estimated through it. See the following table for the most important values used, or Figure S2 for a summary of all estimated parameters for each ancestry component.

| Multivariate method | Dimension | $\sqrt{\theta_x}$ | $\sqrt{\theta_y}$ | $\sqrt{\theta_t}$ | $\eta$ |
|---|---|---|---|---|---|
| MDS2 | C1 | 900 | 900 | 800 | 0.0710309 |
| MDS2 | C2 | 900 | 900 | 600 | 0.0589138 |
| PCA5 | C1 | 1000 | 1000 | 700 | 0.0790766 |
| PCA5 | C2 | 800 | 800 | 800 | 0.0806609 |
| PCA5 | C3 | 900 | 900 | 800 | 0.1412002 |
| PCA5 | C4 | 700 | 700 | 700 | 0.4677798 |
| PCA5 | C5 | 900 | 900 | 800 | 0.3623957 |

Beyond that we also experimented with smaller kernels and kernels with $\theta_s \ll \theta_t$ and $\theta_s \gg \theta_t$. Above results indicate that a rather large range of covariance functions may yield satisfying models, and for the mobility estimation attempted here, smaller kernels may theoretically be more useful. They could produce stronger and more sharply bounded signals for specific events of change. A kernel with a high $\theta_s$ and $\theta_t$ on the other hand may obscure phenomena of temporal change by smoothing them out and by artificially attributing them an earlier starting and later end time. In the end, though, our experiments left us to believe that the different plausible kernel choices yield rather similar patterns for the mobility estimation and we focused on only one, numerically optimal setting.

# 3 Supplementary Text: The similarity search algorithm

The main question for this paper was to estimate and quantify genetic spatiotemporal similarity and therefore ancestry relocation through human mobility. We assume this can in principle be done because people carry their genetic ancestry profiles with them when they move. Mobility estimation requires i) a suitable dimension reduction for "ancestry", ii) a handle on the sparseness of genetic data and iii) an algorithm to derive a probabilistic measure of genetic similarity for individual samples through space and time, which considers aforementioned sparsity. We finally need iiii), a method to assess the similarity space to quantify meaningful signals of possible mobility.

We deal with the first requirement with different multivariate methods, which assign every individual two or more principal components (see Supp. Text 1). For simplicity, in the following sections we will only assume a single principal component, called $C$. The second requirement can be solved by interpolation through Gaussian process regression, as implemented in the laGP R package [8]. With a suitable kernel (see Supp. Text 2), this yields an estimate of the genetic ancestry component $C$ as a *smooth* function in space and time. "Smooth" here means that our function $C$ is continuous and differentiable within the focus area and focus time. Our solutions for the third and fourth requirement will be explained below.

## 3.1 Ancestry and sample-wise similarity fields

Consider a genetic component $C$ as a function of a 2D spatial position $x$ and $y$ and time $t$. Then, our genetic component is a function $C(x, y, t)$, like for example a temperature "field". Keeping $y$ and $t$ fixed, along $x$ the theoretical field and the samples from which it is derived may look like Figure S27 **A**. In this example, the genetic component $C$ follows a gradient with lower values on the left side (say, "West") and higher values on the right side (say, "East"). Thanks to our probabilistic interpolation method, each point of our field has an uncertainty reflecting the heterogeneity and sparsity of observations around it, which we abbreviate $\sigma(x, y, t)$. In practice, we consider a finite number of positions for which we determine the interpolation mean $C$ and variance $\sigma$ (Figure S27 **B**).

Now, consider a focal archaeogenetic sample $S_{x,y,t}$ with ancestry component $C_s$, a measurement of the ancestry component at a given point in space and time (Figure S27 **C**). We can write down the conditional probability that our individual with ancestry $C_s$ matches our ancestry field $C$ at location $(x, y, t)$ using the normal distribution (for brevity we hide $t$):

$$p(C_s|x, y) = \mathcal{N}(C(x, y), \sigma(x, y)) = \frac{1}{\sigma(x, y)\sqrt{2\pi}} e^{-\frac{1}{2}\left(\frac{C_s - C(x,y)}{\sigma(x,y)}\right)^2}$$

From a Bayesian perspective, this is the conditional probability of observing data $C_s$ under a model $(x, y)$, where "model" here simply refers to an unobserved similarity point. Such a conditional probability, where the data appears left and the model parameters right is a likelihood. We can flip this using Bayes' formula to obtain the posterior probability for $(x, y)$ given data $C_s$, using a prior probability $p(x, y)$:

$$p(x, y|C_s) = \frac{p(C_s|x, y)p(x, y)}{Z(C_s)}$$

where $Z$ simply is the normalization constant that makes this expression a valid probability over $x, y$:

$$Z(C_s) = \int_x \int_y p(x, y|C_s) \mathrm{d}x \mathrm{d}y$$

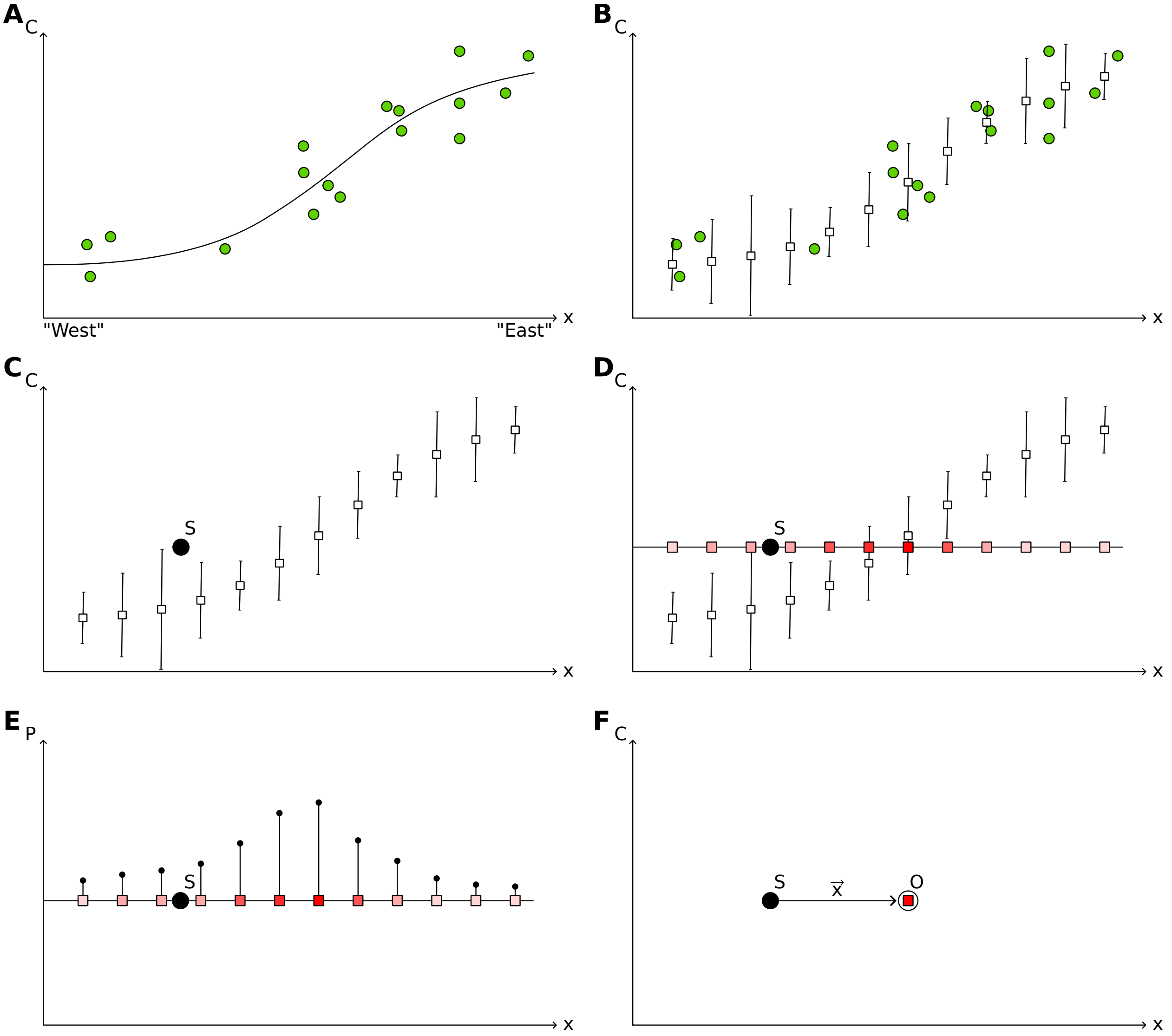


**Figure S27**: Schemata to explain the similarity search algorithm.

This leads to a posterior ”similarity” probability for potentially any point in space, so a similarity probability field. In our implementation the resolution of this field is limited by the regular, interpolated search grid (Figure S27 **D & E**).

For real-world data this operation requires some further generalisation. Above we had a single component $C(x, y)$, but actually we have multiple, typically at least $C_1(x, y)$ and $C_2(x, y)$. To combine the two, we simply compute probabilities $p_1(x, y|C_{s1})$ based on $C_1(x, y)$ and the focal value $C_{s1}$, and $p_2(x, y|C_{s2})$ based on $C_2(x, y)$ and the focal value $C_{s2}$. To get a combined similarity probability, we multiply the two and normalise again.

Real world data is also not precisely positioned in space and – even more severely – time, e.g. through uncertainties in absolute dating, where most input samples informing the interpolated field are either dated with radiocarbon ages or through archaeological context information. For the former we can derive a complex post-calibration radiocarbon probability distribution, for the latter at least a uniform probability distribution from the potential start to the potential end point. One solution to consider this is through iterations of

random sampling, which leaves us with sampling iterations for $C$ and thus $p(x, y|C_s)$. In this case a combined probability field could be calculated as the mean of the individual fields, but for our large-scale mobility proxy (see below) we in fact computed separate mobility vectors for each temporal resampling iteration.

## 3.2 Diachronic mobility proxy

For derived applications and as a simpler summary statistic we give special consideration to the maximum of the posterior probability at a given time before the age of a sample of interest, so the spatial position of maximum genetic similarity $O_S$ in a past reconstructed similarity field (Figure S27 **F**). If we compute $C$ for a sample $S_{x,y,t}$ not at the time $t$, but for a previous time step $t - u$, then $O_S$ can be considered a measure of a likely point of "origin" for the ancestry profile of $S$ at $t - u$. The vector $\overrightarrow{x_S}$, pointing from $S$ to $O_S$ then becomes a measure of ancestry relocation through time, which is a proxy for mobility: If $\Delta x \approx 0$, then no spatial mobility took place within the time $u$. For $\Delta x \gg 0$ we can assume some relocation. $u$ is a free parameter and we call it the retrospection distance. The vector with length $\Delta x$ we call the mobility vector $\overrightarrow{x_S}$. It has both an informative length/magnitude and a direction. Many mobility vectors $\overrightarrow{x_{S_1}}, ..., \overrightarrow{x_{S_n}}$ from samples $S_1, ..., S_n$ can be spatially and temporally binned to compute regional and diachronic mobility proxies.

## 3.3 Concrete steps for the mobility estimation

For the large-scale mobility estimation performed for this paper, we additionally considered different parameter permutations. Please see the following summary of the concrete steps undertaken. The interpolation and similarity search behind other applications in the paper are considerably more simple and require fewer summary operations.

1. For each individual sample $S(x, y, t)$ we interpolated the ancestry fields $C_1(x, y, t-u), ..., C_n(x, y, t-u)$. This is done for the MDS output dimensions C1 and C2 and for Projection PCA C1-C5 (MDS2 & PCA5, see Supp. Text 1). The spatial target cell-size for the grid is set to 100km and for the retrospection distance $u$ we iterate through three settings (see Supp. Text 5). The interpolation is repeated in 25 temporal resampling iterations and we thus get a total of $7*3*25 = 525$ permutations of the interpolated field.

2. We then calculate the respective 525 similarity probability fields $p(x, y|C_s)$ for each sample.

3. The 2 or 5 probability fields of the individual ancestry components $C_1, ..., C_n$ (2 for MDS and 5 for PCA) are then multiplied respectively to derive $2 * 3 * 25 = 150$ combined fields for each sample, 75 for each multivariate method.

4. For each of these 150 fields and each sample we determine the point of maximum genetic similarity $O_S$ and the mobility vector $\overrightarrow{x_S}$.

5. This leaves us with 150 vectors for each sample. For each of the three retrospection distances $u$ and both multivariate methods, we then combine the 25 temporal resampling iterations to visualize the sample-wise results in the mobility figures (Figure 5 and others) with the following operations:

   (a) The length of the mobility vectors as displayed on the y-axis of Figure 5 is calculated by averaging the 25 distances between sample location and estimated maximum similarity point for the spatial

dimensions $x$ and $y$ separately ($\overline{O_x}$ and $\overline{O_y}$), and then computing the average mobility distance $d = \sqrt{\overline{O_x}^2 + \overline{O_y}^2}$. This type of averaging corresponds to computing a vector-wise mean first, before computing its length.

(b) The error bars of the distances are computed as standard deviations of the lengths of the 25 individual mobility vectors, in order to give an impression of their uncertainty.

(c) The angle displayed on the colour scale is given by $\arctan(\overline{O_y}/\overline{O_x})$.

6. The actual mobility curve, so the grey line and ribbon in the background of Figure 5 is a region-wise, diachronic summary statistic computed for an overlapping sequence of moving time-windows combining the sample-wise mobility vectors as follows:

   (a) In a given region for every 400 year time window (one step every 50 years) we compute the length of the vector-wise average of the previously computed sample-wise mean mobility vectors. In Figure 5 this is shown as a grey line in the background.

   (b) The grey ribbon around that line is defined as $\pm 2$ times the standard error of the mean of the individual sample-wise distances in a given 400 year window.

# 4 Supplementary Text: A toy simulation to demonstrate the similarity search algorithm

To explore the robustness of the search algorithm described in Supp. Text 3 we implemented a minimal simulation study. For the sake of a minimum of complexity and to stay focused on a basic, key question, we decided not to implement a spatiotemporal, agent-based model with artificial genomes or an equivalent substitute, but to only consider the very derived proxy of a position along a genetic component like for example the first MDS output coordinate. We specifically tried to answer the following question with a setup that is as simple as possible both in structure and parameters: *How reliable is the spatial similarity search given varying genetic between-area distances and how do basic context parameters affect it?*

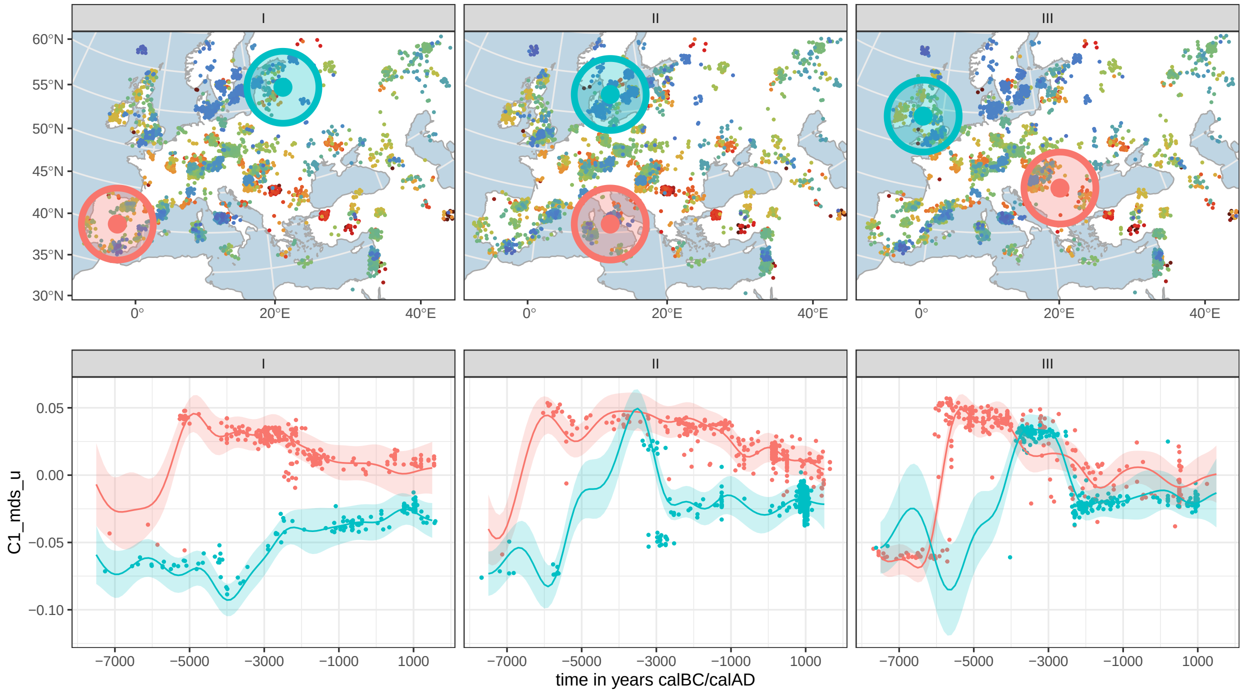


**Figure S28**: Real world examples of the development of C1_mds_u (see Supp. Text 1) through time for six European regions. The three maps on top show the (arbitrary) pairs plotted with a center point and a 500km radius. The bottom plots show C1_mds_u through time, with the individual dots representing samples from the respective 1000km circles. The smooth curves are the output of the default GPR interpolation established for this paper at the spatial center points of the regions. The ribbons around the curves cover one standard deviation. Note that the interpolation is also influenced by samples not in the 1000km circles and thus not plotted in the bottom plots.

To contextualize this question, it is helpful to consider some of the real world examples reconstructed with the data and methodology established for this paper. Ideally a similarity search algorithm should be able to distinguish a minimum of two areas independent of its genomic history through time. Figure S28 shows three pairs of regions and their respective developments along a genetic component C1_mds_u (from now on only called C1) relevant for the analysis in this paper (see Supp. Text 1). The areas undergo different population-level processes, that are indirectly represented by the rough proxy of individual samples on C1. The area-pair in Example I expresses a funnel-like pattern, where the genetic profiles of Iberia and the Eastern Baltic Sea region gradually get more similar over time. Example II contrasts the relative stability

of C1 for Italy since the Neolithic with the rapid changes Southern Scandinavia passes through in the third millennium BC. In Example III, Great Britain and Ireland show a generally similar development as the Balkans, but lag behind on the Early Neolithic population shift (here disregarding the extreme sparsity of pre-Neolithic observations).

We generally assume, that our similarity search algorithm is capable of distinguishing spatial origins, when the spatial distribution of specific genetic components is well accentuated. It should have no problem to attribute samples to either Iberia or the Baltics in the fourth millennium BC. It is less certain, though, how accurate the search would be in case of a higher degree of similarity, like for example between Britain and the Balkans from the fourth millennium onward (only considering C1!).

## 4.1 Simulation setup

For the simulation we assume a world with two spatial ($x$, $y$) and one temporal dimension ($z$). Each of these three dimensions scales between 0 and 1 and the space is fully homogeneous and featureless. Within this world, observations (”samples”) are randomly distributed following a uniform distribution through space and time. The sample size is a variable parameter of this setup to later measure the effect of sample sparsity. One quarter of the spatial square landscape passes through a different genetic development as the rest (see Figure S29), which puts it more or less apart through time. As an analogy to the real-world C1_mds_u, the genetic component is represented by a numeric value roughly scaling between 0 and 1.

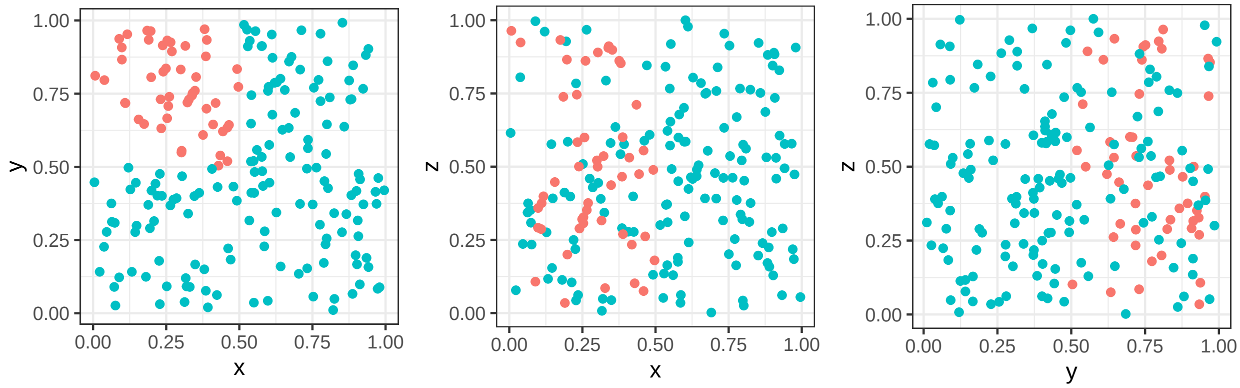


**Figure S29**: One iteration of the simulated sample distribution with $4 \times 50 = 200$ samples. The three plots show the spatiotemporal 3D cube world from different perspectives, so the first plot can be understood as a spatial map. The colors serve to distinguish the two focal areas with divergent genetic developments.

For the population-wise development of this ancestry proxy we consider three scenarios, that are inspired by the real-world observations in Figure S28: A scenario *linear*, where the two spatial areas are genetically different at the beginning (so at $z = 0$), but become more similar over time in a process of linear growth, a scenario *limited* with the same outset, but a faster synchronization through limited growth (reaching almost full identity at $z = 1$), and finally a scenario *intertwined*, where the similarity in ancestry increases in an oscillating pattern (Figure S30 **A**). Each sample $S_{x,y,z}$ gets a genetic component $C$ according to these time-dependent scenarios. The ”simulated” observation is sampled from a normal distribution with a fixed standard deviation of 0.1 and whose mean is defined by the respective scenario.

$$C(S_{x,y,z}) \sim \mathcal{N}(\text{scenario}(z),\, 0.1)$$

We rerun this whole system across the three different scenarios (*linear*, *limited* and *intertwined*), three different sample sizes ($4 \times 10 = 40$, $4 \times 50 = 200$ and $4 \times 100 = 400$) and 100 resampling iterations, where both the spatiotemporal positions and the genetic values of the samples vary according to the aforementioned priors.

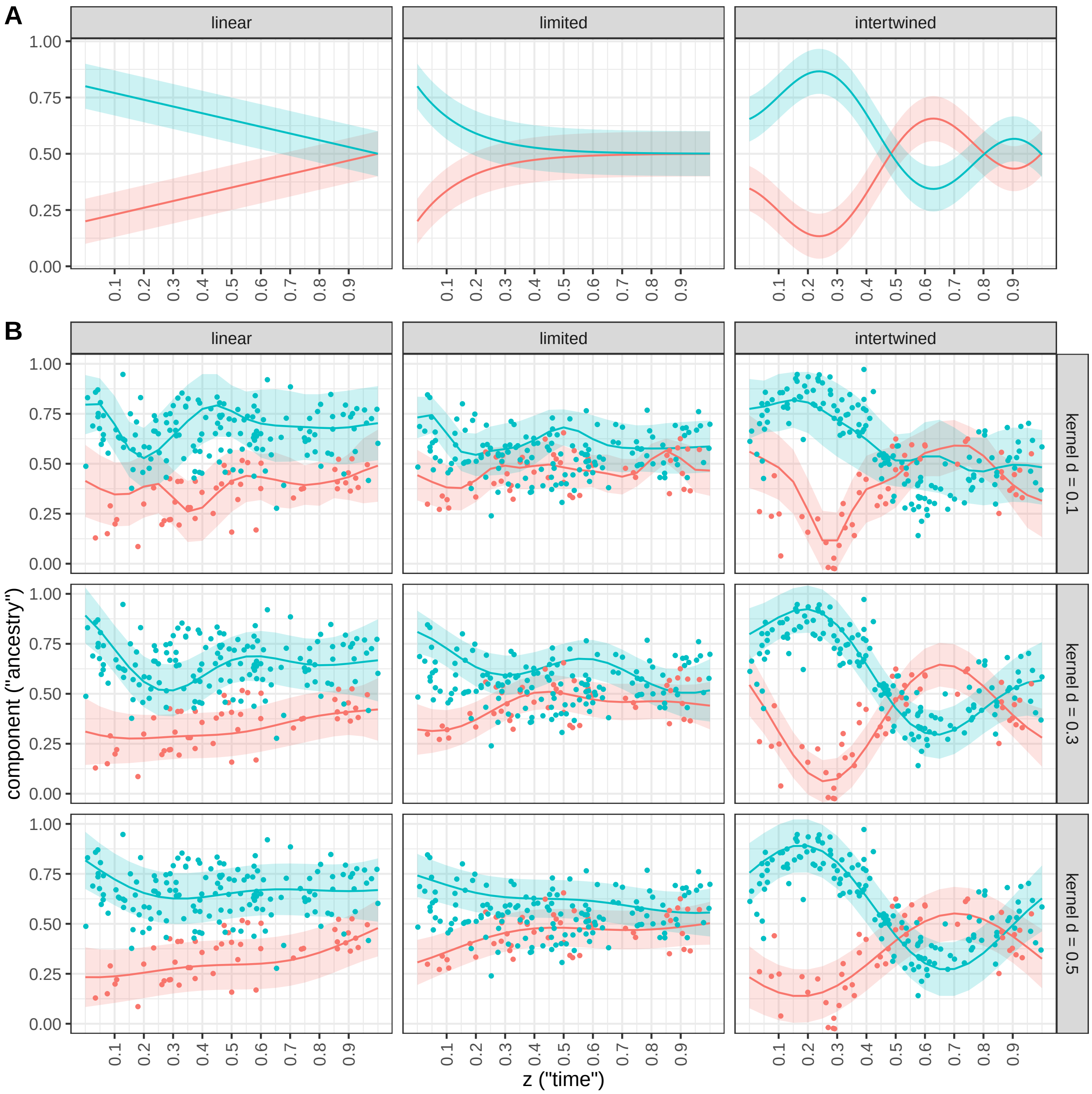


**Figure S30**: "Genetic" development through time for the artificial simulation scenarios. **A** shows the three theoretical models, **B** randomly sampled iterations of these scenarios with $4 \times 50 = 200$ samples. The fitted curves are created via the GPR interpolation (just as in Figure S28) at the spatial positions $[0.25, 0.75]$ and $[0.75, 0.25]$. The ribbons show one standard deviation of the field.

## 4.2 Interpolation

Figure S30 **B** shows one resampling iteration for the sample size $4 \times 50 = 200$. The randomly drawn points behave according to the input scenarios on the x-axis of the plot matrix. There are three times more blue points as there are red points following the spatial setup introduced above (Figure S29), so the red point cloud is naturally more sparse.

To get a better understanding of the Gaussian process regression algorithm employed in this paper and to evaluate how well the reconstructed ancestry field captures the respective input scenarios for the pseudo-"genetic" component in this simulation, we ran the interpolation for two spatial points within the red and the blue area through time and projected mean and standard deviation on to the respective point clouds in Figure S30 **B**. The positions of these points are $[0.25, 0.75]$ for the red area and $[0.75, 0.25]$ for the larger blue one (see Figure S29 for reference). So the fairly irregular curves we see in the plot only represent one central point within the distinguished areas and are fully dependent on the noisy samples informing the field around them through time. They do not capture the input scenarios perfectly and are accurate only to the degree the interpolated field does so at this one spatial position. Besides the available input samples, the quality of this reconstruction also depends to on the parameters set for the field, namely the characteristics of the covariance function (kernel). Here we set the nugget term to $\eta = 0.1$ (considering the deviation we set for the sampling process generating this artificial data) and varied the lengthscale parameter in three different permutations (equal for both spatial and the temporal dimension).

Unsurprisingly we observe that smaller kernels yield more irregular, larger ones more smooth curves, but generally the field succeeds in reconstructing the broad strokes of the input scenarios.

## 4.3 Similarity search

As explained in Supp. Text 3, our similarity search relies on the interpolation of ancestry components and the search for points of maximum similarity in time slices of the "genetic" field. To measure the accuracy of the similarity search algorithm in the described simulation setup we constructed the following test: We assume a search sample $S_z$ with one (!) genetic component

$$C(S_z) = \text{scenario}(z)$$

for a sequence of $z = 0.1, 0.2, \ldots, 0.9$. We omitted $x$ and $y$ here, because they are without effect for the similarity search in this application (they would only matter if we were to assign a similarity vector), but for the sake of tangibility we imagine this sample to represent an individual who left the red area and is now found among the blue samples at $[0.75, 0.25]$. We now use the similarity search to determine the point of highest, interpolated similarity of the field in the same time slices $z$, given the different system parameter permutations.

Figure S31 shows one run of this experiment for a sample size of $4 \times 50 = 200$, a kernel size of $d = 0.3$ and one arbitrary resampling iteration. For each ancestry scenario nine subplots show the search outcomes for different time slices. The orange dot indicates the maximum similarity point, so the derived result of the search. Ideally we want this point to be always in the top left quarter of the search map: If it is there, then the search yielded an accurate result. Despite the fact we are only considering a single ancestry component here, for the *linear* scenario this is mostly the case through time, although the light blue areas of the similarity probability rasters show how the distinction of the red and blue areas slowly fall apart later on (see e.g. the field for $z = 0.9$), as the two populations become less well distinguished by ancestry $C$. This

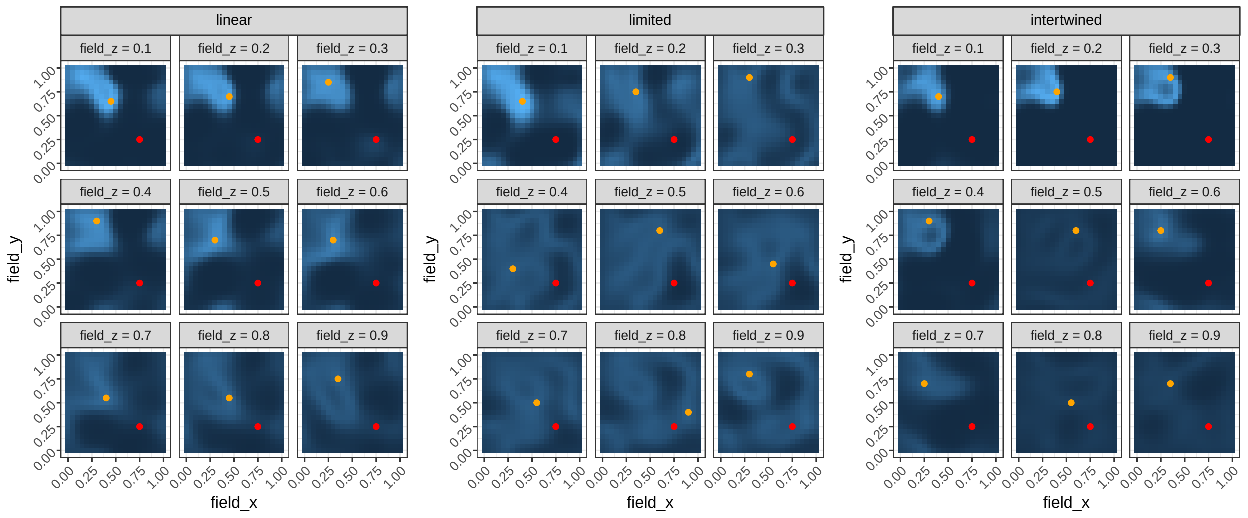


**Figure S31**: One iteration of the simulated similarity search through time for the three different simulated scenarios. Each search time slice (field_z) is reflected by one subplot in a matrix of nine plots for each scenario. The blue raster maps in the background of each of these plots show the similarity probability for each pixel, with dark blue representing lower and light blue higher probabilities. The maximum likelihood point is indicated with an orange dot. The red dot marks the (in fact irrelevant) position of the search sample in the imaginary scenario underlying this figure.

process of deterioration is quicker and more severe for the more challenging scenario *limited*. Here, the correct attribution fails already for $z = 0.4$, with a high potential similarity probability almost everywhere in the square simulation world. For the more complex scenario *intertwined*, finally, the similarity search reflects the volatile development of the ancestry component (see Figure S30 **A**): For $z \leq 0.4$ the model is well able to distinguish the areas, for $z = 0.5$ it fails, for $z = 0.6$ & $z = 0.7$ it works again to then finally fall apart for $z \geq 0.8$.

The example in Figure S31 is informative, but as a single iteration not conclusive on the behaviour of the search in the above established simulation setup. For that we have to consider more parameter permutations and a representative number of resampling iterations. Figure S32 summarises runs across three different samples sizes, three different ancestry scenarios and three different interpolation kernel sizes. Each of these combinations is resampled 100 times. For all $3 * 3 * 3 * 100 = 2700$ permutations we run the similarity search for the nine time slices and check for each of them, if the result point is within the spatial top left ("red") quadrant of the simulation world. If this is the case, we count this run towards the *number of "correct" maximum similarity attributions* as plotted on the y-axis. of Figure S31. If this number reaches 100, so includes every single one of the resampling iterations, then the search algorithm managed to detect the correct origin area in 100% of the random spatiotemporal sample distributions. If this proxy goes down to 25, then it does not perform better than a random coordinate generator, which would place approximately 25 of 100 runs in the correct quarter of the simulation world.

A closer look at this figure reveals a number of important observations: The three different ancestry scenarios yield vastly different results. *linear* is simple and keeps the two areas apart just until the very end. Irrespective of sample- or kernel size the similarity search accuracy is high. It is slightly lower, though, at the end of the time sequence (so when the two areas are becoming more similar, see Figure S30), if either the sample or the kernel size is too small. For the *limited* scenario the observed accuracy is much

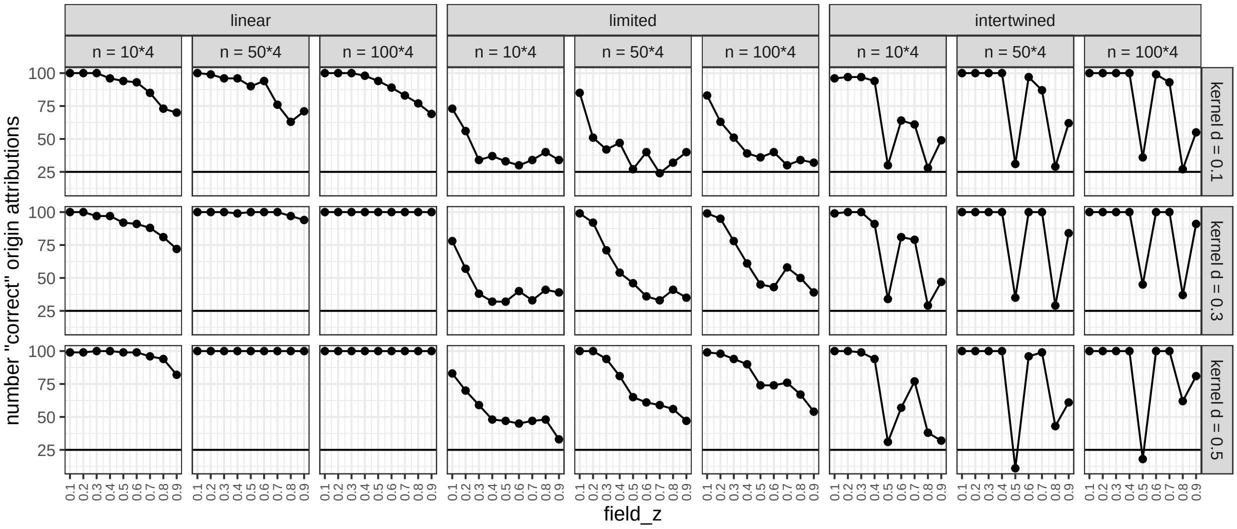


**Figure S32**: Results of the permutation analysis for similarity search accuracy given different parameter settings. Each of the 27 subplots for one of the three scenarios, one of the three population sizes and one of the three kernel sizes, summarises 900 runs of the search in the artificial simulation setup: 100 for each of the 9 time slices. The time slices are distinguished on the x-axis, and the y-axis thus encodes how many of the 100 searches per slice yielded an accurate search result. The horizontal line at $y = 25$ is the random-attribution baseline, which hints at a fully failing similarity search if undercut.

lower. Especially for very low sample sizes a distinction of the two source areas is barely possible for later time slices. Increasing the kernel size helps to smooth out sampling gaps and keeps the accuracy above the random-threshold. The *intertwined* scenario highlights how quickly the similarity search can fall apart, but also recover again in case of opposing and overlapping genetic developments for the two focal areas. It fails dramatically for $z = 0.5$ no matter the model parameter settings.

## 4.4 Conclusion

The purpose of this simulation exercise was to get a better understanding the robustness of the similarity search algorithm in different scenarios. From our analysis we conclude, that said robustness is high and should generally be suitable for real-world data on the orders of magnitude relevant for this paper. There are, of course scenarios, where the algorithm fails to yield meaningful output. These are notably significant bi-directional genetic turnover, so when two regions swap their genetic make-up, and extensive cross-regional synchronization. The former can be considered unlikely or at least rare, but the latter is a defining property of the Western Eurasian archaeogenetic record for various regions after the Bronze Age, which happens to be a focal research context for this study.

We considered this setup in the artificial *limited* scenario of this simulation and learned that large sample sizes and large kernel sizes improve the accuracy of the search considerably. Larger kernels allow the past to inform the present, which is a reasonably safe default for the similarity search application. For the large scale mobility estimation attempted in this study, we even emphasised this effect through the introduction of the retrospection distance parameter.

We finally highlight two more features of our similarity search algorithm that mitigate undesired effects: 1.) By picking not just one, but two or more ancestry components, we render it less likely that two regions

become fully similar in their ancestry profile. Two or more dimensions are less likely to be spuriously similar. 2.) Our algorithm is probabilistic, and reveals for each sample a probability distribution through space, which captures the full uncertainty of our search. So in cases of ambiguity, we expect the probability distribution to reflect this ambiguity and make it transparent to a user of our method.

# 5 Supplementary Text: Mobility curve exploration

## 5.1 Two additional regions: Southeastern Europe and Western Pontic Steppe

For conciseness we decided to only discuss four mobility analysis regions in the main text: Britain and Ireland, Central Europe, Iberia and Italy. We ran the mobility estimation for all samples, though, and defined two additional regions to be considered here now: Southeastern Europe and the Western Pontic Steppe (see Figure S10).

Southeastern Europe stands out in our analysis, because we could include a high number of comparatively early samples from the Mesolithic, e.g. M96 [11]. All of them are from the small and extraordinary Iron Gates area in Serbia and Romania, where the Danube passes between the Balkan Mountains and the Southern Carpathian Mountains range. During the 6th millennium BC, correlating well with the beginning of the Neolithisation in the region [12], we observe the emergence of non-locality signals: The ancestry profile of Early Neolithic individuals like e.g. I3948 [13] from the Adriatic coast points to Western Anatolia. Surprisingly, for the individual I2534 [13, 14], we observe a long mobility vector to the North, even after the onset of the Neolithic. This individual might not necessarily have been (personally or through their immediate ancestors) part of any permanent long-range mobility: they lived at a time and place where new ancestry was arriving with the Neolithic package – rapidly changing the local ancestry landscape – and their genetic "displacement" thus becomes an indirect proxy of the major mobility event surrounding them. The unexpected peak with northwestern direction in the 5th millennium is carried by chalcolithic individuals from Bulgaria (all from ref. [13]), whose mobility vectors point to Central Europe. Unlike other European regions, the arrival of Steppe ancestry in Southeastern Europe is more gradual, beginning earlier and less abruptly [13]. Few individuals show a clear mobility signal pointing to the far Northeast – e.g. I4175 [13]. For later periods, finally, we observe some remarkable outliers with strong mobility signals: For example the Hungarian Bell Beaker I2787 [15], the Iron Age Scythian DA197 [16] or the Migration Period Hunnic individual HUN001 [17].

Even further to the East, in the Western Pontic steppe (including the area north of the Greater Caucasus mountain range), we see a quite varied account of ancestry influx. For the Ukrainian samples from the sixth millennium and before, Mathieson et al. 2018 [13] report ancestry on a cline between Eastern-, Scandinavian Mesolithic- and later Western Hunter-Gatherers. This genetic affinity is reflected in the first increase of signal we observe mainly from the Northwest during the sixth millennium, confirming previously described similarities in the developments in Eastern and Northeastern Europe [18]. Only at the beginning of the fifth millennium one extraordinary individual (I3719) stands out with "entirely northwestern-Anatolian-Neolithic-related ancestry" [13] and thus long-distance affinity to the West and Southwest. Most data for the Neolithic and the Bronze Age is from the Caucasus region and documents a complex, though relatively local mobility history [19]. Within this time frame, multiple Globular Amphora context individuals (e.g. ILK003 [13]) from present-day Ukraine show a strong mobility signal from the West. During the Iron Age, more individuals with a relatively long-distance mobility signal appear, for example cim359 [20] and MJ-13 [21]. Their mobility vectors point to the opposite ends of Europe, possibly illustrating the region's position as a bridge between Europe and Central Eurasia, housing different equestrian steppe nomad populations – e.g. Cimmerians, Scythians, and Sarmatians. This generally holds true into historical times, including the Migration- [16] and Medieval Periods (e.g. VK542 [5]).

## 5.2 Comparing different mobility curves

The mobility estimation presented in this paper depends on a large number of parameters. For many of them there is no naturally optimal choice, so we had to make multiple intuitive or empirically informed decisions. Supp. Text 1 explains how we selected the simplified genetic space to interpolate for our ancestry field and how we settled on a 2D MDS and a 5D projection-based PCA. Supp. Text 2 explains how we determined the parameters for the Gaussian process regression interpolation. As explained in Supp. Text 3, another key parameter arises from the retrospection distance that should be used for the similarity search algorithm in our large scale mobility estimation.

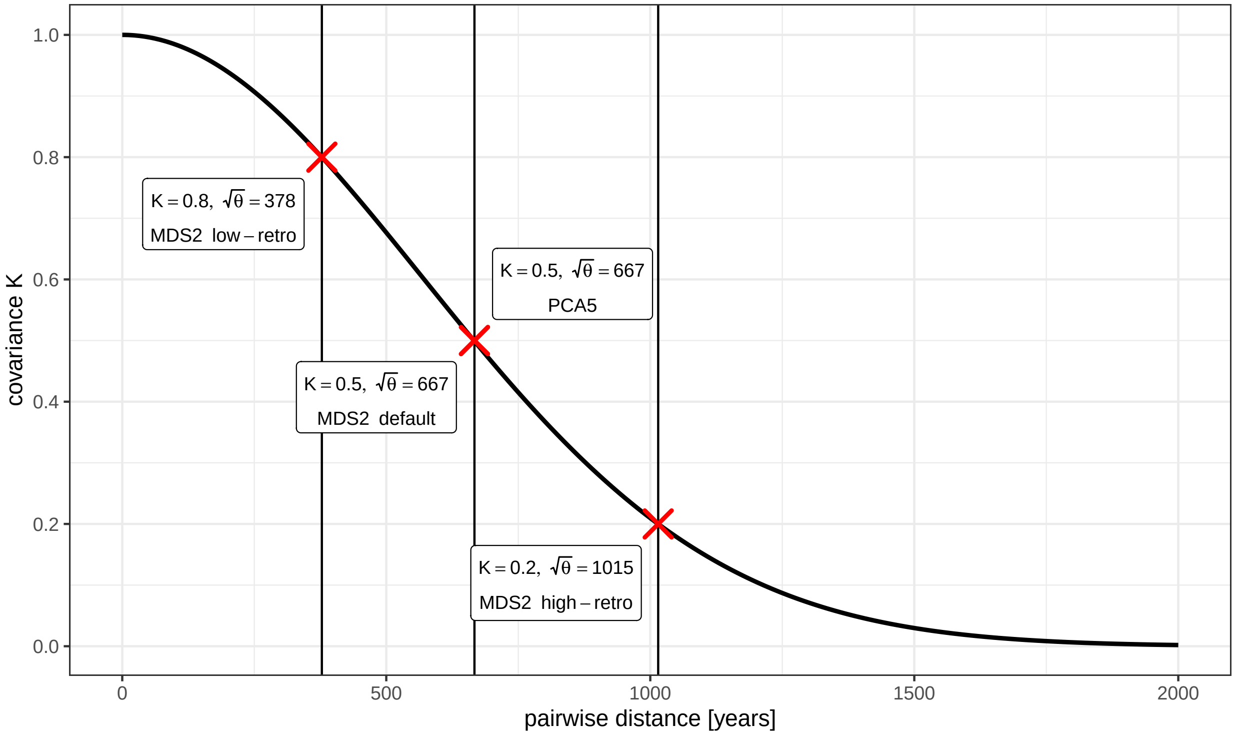


**Figure S33**: Retrospection distance settings for the different runs.

Figure S33 shows that we eventually decided to consider three values for the retrospection distance, informed by an assumed temporal kernel size of $\sqrt{\theta_t} = 800$: An intermediate one based on the point of $Cov(x, x') = 0.5$ (*default*), a low one at $Cov(x, x') = 0.8$ (*low*) and a high one at $Cov(x, x') = 0.2$ (*high*). For each of these settings we reran the mobility estimation and produced the curve plots Figure S10, S12 and S13. An additional version in Figure S11 shows the PCA5 result, again with the *default* retrospection distance. Figure S34 is finally an attempt to visualise the major differences between these iterations.

The curves for all three experimental settings (Figure S11, S12, S13) are generally similar to the default (S10). The main peaks and depressions generally overlap and seem to be detected robustly. A number of differences emerge, though: A lower retrospection distance generally causes shorter mean mobility vectors, whereas a higher one causes the peak similarity to be further away. This is a strong signal, but not surprising: It is plausible that the further one goes back in time, the further away ones ancestors might have lived originally. Especially the main long-distance events during the Early and Late Neolithic get exaggerated by this effect (see e.g. the timeseries for the Stuttgart sample in Figure S7) and only few contexts and individuals

seem to deviate from this general pattern entirely.

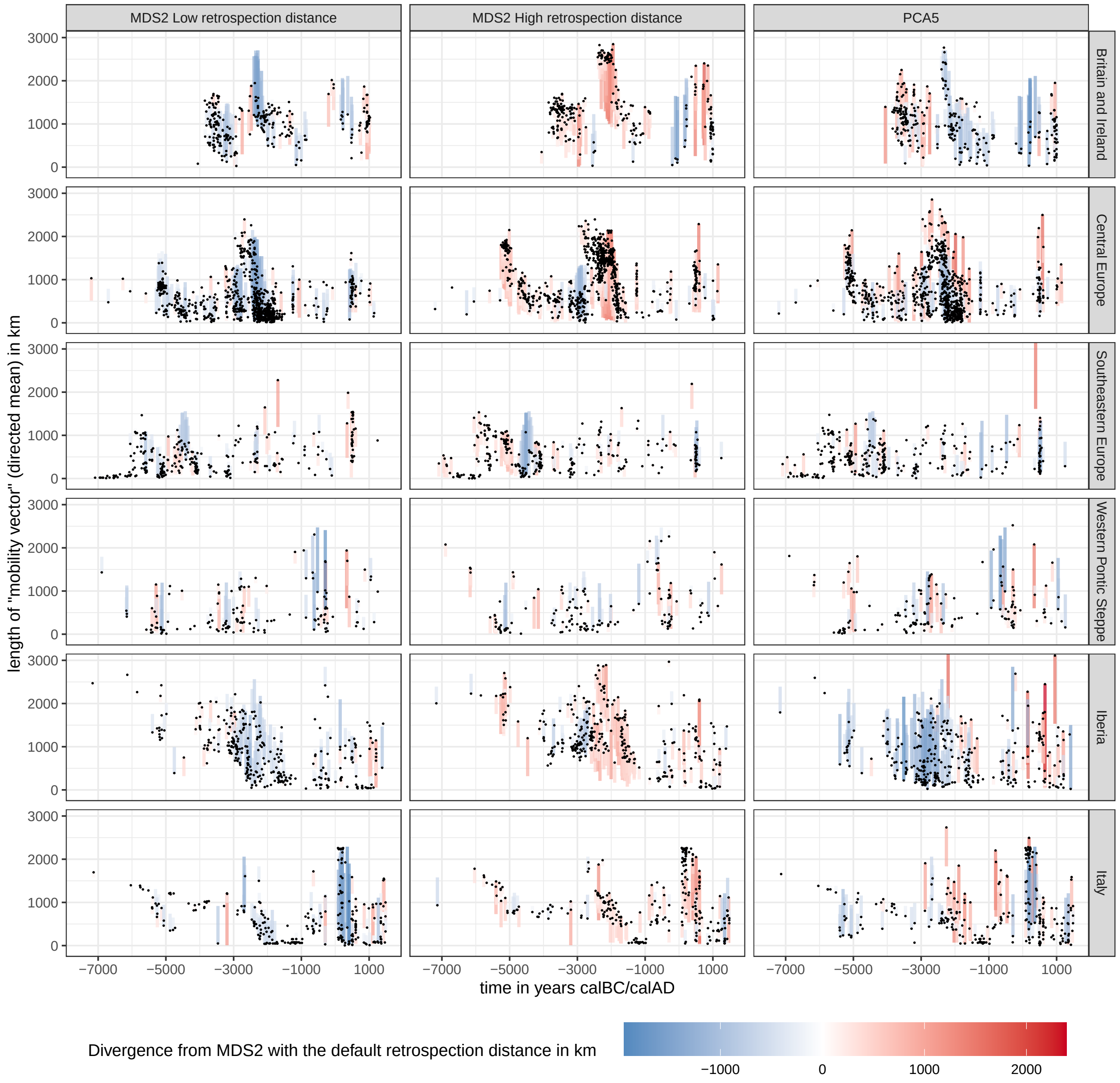


**Figure S34**: Comparison of different mobility estimation runs in a plot matrix. Each row of the plot matrix covers one analysis region, each column one of the three additional run configurations. The dots show the length of the summarized mobility vectors of one sample, just as in Figure 5. Vertical lines connect the results for the given run and the MDS2 run with default retrospection distance as in Figure 5 and Figure S10. The diverging colour scheme of these lines highlight when, where and to which degree the runs yield different mean mobility vector lengths.

The difference between the MDS2 and the PCA5 run are less systematic. For various regions and time periods, e.g. Britain and Ireland before 3000BC, Central Europe in the Late Neolithic and the Early Bronze Age, Iberia after AD and Italy between 2500 and 500BC, the PCA5-based search finds some markedly longer vectors. For Britain and Ireland after 3000BC and most notably Iberia before 2500BC the opposite is the case. A look at the outlier or peak-mobility individuals highlighted in the main text and above points towards

more conservative estimates for the PCA5 run: Outliers in the MDS2 run with default retrospection distance usually also emerge as outliers when the retrospection distance is modified, but often don't with PCA5. This applies especially for individuals from the Western Pontic Steppe, Iberia and Italy.

# C Bibliography: Supplementary Texts


[1] Ludovic Orlando et al. "Ancient DNA analysis". In: *Nature Reviews Methods Primers* 1.1 (Feb. 2021). DOI: 10.1038/s43586-020-00011-0.

[2] Shaun Purcell et al. "PLINK: a tool set for whole-genome association and population-based linkage analyses". In: *Am. J. Hum. Genet.* 81.3 (Sept. 2007), pp. 559–575.

[3] Salvador Herrando-Pérez, Raymond Tobler, and Christian D. Huber. "smartsnp, an R package for fast multivariate analyses of big genomic data". In: *Methods in Ecology and Evolution* 12.11 (Aug. 2021), pp. 2084–2093. DOI: 10.1111/2041-210x.13684.

[4] Jonas Meisner et al. "Large-scale inference of population structure in presence of missingness using PCA". In: *Bioinformatics* 37.13 (Jan. 2021), pp. 1868–1875. DOI: 10.1093/bioinformatics/btab027.

[5] Ashot Margaryan et al. "Population genomics of the Viking world". In: *Nature* 585.7825 (Sept. 2020), pp. 390–396. DOI: 10.1038/s41586-020-2688-8.

[6] Alkes L. Price et al. "Long-Range LD can confound genome scans in admixed populations". In: *The American Journal of Human Genetics* 83.1 (2008), pp. 132–135. DOI: 10.1016/j.ajhg.2008.06.005.

[7] Carl A Anderson et al. "Data quality control in genetic case-control association studies". In: *Nat. Protoc.* 5.9 (Aug. 2010), pp. 1564–1573. DOI: 10.1038/nprot.2010.116.

[8] Robert B. Gramacy. "laGP: Large-scale spatial modeling via Local Approximate Gaussian Processes in R". In: *Journal of Statistical Software* 72.1 (2016), pp. 1–46. DOI: 10.18637/jss.v072.i01.

[9] Fernando Racimo et al. "The spatiotemporal spread of human migrations during the European Holocene". en. In: *Proc. Natl. Acad. Sci. U. S. A.* 117.16 (Apr. 2020), pp. 8989–9000. DOI: 10.1073/pnas.1920051117.

[10] S Banerjee, B P Carlin, and A E Gelfand. *Hierarchical modeling and analysis for spatial data. 2nd Edition.* Boca Raton, FL: CRC Press, 2015.

[11] Gloria González-Fortes et al. "Paleogenomic Evidence for Multi-generational Mixing between Neolithic Farmers and Mesolithic Hunter-Gatherers in the Lower Danube Basin". In: *Current Biology* 27.12 (June 2017), 1801–1810.e10. DOI: 10.1016/j.cub.2017.05.023.

[12] Marko Porčić et al. "The timing and tempo of the Neolithic expansion across the Central Balkans in the light of the new radiocarbon evidence". In: *Journal of Archaeological Science: Reports* 33 (Oct. 2020), p. 102528. DOI: 10.1016/j.jasrep.2020.102528.

[13] Iain Mathieson et al. "The genomic history of Southeastern Europe". In: *Nature* 555.7695 (Mar. 2018), pp. 197–203. DOI: 10.1038/nature25778.

[14] Mark Lipson et al. "Parallel palaeogenomic transects reveal complex genetic history of early European farmers". In: *Nature* 551.7680 (Nov. 2017), pp. 368–372. DOI: 10.1038/nature24476.

[15] Iñigo Olalde et al. "The Beaker phenomenon and the genomic transformation of Northwest Europe". In: *Nature* 555.7695 (Mar. 2018), pp. 190–196. DOI: 10.1038/nature25738.

[16] Peter de Barros Damgaard et al. "137 ancient human genomes from across the Eurasian steppes". In: *Nature* 557.7705 (May 2018), pp. 369–374. DOI: 10.1038/s41586-018-0094-2.

[17] Guido Alberto Gnecchi-Ruscone et al. “Ancient genomic time transect from the Central Asian Steppe unravels the history of the Scythians”. In: *Science Advances* 7.13 (Mar. 2021), eabe4414. DOI: 10.1126/sciadv.abe4414.

[18] Eppie R Jones et al. “The Neolithic transition in the Baltic was not driven by admixture with early European farmers”. en. In: *Curr. Biol.* 27.4 (Feb. 2017), pp. 576–582. DOI: 10.1016/j.cub.2016.12.060.

[19] Chuan-Chao Wang et al. “Ancient human genome-wide data from a 3000-year interval in the Caucasus corresponds with eco-geographic regions”. In: *Nat. Commun.* 10.1 (Feb. 2019), p. 590. DOI: 10.1038/s41467-018-08220-8.

[20] Maja Krzewińska et al. “Ancient genomes suggest the eastern Pontic-Caspian steppe as the source of western Iron Age nomads”. In: *Science Advances* 4.10 (Oct. 2018), eaat4457. DOI: 10.1126/sciadv.aat4457.

[21] Mari Järve et al. “Shifts in the genetic landscape of the Western Eurasian steppe associated with the beginning and end of the Scythian dominance”. In: *Curr. Biol.* 29.14 (July 2019), 2430–2441.e10. DOI: 10.1016/j.cub.2019.06.019.

# D Bibliography: AADR Dataset


[1] Cristina Gamba et al. "Genome flux and stasis in a five millennium transect of European prehistory". In: *Nat Commun* 5.1 (Oct. 2014). DOI: 10.1038/ncomms6257.

[2] Iñigo Olalde et al. "Derived immune and ancestral pigmentation alleles in a 7,000-year-old Mesolithic European". In: *Nature* 507.7491 (Jan. 2014), pp. 225–228. DOI: 10.1038/nature12960.

[3] Iosif Lazaridis et al. "Ancient human genomes suggest three ancestral populations for present-day Europeans". In: *Nature* 513.7518 (Sept. 2014), pp. 409–413. DOI: 10.1038/nature13673.

[4] Pontus Skoglund et al. "Genomic Diversity and Admixture Differs for Stone-Age Scandinavian Foragers and Farmers". In: *Science* 344.6185 (May 2014), pp. 747–750. DOI: 10.1126/science.1253448.

[5] Eppie R. Jones et al. "Upper Palaeolithic genomes reveal deep roots of modern Eurasians". In: *Nat Commun* 6.1 (Nov. 2015). DOI: 10.1038/ncomms9912.

[6] Iñigo Olalde et al. "A Common Genetic Origin for Early Farmers from Mediterranean Cardial and Central European LBK Cultures". In: *Mol Biol Evol* (Sept. 2015), msv181. DOI: 10.1093/molbev/msv181.

[7] Iain Mathieson et al. "Genome-wide patterns of selection in 230 ancient Eurasians". In: *Nature* 528.7583 (Nov. 2015), pp. 499–503. DOI: 10.1038/nature16152.

[8] Lara M. Cassidy et al. "Neolithic and Bronze Age migration to Ireland and establishment of the insular Atlantic genome". In: *Proc. Natl. Acad. Sci. U.S.A.* 113.2 (Dec. 2015), pp. 368–373. DOI: 10.1073/pnas.1518445113.

[9] Morten E. Allentoft et al. "Population genomics of Bronze Age Eurasia". In: *Nature* 522.7555 (June 2015), pp. 167–172. DOI: 10.1038/nature14507.

[10] Torsten Günther et al. "Ancient genomes link early farmers from Atapuerca in Spain to modern-day Basques". In: *Proc. Natl. Acad. Sci. U.S.A.* 112.38 (Sept. 2015), pp. 11917–11922. DOI: 10.1073/pnas.1509851112.

[11] Wolfgang Haak et al. "Massive migration from the steppe was a source for Indo-European languages in Europe". In: *Nature* 522.7555 (Mar. 2015), pp. 207–211. DOI: 10.1038/nature14317.

[12] Ayça Omrak et al. "Genomic Evidence Establishes Anatolia as the Source of the European Neolithic Gene Pool". In: *Current Biology* 26.2 (Jan. 2016), pp. 270–275. DOI: 10.1016/j.cub.2015.12.019.

[13] Farnaz Broushaki et al. "Early Neolithic genomes from the eastern Fertile Crescent". In: *Science* 353.6298 (July 2016), pp. 499–503. DOI: 10.1126/science.aaf7943.

[14] Gülşah Merve Kılınç et al. "The Demographic Development of the First Farmers in Anatolia". In: *Current Biology* 26.19 (Oct. 2016), pp. 2659–2666. DOI: 10.1016/j.cub.2016.07.057.

[15] Iosif Lazaridis et al. "Genomic insights into the origin of farming in the ancient Near East". In: *Nature* 536.7617 (July 2016), pp. 419–424. DOI: 10.1038/nature19310.

[16] Qiaomei Fu et al. "The genetic history of Ice Age Europe". In: *Nature* 534.7606 (May 2016), pp. 200–205. DOI: 10.1038/nature17993.

[17] Rui Martiniano et al. "Genomic signals of migration and continuity in Britain before the Anglo-Saxons". In: *Nat Commun* 7.1 (Jan. 2016). DOI: 10.1038/ncomms10326.

[18] Stephan Schiffels et al. "Iron Age and Anglo-Saxon genomes from East England reveal British migration history". In: *Nat Commun* 7.1 (Jan. 2016). DOI: 10.1038/ncomms10408.

[19] Zuzana Hofmanová et al. "Early farmers from across Europe directly descended from Neolithic Aegeans". In: *Proc. Natl. Acad. Sci. U.S.A.* 113.25 (June 2016), pp. 6886–6891. DOI: 10.1073/pnas.1523951113.

[20] Edwin C. M. van den Brink et al. "A Late Bronze Age II clay coffin from Tel Shaddud in the Central Jezreel Valley, Israel: context and historical implications". In: *Levant* 49.2 (May 2017), pp. 105–135. DOI: 10.1080/00758914.2017.1368204.

[21] Eppie R. Jones et al. "The Neolithic Transition in the Baltic Was Not Driven by Admixture with Early European Farmers". In: *Current Biology* 27.4 (Feb. 2017), pp. 576–582. DOI: 10.1016/j.cub.2016.12.060.

[22] Gloria González-Fortes et al. "Paleogenomic Evidence for Multi-generational Mixing between Neolithic Farmers and Mesolithic Hunter-Gatherers in the Lower Danube Basin". In: *Current Biology* 27.12 (June 2017), 1801–1810.e10. DOI: 10.1016/j.cub.2017.05.023.

[23] Iosif Lazaridis et al. "Genetic origins of the Minoans and Mycenaeans". In: *Nature* 548.7666 (Aug. 2017), pp. 214–218. DOI: 10.1038/nature23310.

[24] Lehti Saag et al. "Extensive Farming in Estonia Started through a Sex-Biased Migration from the Steppe". In: *Current Biology* 27.14 (July 2017), 2185–2193.e6. DOI: 10.1016/j.cub.2017.06.022.

[25] Marc Haber et al. "Continuity and Admixture in the Last Five Millennia of Levantine History from Ancient Canaanite and Present-Day Lebanese Genome Sequences". In: *The American Journal of Human Genetics* 101.2 (Aug. 2017), pp. 274–282. DOI: 10.1016/j.ajhg.2017.06.013.

[26] Mark Lipson et al. "Parallel palaeogenomic transects reveal complex genetic history of early European farmers". In: *Nature* 551.7680 (Nov. 2017), pp. 368–372. DOI: 10.1038/nature24476.

[27] Martin Sikora et al. "Ancient genomes show social and reproductive behavior of early Upper Paleolithic foragers". In: *Science* 358.6363 (Oct. 2017), pp. 659–662. DOI: 10.1126/science.aao1807.

[28] Martina Unterländer et al. "Ancestry and demography and descendants of Iron Age nomads of the Eurasian Steppe". In: *Nat Commun* 8.1 (Mar. 2017). DOI: 10.1038/ncomms14615.

[29] Rui Martiniano et al. "The population genomics of archaeological transition in west Iberia: Investigation of ancient substructure using imputation and haplotype-based methods". In: *PLoS Genet* 13.7 (July 2017). Ed. by Anna Di Rienzo, e1006852. DOI: 10.1371/journal.pgen.1006852.

[30] Verena J. Schuenemann et al. "Ancient Egyptian mummy genomes suggest an increase of Sub-Saharan African ancestry in post-Roman periods". In: *Nat Commun* 8.1 (May 2017). DOI: 10.1038/ncomms15694.

[31] Éadaoin Harney et al. "Ancient DNA from Chalcolithic Israel reveals the role of population mixture in cultural transformation". In: *Nat Commun* 9.1 (Aug. 2018). DOI: 10.1038/s41467-018-05649-9.

[32] Alissa Mittnik et al. "The genetic prehistory of the Baltic Sea region". In: *Nat Commun* 9.1 (Jan. 2018). DOI: 10.1038/s41467-018-02825-9.

[33] Carlos Eduardo G. Amorim et al. "Understanding 6th-century barbarian social organization and migration through paleogenomics". In: *Nat Commun* 9.1 (Sept. 2018). DOI: 10.1038/s41467-018-06024-4.

[34] Cristina Valdiosera et al. "Four millennia of Iberian biomolecular prehistory illustrate the impact of prehistoric migrations at the far end of Eurasia". In: *Proc. Natl. Acad. Sci. U.S.A.* 115.13 (Mar. 2018), pp. 3428–3433. DOI: 10.1073/pnas.1717762115.

[35] D. M. Fernandes et al. “A genomic Neolithic time transect of hunter-farmer admixture in central Poland”. In: *Sci Rep* 8.1 (Oct. 2018). DOI: 10.1038/s41598-018-33067-w.

[36] Iñigo Olalde et al. “The Beaker phenomenon and the genomic transformation of northwest Europe”. In: *Nature* 555.7695 (Feb. 2018), pp. 190–196. DOI: 10.1038/nature25738.

[37] Iain Mathieson et al. “The genomic history of southeastern Europe”. In: *Nature* 555.7695 (Feb. 2018), pp. 197–203. DOI: 10.1038/nature25778.

[38] Krishna R. Veeramah et al. “Population genomic analysis of elongated skulls reveals extensive female-biased immigration in Early Medieval Bavaria”. In: *Proc. Natl. Acad. Sci. U.S.A.* 115.13 (Mar. 2018), pp. 3494–3499. DOI: 10.1073/pnas.1719880115.

[39] Maja Krzewińska et al. “Genomic and Strontium Isotope Variation Reveal Immigration Patterns in a Viking Age Town”. In: *Current Biology* 28.17 (Sept. 2018), 2730–2738.e10. DOI: 10.1016/j.cub.2018.06.053.

[40] Maja Krzewińska et al. “Ancient genomes suggest the eastern Pontic-Caspian steppe as the source of western Iron Age nomads”. In: *Sci. Adv.* 4.10 (Oct. 2018). DOI: 10.1126/sciadv.aat4457.

[41] Niall O’Sullivan et al. “Ancient genome-wide analyses infer kinship structure in an Early Medieval Alemannic graveyard”. In: *Sci. Adv.* 4.9 (Sept. 2018). DOI: 10.1126/sciadv.aao1262.

[42] Peter de Barros Damgaard et al. “137 ancient human genomes from across the Eurasian steppes”. In: *Nature* 557.7705 (May 2018), pp. 369–374. DOI: 10.1038/s41586-018-0094-2.

[43] Peter de Barros Damgaard et al. “The first horse herders and the impact of early Bronze Age steppe expansions into Asia”. In: *Science* 360.6396 (June 2018). DOI: 10.1126/science.aar7711.

[44] Pierre Zalloua et al. “Ancient DNA of Phoenician remains indicates discontinuity in the settlement history of Ibiza”. In: *Sci Rep* 8.1 (Dec. 2018). DOI: 10.1038/s41598-018-35667-y.

[45] Thiseas C. Lamnidis et al. “Ancient Fennoscandian genomes reveal origin and spread of Siberian ancestry in Europe”. In: *Nat Commun* 9.1 (Nov. 2018). DOI: 10.1038/s41467-018-07483-5.

[46] Torsten Günther et al. “Population genomics of Mesolithic Scandinavia: Investigating early postglacial migration routes and high-latitude adaptation”. In: *PLoS Biol* 16.1 (Jan. 2018). Ed. by Nick Barton, e2003703. DOI: 10.1371/journal.pbio.2003703.

[47] Alissa Mittnik et al. “Kinship-based social inequality in Bronze Age Europe”. In: *Science* 366.6466 (Nov. 2019), pp. 731–734. DOI: 10.1126/science.aax6219.

[48] Christiana L. Scheib et al. “East Anglian early Neolithic monument burial linked to contemporary Megaliths”. In: *Annals of Human Biology* 46.2 (Feb. 2019), pp. 145–149. DOI: 10.1080/03014460.2019.1623912.

[49] Chuan-Chao Wang et al. “Ancient human genome-wide data from a 3000-year interval in the Caucasus corresponds with eco-geographic regions”. In: *Nat Commun* 10.1 (Feb. 2019). DOI: 10.1038/s41467-018-08220-8.

[50] Federico Sánchez-Quinto et al. “Megalithic tombs in western and northern Neolithic Europe were linked to a kindred society”. In: *Proc. Natl. Acad. Sci. U.S.A.* 116.19 (Apr. 2019), pp. 9469–9474. DOI: 10.1073/pnas.1818037116.

[51] G. González-Fortes et al. “A western route of prehistoric human migration from Africa into the Iberian Peninsula”. In: *Proc. R. Soc. B.* 286.1895 (Jan. 2019), p. 20182288. DOI: 10.1098/rspb.2018.2288.

[52] Hannes Schroeder et al. "Unraveling ancestry, kinship, and violence in a Late Neolithic mass grave". In: *Proc. Natl. Acad. Sci. U.S.A.* 116.22 (May 2019), pp. 10705–10710. DOI: 10.1073/pnas.1820210116.

[53] Helena Malmström et al. "The genomic ancestry of the Scandinavian Battle Axe Culture people and their relation to the broader Corded Ware horizon". In: *Proc. R. Soc. B.* 286.1912 (Oct. 2019), p. 20191528. DOI: 10.1098/rspb.2019.1528.

[54] Iñigo Olalde et al. "The genomic history of the Iberian Peninsula over the past 8000 years". In: *Science* 363.6432 (Mar. 2019), pp. 1230–1234. DOI: 10.1126/science.aav4040.

[55] Lehti Saag et al. "The Arrival of Siberian Ancestry Connecting the Eastern Baltic to Uralic Speakers further East". In: *Current Biology* 29.10 (May 2019), 1701–1711.e16. DOI: 10.1016/j.cub.2019.04.026.

[56] Marc Haber et al. "A Transient Pulse of Genetic Admixture from the Crusaders in the Near East Identified from Ancient Genome Sequences". In: *The American Journal of Human Genetics* 104.5 (May 2019), pp. 977–984. DOI: 10.1016/j.ajhg.2019.03.015.

[57] Margaret L. Antonio et al. "Ancient Rome: A genetic crossroads of Europe and the Mediterranean". In: *Science* 366.6466 (Nov. 2019), pp. 708–714. DOI: 10.1126/science.aay6826.

[58] Mari Järve et al. "Shifts in the Genetic Landscape of the Western Eurasian Steppe Associated with the Beginning and End of the Scythian Dominance". In: *Current Biology* 29.14 (July 2019), 2430–2441.e10. DOI: 10.1016/j.cub.2019.06.019.

[59] Martin Sikora et al. "The population history of northeastern Siberia since the Pleistocene". In: *Nature* 570.7760 (June 2019), pp. 182–188. DOI: 10.1038/s41586-019-1279-z.

[60] Michal Feldman et al. "Ancient DNA sheds light on the genetic origins of early Iron Age Philistines". In: *Sci. Adv.* 5.7 (July 2019). DOI: 10.1126/sciadv.aax0061.

[61] Michal Feldman et al. "Late Pleistocene human genome suggests a local origin for the first farmers of central Anatolia". In: *Nat Commun* 10.1 (Mar. 2019). DOI: 10.1038/s41467-019-09209-7.

[62] Selina Brace et al. "Ancient genomes indicate population replacement in Early Neolithic Britain". In: *Nat Ecol Evol* 3.5 (Apr. 2019), pp. 765–771. DOI: 10.1038/s41559-019-0871-9.

[63] Vagheesh M. Narasimhan et al. "The formation of human populations in South and Central Asia". In: *Science* 365.6457 (Sept. 2019). DOI: 10.1126/science.aat7487.

[64] Vanessa Villalba-Mouco et al. "Survival of Late Pleistocene Hunter-Gatherer Ancestry in the Iberian Peninsula". In: *Current Biology* 29.7 (Apr. 2019), 1169–1177.e7. DOI: 10.1016/j.cub.2019.02.006.

[65] Alexandra Coutinho et al. "The Neolithic Pitted Ware culture foragers were culturally but not genetically influenced by the Battle Axe culture herders". In: *Am J Phys Anthropol* 172.4 (June 2020), pp. 638–649. DOI: 10.1002/ajpa.24079.

[66] Anja Furtwängler et al. "Comparison of target enrichment strategies for ancient pathogen DNA". In: *BioTechniques* 69.6 (Dec. 2020), pp. 455–459. DOI: 10.2144/btn-2020-0100.

[67] Anna Linderholm et al. "Corded Ware cultural complexity uncovered using genomic and isotopic analysis from south-eastern Poland". In: *Sci Rep* 10.1 (Apr. 2020). DOI: 10.1038/s41598-020-63138-w.

[68] Ashot Margaryan et al. "Population genomics of the Viking world". In: *Nature* 585.7825 (Sept. 2020), pp. 390–396. DOI: 10.1038/s41586-020-2688-8.

[69] Daniel M. Fernandes et al. "The spread of steppe and Iranian-related ancestry in the islands of the western Mediterranean". In: *Nat Ecol Evol* 4.3 (Feb. 2020), pp. 334–345. DOI: 10.1038/s41559-020-1102-0.

[70] David Gokhman et al. "Differential DNA methylation of vocal and facial anatomy genes in modern humans". In: *Nat Commun* 11.1 (Mar. 2020). DOI: 10.1038/s41467-020-15020-6.

[71] Eirini Skourtanioti et al. "Genomic History of Neolithic to Bronze Age Anatolia, Northern Levant, and Southern Caucasus". In: *Cell* 181.5 (May 2020), 1158–1175.e28. DOI: 10.1016/j.cell.2020.04.044.

[72] Joachim Burger et al. "Low Prevalence of Lactase Persistence in Bronze Age Europe Indicates Ongoing Strong Selection over the Last 3,000 Years". In: *Current Biology* 30.21 (Nov. 2020), 4307–4315.e13. DOI: 10.1016/j.cub.2020.08.033.

[73] Joseph H. Marcus et al. "Genetic history from the Middle Neolithic to present on the Mediterranean island of Sardinia". In: *Nat Commun* 11.1 (Feb. 2020). DOI: 10.1038/s41467-020-14523-6.

[74] Lara M. Cassidy et al. "A dynastic elite in monumental Neolithic society". In: *Nature* 582.7812 (June 2020), pp. 384–388. DOI: 10.1038/s41586-020-2378-6.

[75] Lily Agranat-Tamir et al. "The Genomic History of the Bronze Age Southern Levant". In: *Cell* 181.5 (May 2020), 1146–1157.e11. DOI: 10.1016/j.cell.2020.04.024.

[76] Maïté Rivollat et al. "Ancient genome-wide DNA from France highlights the complexity of interactions between Mesolithic hunter-gatherers and Neolithic farmers". In: *Sci. Adv.* 6.22 (May 2020). DOI: 10.1126/sciadv.aaz5344.

[77] Marc Haber et al. "A Genetic History of the Near East from an aDNA Time Course Sampling Eight Points in the Past 4,000 Years". In: *The American Journal of Human Genetics* 107.1 (July 2020), pp. 149–157. DOI: 10.1016/j.ajhg.2020.05.008.

[78] Samantha Brunel et al. "Ancient genomes from present-day France unveil 7,000 years of its demographic history". In: *Proc. Natl. Acad. Sci. U.S.A.* 117.23 (May 2020), pp. 12791–12798. DOI: 10.1073/pnas.1918034117.

[79] Éadaoin Harney et al. "A minimally destructive protocol for DNA extraction from ancient teeth". In: *Genome Res.* 31.3 (Feb. 2021), pp. 472–483. DOI: 10.1101/gr.267534.120.

[80] Andaine Seguin-Orlando et al. "Heterogeneous Hunter-Gatherer and Steppe-Related Ancestries in Late Neolithic and Bell Beaker Genomes from Present-Day France". In: *Current Biology* 31.5 (Mar. 2021), 1072–1083.e10. DOI: 10.1016/j.cub.2020.12.015.

[81] Anne Friis-Holm Egfjord et al. "Genomic Steppe ancestry in skeletons from the Neolithic Single Grave Culture in Denmark". In: *PLoS ONE* 16.1 (Jan. 2021). Ed. by Peter F. Biehl, e0244872. DOI: 10.1371/journal.pone.0244872.

[82] Guido Alberto Gnecchi-Ruscone et al. "Ancient genomic time transect from the Central Asian Steppe unravels the history of the Scythians". In: *Sci. Adv.* 7.13 (Mar. 2021). DOI: 10.1126/sciadv.abe4414.

[83] Lehti Saag et al. "Genetic ancestry changes in Stone to Bronze Age transition in the East European plain". In: *Sci. Adv.* 7.4 (Jan. 2021). DOI: 10.1126/sciadv.abd6535.

[84] Luka Papac et al. "Dynamic changes in genomic and social structures in third millennium BCE central Europe". In: *Sci. Adv.* 7.35 (Aug. 2021). DOI: 10.1126/sciadv.abi6941.

[85] Mario Novak et al. "Genome-wide analysis of nearly all the victims of a 6200 year old massacre". In: *PLoS ONE* 16.3 (Mar. 2021). Ed. by Peter F. Biehl, e0247332. DOI: 10.1371/journal.pone.0247332.

[86] Reyhan Yaka et al. "Variable kinship patterns in Neolithic Anatolia revealed by ancient genomes". In: *Current Biology* 31.11 (June 2021), 2455–2468.e18. DOI: 10.1016/j.cub.2021.03.050.
